# Evaluation of Alisertib Alone or Combined With Fulvestrant in Patients With Endocrine-Resistant Advanced Breast Cancer: The Phase 2 TBCRC041 Randomized Clinical Trial

**DOI:** 10.1001/jamaoncol.2022.7949

**Published:** 2023-03-09

**Authors:** Tufia C. Haddad, Vera J. Suman, Antonino B. D’Assoro, Jodi M. Carter, Karthik V. Giridhar, Brendan P. McMenomy, Katelyn Santo, Erica L. Mayer, Meghan S. Karuturi, Aki Morikawa, P. Kelly Marcom, Claudine J. Isaacs, Sun Young Oh, Amy S. Clark, Ingrid A. Mayer, Khandan Keyomarsi, Timothy J. Hobday, Prema P. Peethambaram, Ciara C. O’Sullivan, Roberto A. Leon-Ferre, Minetta C. Liu, James N. Ingle, Matthew P. Goetz

**Affiliations:** 1 Department of Oncology, Mayo Clinic, Rochester, Minnesota; 2 Department of Quantitative Health Sciences, Mayo Clinic, Rochester, Minnesota; 3 Department of Laboratory Medicine and Pathology, Mayo Clinic, Rochester, Minnesota; 4 Department of Radiology, Mayo Clinic, Rochester, Minnesota; 5 Department of Medical Oncology, Dana-Farber Cancer Institute, Boston, Massachusetts; 6 Department of Breast Medical Oncology, MD Anderson Cancer Center, Houston, Texas; 7 Department of Medicine, University of Michigan, Ann Arbor; 8 Department of Medicine, Duke University Cancer Institute, Durham, North Carolina; 9 Department of Medicine, Georgetown University, Washington, DC; 10 Department of Medical Oncology, Albert Einstein College of Medicine/Montefiore Medical Center, Bronx, New York; 11 Department of Medicine, University of Pennsylvania, Philadelphia; 12 Department of Medicine, Vanderbilt University Medical Center, Nashville, Tennessee; 13 Department of Experimental Radiation Oncology, MD Anderson Cancer Center, Houston, Texas

## Abstract

**Importance:**

Aurora A kinase (AURKA) activation, related in part to *AURKA* amplification and variants, is associated with downregulation of estrogen receptor (ER) α expression, endocrine resistance, and implicated in cyclin-dependent kinase 4/6 inhibitor (CDK 4/6i) resistance. Alisertib, a selective AURKA inhibitor, upregulates ERα and restores endocrine sensitivity in preclinical metastatic breast cancer (MBC) models. The safety and preliminary efficacy of alisertib was demonstrated in early-phase trials; however, its activity in CDK 4/6i–resistant MBC is unknown.

**Objective:**

To assess the effect of adding fulvestrant to alisertib on objective tumor response rates (ORRs) in endocrine-resistant MBC.

**Design, Setting, and Participants:**

This phase 2 randomized clinical trial was conducted through the Translational Breast Cancer Research Consortium, which enrolled participants from July 2017 to November 2019. Postmenopausal women with endocrine-resistant, ERBB2 (formerly HER2)–negative MBC who were previously treated with fulvestrant were eligible. Stratification factors included prior treatment with CDK 4/6i, baseline metastatic tumor ERα level measurement (<10%, ≥10%), and primary or secondary endocrine resistance. Among 114 preregistered patients, 96 (84.2%) registered and 91 (79.8%) were evaluable for the primary end point. Data analysis began after January 10, 2022.

**Interventions:**

Alisertib, 50 mg, oral, daily on days 1 to 3, 8 to 10, and 15 to 17 of a 28-day cycle (arm 1) or alisertib same dose/schedule with standard-dose fulvestrant (arm 2).

**Main Outcomes and Measures:**

Improvement in ORR in arm 2 of at least 20% greater than arm 1 when the expected ORR for arm 1 was 20%.

**Results:**

All 91 evaluable patients (mean [SD] age, 58.5 [11.3] years; 1 American Indian/Alaskan Native [1.1%], 2 Asian [2.2%], 6 Black/African American [6.6%], 5 Hispanic [5.5%], and 79 [86.8%] White individuals; arm 1, 46 [50.5%]; arm 2, 45 [49.5%]) had received prior treatment with CDK 4/6i. The ORR was 19.6%; (90% CI, 10.6%-31.7%) for arm 1 and 20.0% (90% CI, 10.9%-32.3%) for arm 2. In arm 1, the 24-week clinical benefit rate and median progression-free survival time were 41.3% (90% CI, 29.0%-54.5%) and 5.6 months (95% CI, 3.9-10.0), respectively, and in arm 2 they were 28.9% (90% CI, 18.0%-42.0%) and 5.4 months (95% CI, 3.9-7.8), respectively. The most common grade 3 or higher adverse events attributed to alisertib were neutropenia (41.8%) and anemia (13.2%). Reasons for discontinuing treatment were disease progression (arm 1, 38 [82.6%]; arm 2, 31 [68.9%]) and toxic effects or refusal (arm 1, 5 [10.9%]; arm 2, 12 [26.7%]).

**Conclusions and Relevance:**

This randomized clinical trial found that adding fulvestrant to treatment with alisertib did not increase ORR or PFS; however, promising clinical activity was observed with alisertib monotherapy among patients with endocrine-resistant and CDK 4/6i–resistant MBC. The overall safety profile was tolerable.

**Trial Registration:**

ClinicalTrials.gov Identifier: NCT02860000

## Key Points

**Question:**

Does treatment with alisertib restore fulvestrant sensitivity and improve tumor objective response rates (ORRs) compared with alisertib monotherapy in patients with endocrine-resistant metastatic breast cancer?

**Findings:**

In this phase 2 randomized clinical trial of 91 patients with endocrine-resistant, metastatic breast cancer who were previously treated with a cyclin-dependent kinase 4/6 inhibitor, participants were randomized to receive treatment with alisertib alone or combined with fulvestrant. The ORR was not significantly improved by the addition of fulvestrant to alisertib, with an ORR of approximately 20.0% for both regimens.

**Meaning:**

The trial results found that while alisertib did not restore fulvestrant sensitivity and increase ORRs, promising clinical activity was observed with alisertib monotherapy among patients with endocrine-resistant and cyclin-dependent kinase 4/6 inhibitor–resistant metastatic breast cancer.

## Teaser

This randomized clinical trial examines the effect of adding fulvestrant to alisertib on objective tumor response rates in endocrine-resistant metastatic breast cancer.

## Introduction

Effective approaches to overcome endocrine therapy (ET) resistance remain a major challenge in estrogen receptor (ER)–positive breast cancer management.^1^ Loss of ERα expression, a mechanism of ET resistance, is associated with tumor progression and poor clinical outcomes.^1,2,3,4^

We have previously demonstrated in luminal ER^+^ breast cancer models that Aurora A kinase (AURKA) activation induces epithelial to mesenchymal transition reprogramming and clonal expansion of CD44^+^/CD24^low/−^ cells with stemness features and the ability to self-renew and propagate distant metastasis.^5^ Furthermore, these cells are characterized by loss of ERα expression and resistance to ET.^6^ In tamoxifen-resistant ER^+^ breast cancer models, the selective AURKA inhibitor, alisertib, was found to restore a CD44^−^/CD24^+^ epithelial phenotype, ERα expression, and sensitivity to ET.^5,6^ AURKA further emerged as a novel therapeutic target in kinase inhibitor screens given its association with ET resistance^7,8^ and shorter disease-free and overall survival (OS) when expressed at high levels in ER^+^ tumor biospecimens.^9^

Alisertib’s safety and tolerability profile was well-defined in early phase trials in hematologic cancers.^10,11,12^ In a phase 2 basket trial of alisertib monotherapy for solid tumors, among those with heavily pretreated, ER^+^/ERBB2^−^ (formerly HER2) metastatic breast cancer (MBC) (n = 26), a 23% objective tumor response rate (ORR), 6-month clinical benefit rate (CBR) of 54%, and median progression-free survival (mPFS) of 7.9 months were observed.^13^

Based on our preclinical rationale, we conducted a phase 1 trial combining alisertib with fulvestrant in patients who previously received aromatase inhibitor (AI) therapy for ER^+^/ERBB2^−^ MBC.^14^ A 28-day, pulse dose alisertib regimen^15^ was used to align with the fulvestrant schedule and reduce myelosuppression. A favorable safety profile and promising clinical activity was observed, with a 6-month CBR of 78% and mPFS of 12.4 months in this cyclin-dependent kinase 4/6 inhibitor (CDK 4/6i)–naive cohort.

Concurrent with alisertib development for MBC, the CDK 4/6is were established as optimal first or second-line therapies in combination with ET for ER^+^/ERBB2^−^ MBC.^16^ Despite substantial clinical improvements gained by this targeted treatment approach, CDK 4/6i resistance eventually ensues, and subsequent treatment with an AI or fulvestrant has been associated with a limited mPFS of 1.9 months in this setting.^17^ Optimal treatment after CDK4/6i progression remains poorly defined. AURKA amplification and variants have been associated with resistance to CDK 4/6i.^18,19^ However, as prior trials with alisertib did not include CDK 4/6i–resistant patients, the safety and efficacy of alisertib in this setting was unknown.

Given the phase 1 results and potential importance of AURKA in the setting of endocrine and CDK 4/6i resistance, we conducted an investigator-initiated, multicenter, randomized phase 2 clinical trial to evaluate alisertib alone or combined with fulvestrant in patients with endocrine-resistant, ERBB2^−^ MBC. The primary objective of this study was to determine if the addition of fulvestrant to treatment with alisertib could improve ORRs in fulvestrant-resistant disease.

## Methods

### Study Approval and Patient Eligibility

This protocol was approved by the Mayo Clinic institutional review board and each site institutional review board within the participating Translational Breast Cancer Research Consortium (TBCRC) institutions (Supplement 1). Participants provided written informed consent. Postmenopausal women with ER^+^/ERBB2^−^ MBC or ER^−^/ERBB2^−^ MBC with a history of primary ER^+^/ERBB2^−^ disease were preregistered. The study defined ER^+^ disease (by clinical assay) as 10% or more cells positive to limit inclusion of basal-like ER^−^/ERBB2^−^ MBC that did not acquire ET resistance due to ERα downregulation. Additional key preregistration criteria included 2 or fewer prior MBC chemotherapy regimens, prior treatment with fulvestrant for ER^+^ MBC, measurable disease by Response Evaluation Criteria in Solid Tumors (RECIST) criteria, willingness to limit daily alcohol intake, and no visceral crisis. Unlimited prior treatment with ET was allowed.

During the preregistration period, a biopsy specimen of a metastatic site was obtained for central ERα testing (for stratification). Key registration criteria included adequate blood cell counts and chemistry results, an Eastern Cooperative Oncology Group (ECOG) performance score of 0 to 1, no systemic therapy 21 days or fewer before registration, no need for treatment with a chronic proton pump inhibitor or H2 antagonist, and no visceral crisis.

### Study Design

#### Registration and Stratification

Patients were randomized 1:1 to arm 1 (alisertib) or arm 2 (alisertib and fulvestrant). Treatment was assigned using Pocock-Simon dynamic allocation procedure^20^ with stratification factors: ET resistance (primary vs secondary),^21^ central laboratory ERα expression (positive vs negative), and prior treatment with CDK 4/6i (yes vs no).

#### Treatment Schedule

Alisertib was administered as 50 mg, oral, twice daily on days 1 to 3, 8 to 10, and 15 to 17 of a 28-day cycle. Fulvestrant was administered as 500 mg, intramuscularly on days 1 and 15 of a 28-day cycle (cycle 1) and then day 1 of all subsequent cycles.

Arm 1 patients experiencing progression of disease (PD) could crossover to arm 2 if (1) ER was 10%or greater positive in preregistration or PD tumor tissue; (2) recovery from toxic effects to grade 0 to 1; (3) adequate blood cell counts and chemistry results; and (4) treatment initiated within 35 days of PD.

#### Patient Safety and Efficacy Evaluations

Adverse events (AEs) were documented using Common Terminology Criteria for Adverse Events, version 4. Alisertib dose levels/modifications are provided in eTable 1 in Supplement 2. Fulvestrant was not modified for AEs. If treatment with alisertib was paused due to toxic effects, then so was fulvestrant.

Within 14 days of registration, before each cycle, and at PD, patients underwent toxic effect assessments. Tumor assessments were performed after every 2 cycles.

### Correlative Tissue and Blood Biospecimens

Metastatic tumor biospecimens were collected during preregistration, end of cycle 1, and PD. Two tissue cores were formalin fixed and paraffin embedded while 1 core was immediately frozen at −70 °F. Formalin-fixed and paraffin-embedded tissues were sectioned at 5 microns and immunohistochemistry (IHC) staining performed using the ERα antibody, prediluted, clone SP1 (Ventana) and total AURKA antibody, diluted 1:100, clone JLM28 (Leica Microsystems).

### Statistical Design and Analysis Plan

A 2-stage, randomized phase 2 clinical trial design^22^ with a prospective control treatment and futility stopping rule was used to assess whether the ORR with the addition of fulvestrant to alisertib was greater than alisertib alone by at least 20%, assuming the ORR for alisertib is 20%.^13^ The ORR was defined as the percentage of patients with a complete or partial response by RECIST criteria on 2 consecutive evaluations at least 8 weeks apart. In the first stage, 28 patients were randomized to each arm, and if the combination therapy had 1 or fewer tumor responses than alisertib monotherapy, then the trial was closed to enrollment. Otherwise, an additional 17 patients were randomized to each arm. If there were 4 or more tumor responses among 45 patients who were receiving combination therapy, then we concluded that the ORR in that arm was at least 20% greater than with alisertib alone. The decision rules were chosen so that type 1 and 2 errors were 0.15. The type I error rate was relaxed for ORR and 24-week CBR as the goal was to uncover signals of benefit in support of further testing in this patient population. All registered, eligible patients who began treatment were included in the analysis cohorts according to their assigned arm.

#### Secondary End Points

The 24-week CBR was defined as the percentage of patients who completed 6 cycles of treatment without PD. A 90% binomial confidence interval of the CBR was constructed. The duration of response (DoR) was measured from the date of complete or partial response to the date of PD or death, whichever was earlier. Progression-free survival was defined as time from randomization to PD or death. Overall survival was defined as time from randomization to any-cause death. The distributions of PFS and OS times were estimated with the Kaplan-Meier method.

#### Correlative End Points

Preregistration metastatic tumor biopsy specimens were evaluated for AURKA expression (negative expression defined as 0%-10% of tumor cells with IHC staining present) and ERα expression (negative expression defined as <10% of tumor cells with IHC staining present). Cox modeling was used to explore whether PFS differed in either treatment arm regarding preregistration tumor AURKA expression or ERα expression.

#### Analysis

Data lock occurred on January 10, 2022. Analyses were conducted using SAS, version 9.4 (SAS Institute).

### Study Monitoring

The Mayo Clinic Comprehensive Cancer Center data safety and monitoring board monitored the trial every 6 months. The safety stopping rules applied to each arm separately. If 3 or more of the first 10 patients or 30% or more of patients randomized to the arm thereafter developed a grade 4 toxic effect that was possibly, probably, or definitely related to treatment, then enrollment would be temporarily suspended until the study chair and biostatistician recommendations were approved by coinvestigators.

## Results

The trial activated at the Mayo Clinic on July 6, 2017, and at the 8 participating TBCRC sites beginning February 19, 2018. Enrollment was suspended April 2, 2018, after tolerability issues were identified. Five of the initial 12 patients had grade 4 or higher AEs, among which 4 were attributed as at least possibly related to alisertib (neutropenia [2], hemolysis [1], and acute respiratory failure [1]). In response, eligibility criteria were modified to limit those with end-organ damage, central nervous system metastasis, or an ECOG performance score greater than 1 from participating (eTable 2 in Supplement 2). The study reopened to enrollment from June 13, 2018, to November 27, 2019, during which no major safety issues were encountered and accrual goals were met.

### Trial Cohort

**Figure 1.**
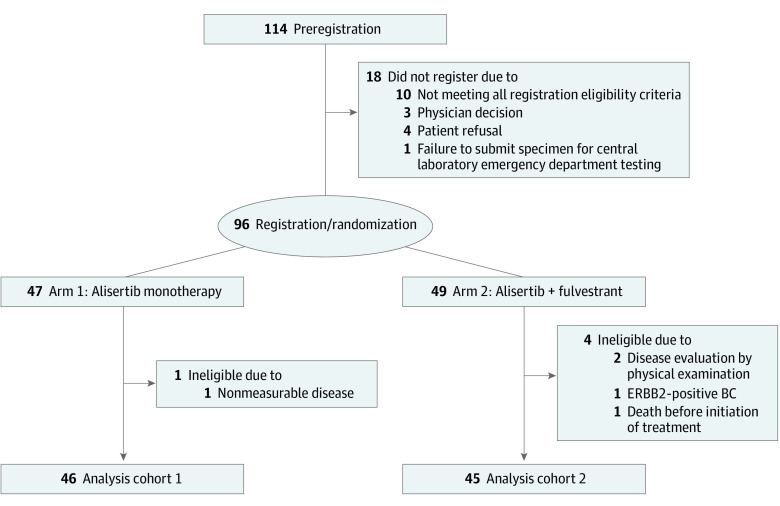
Consolidated Standards of Reporting Trials Diagram Patient flow from preregistration through registration/randomization and creation of the analytic cohort. BC indicates breast cancer.

Among 114 patients who preregistered, 18 (15.8%) did not proceed to registration (Figure 1). Of 96 patients registered, 5 (5.2%) were nonevaluable for the primary end point. The remaining 91 patients comprised the analysis cohort. Patient and disease characteristics are presented in Table 1. Of note, 91 (100%) received prior treatment with CDK 4/6i, 53 (58.2%) received prior chemotherapy for MBC, and all those with ER^+^ MBC received prior treatment with fulvestrant.

**Table 1.** Patient Characteristics

Characteristic	No. (%)	
	Arm 1: alisertib (n = 46)	Arm 2: alisertib + fulvestrant (n = 45)
Age, y		
18-39	3 (6.5)	4 (8.9)
40-59	14 (30.4)	20 (44.4)
60-74	25 (54.4)	19 (42.2)
≥75	4 (8.7)	2 (4.4)
Race		
American Indian/Alaskan Native	0	1 (2.2)
Asian	2 (4.4)	0
Black/African American	3 (6.5)	3 (6.7)
White	40 (87.0)	39 (86.7)
Not reported	1 (2.2)	2 (4.4)
Hispanic ethnicity	1 (2.2)	4 (8.9)
BMI		
Underweight	0	1 (2.2)
Normal	14 (30.4)	21 (46.7)
Overweight	13 (28.3)	15 (33.3)
Obesity	19 (41.3)	8 (17.8)
ECOG performance status		
0	30 (65.2)	27 (60.0)
1	16 (34.8)	18 (40.0)
Any prior chemotherapy		
(Neo)adjuvant setting	27 (58.7)	27 (60.0)
Metastatic setting	22 (47.8)	31 (68.9)
Prior lines of chemotherapy in MBC		
0	24 (52.1)	14 (31.1)
1	9 (19.6)	19 (42.2)
2	13 (28.3)	12 (26.7)
Any prior endocrine therapy		
(Neo)adjuvant setting	31 (67.4)	29 (64.4)
Metastatic setting	46 (100)	45 (100)
Prior (neo)adjuvant endocrine therapy		
Anastrozole	16 (34.8)	15 (33.3)
Exemestane	4 (8.7)	5 (11.1)
Fulvestrant	7 (15.2)	2 (4.4)
Letrozole	10 (21.7)	6 (13.3)
Raloxifene	1 (2.2)	0
Tamoxifen	15 (32.6)	22 (48.9)
Prior MBC endocrine therapy		
Amcenestrant	1 (2.2)	0
Anastrozole	8 (17.4)	4 (8.9)
Exemestane	16 (34.8)	26 (57.8)
Fulvestrant	45 (97.8)	45 (100)
Letrozole	21 (45.7)	33 (73.3)
Tamoxifen	12 (26.1)	10 (22.2)
Prior CDK 4/6 inhibitor treatment	46 (100)	45 (100)
Prior everolimus treatment		
Yes	17 (37.0)	26 (57.8)
No	29 (63.0)	19 (42.2)
Endocrine resistance		
Primary	11 (23.9)	8 (17.8)
Secondary	35 (76.1)	37 (82.2)
Metastatic sites of disease		
Nonvisceral	14 (31.1)	10 (22.2)
Visceral	31 (68.9)	35 (77.8)
Metastatic tumor ERα expression		
Positive (≥10%)	32 (69.6)	31 (68.9)
Borderline (1%-9.9%)	3 (6.5)	4 (13.1)
Negative	5 (10.9)	3 (6.7)
Insufficient tissue	6 (13.0)	7 (15.6)

Abbreviations: BMI, body mass index (calculated as weight in kilograms divided by height in meters squared); CDK, cyclin-dependent kinase; ER, estrogen receptor; MBC, metastatic breast cancer.

### Safety and Efficacy Analysis

#### Arm 1: Alisertib

Three of 46 patients continued to receive alisertib as of the data lock. Reasons for discontinuing treatment included PD (38 [82.6%]), intolerability (2 [4.3%]), death (2 [4.3%]), and physician decision (1 [2.2%]). The median treatment cycles completed was 6 (range, 1-34). Seventeen patients (37.0%) required at least 1 dose reduction during the first year, most commonly due to neutropenia and anemia. The most common grade 3 or higher toxic effects were neutropenia (20 [43.4%]), leukopenia (8 [17.4%]), and anemia (9 [19.6%]). Table 2 summarizes treatment-related toxic effects.

**Table 2.** Treatment-Related Adverse Events by Treatment Arm^a^

Adverse events	Arm^c^	No. (%)^b^		
		Grade 2	Grade 3	Grade 4
Neutrophil count decreased	1	7 (15)	11 (24)	8 (17)
	2	6 (13)	9 (20)	10 (22)
White blood cell decreased	1	13 (28)	6 (13)	2 (4)
	2	8 (18)	10 (22)	4 (9)
Alopecia	1	19 (41)	0	0
	2	18 (40)	0	0
Anemia	1	10 (22)	7 (15)	1 (2)
	2	10 (22)	4 (9)	0
Fatigue	1	11 (24)	0	0
	2	13 (29)	4 (9)	0
Lymphocyte count decreased	1	5 (11)	2 (4)	0
	2	5 (11)	6 (13)	0
Anorexia	1	7 (15)	0	0
	2	3 (7)	1 (2)	0
Platelet count decreased	1	2 (4)	2 (4)	1 (2)
	2	2 (4)	1 (2)	1 (2)
Mucositis oral	1	4 (9)	1 (2)	0
	2	1 (2)	1 (2)	0
Nausea	1	1 (2)	0	0
	2	3 (7)	1 (2)	0
Febrile neutropenia	1	0	1 (2)	0
	2	0	1 (2)	1 (2)
Thromboembolic event	1	0	0	0
	2	0	3 (7)	0
Acute coronary syndrome	1	0	0	1 (2)
	2	0	0	0
Investigations: thrombotic microangiopathic hemolysis	1	0	0	1 (2)
	2	0	0	0

^a^ One grade 5 respiratory failure that was at least possibly related to treatment occurred in arm 2.

^b^ All grade 3 to 4 adverse events at least possibly related to treatment and the most common grade 2 adverse events (>10% in at least 1 treatment arm) at least possibly related to treatment were included.

^c^ Arm 1 alisertib monotherapy and arm 2 alisertib + fulvestrant.

**Figure 2.**
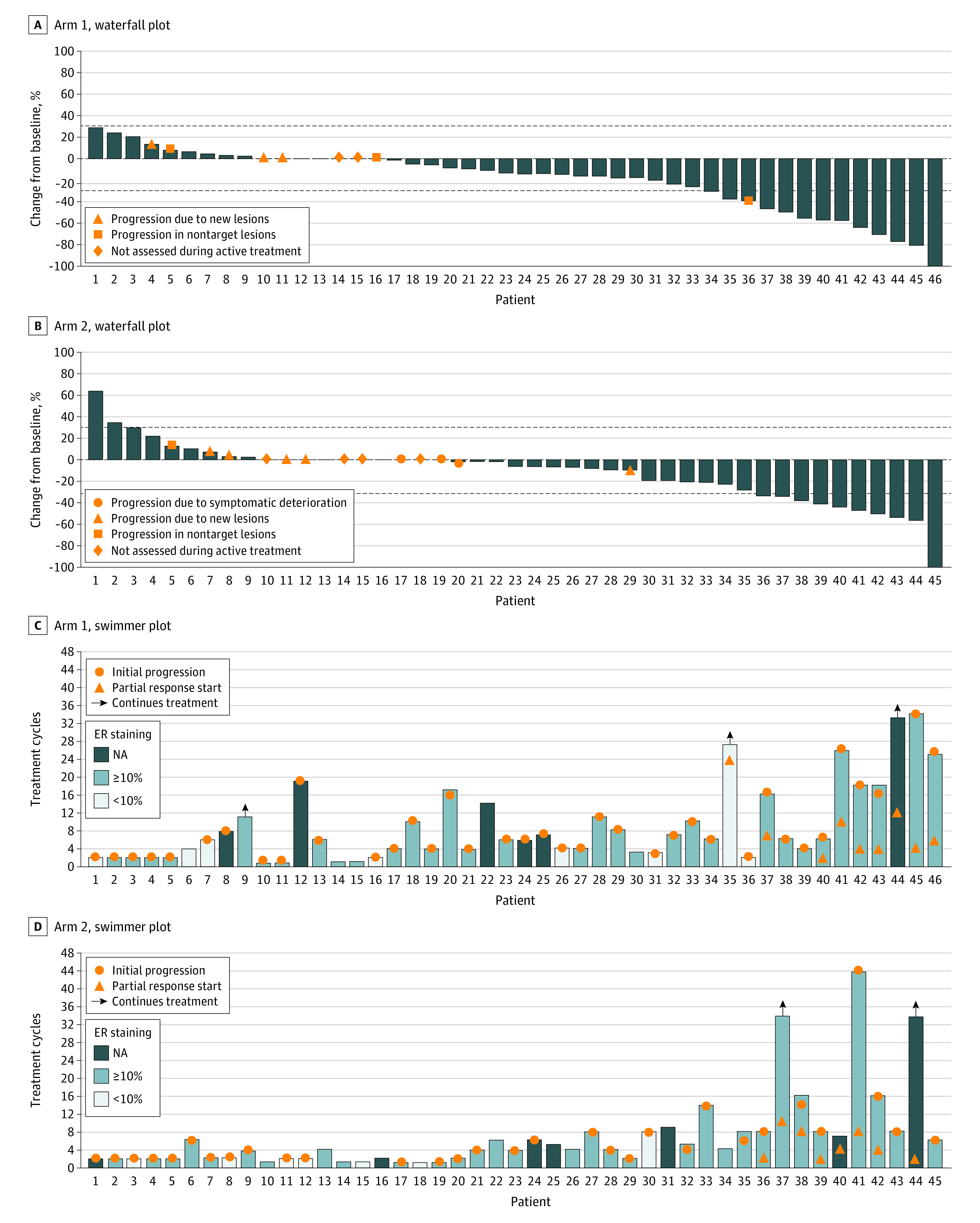
Best Overall Tumor Response and Disease Status Over Time for Individual Patients by Treatment Arm Waterfall plots indicate individual patient (x-axis) best tumor response as measured by percentage change from baseline (y-axis). Swimmer plots indicate individual disease status over time. Estrogen receptor (ER) α staining status is categorized by results of central review on the preregistration, metastatic site biopsy. A, Arm 1 (alisertib monotherapy) waterfall plot. B, Arm 2 (alisertib + fulvestrant) waterfall plot. C, Arm 1 (alisertib monotherapy) swimmer plot. D, Arm 2 (alisertib + fulvestrant) swimmer plot.

Nine partial responses were observed (Figure 2). Thus, the ORR was 19.6% (90% CI, 10.6%-31.7%). Median DoR was 15.1 months, and 24-week CBR was 41.3% (90% CI, 29.0%-54.5%). The mPFS time was estimated to be 5.6 months (95% CI, 3.9-10.0). The 1-year OS rate was 75.1% (95% CI, 63.4%-89.0%), and median OS time was estimated to be 22.7 months (95% CI, 18.4% to not estimable).

#### Arm 2: Alisertib + Fulvestrant

Two of 45 patients continued to receive alisertib with fulvestrant as of the data lock. Reasons for discontinuing treatment included PD (31 [68.9%]), intolerability (6 [13.3%]), refusal (4 [8.9%]), second primary cancer (1 [2.2%]), and death (1 [2.2%]). The median treatment cycles completed was 4 (range, 1-44). Sixteen patients (35.6%) required at least 1 dose reduction within the first year, most commonly due to neutropenia. Per Table 2, the most common grade 3 or higher toxic effects were neutropenia (19 [42.2%]), leukopenia (14 [31.1%]), lymphopenia (7 [15.6%]), fatigue (5 [11.1%]), and anemia (4 [8.9%]).

One complete response and 8 partial responses were observed (Figure 2). Thus, the ORR was 20.0%; (90% CI, 10.9%-32.3%). Median DoR was 8.5 months, and 24-week CBR was 28.9% (90% CI, 18.0%-42.0%). The estimated mPFS time was 5.4 months (95% CI, 3.9-7.8). The 1-year OS rate was 62.7% (95% CI, 49.7%-79.0%), and estimated median OS time was 19.8 months (95% CI, 11.5 to not estimable).

#### Arm 1 Crossover to Arm 2

Seventeen of 37 patients (45.9%) who experienced disease progression while taking alisertib crossed over to arm 2. This occurred mostly commonly after 2 (n = 5) or 6 cycles (n = 5) of alisertib; however, 4 patients initiated combination treatment after 17 to 26 cycles. All patients discontinued treatment due to PD after undergoing 1 to 14 cycles of combination therapy. One partial response was observed with combination treatment. The mPFS after crossover was 3.7 months.

### Correlative Studies

**Figure 3.**
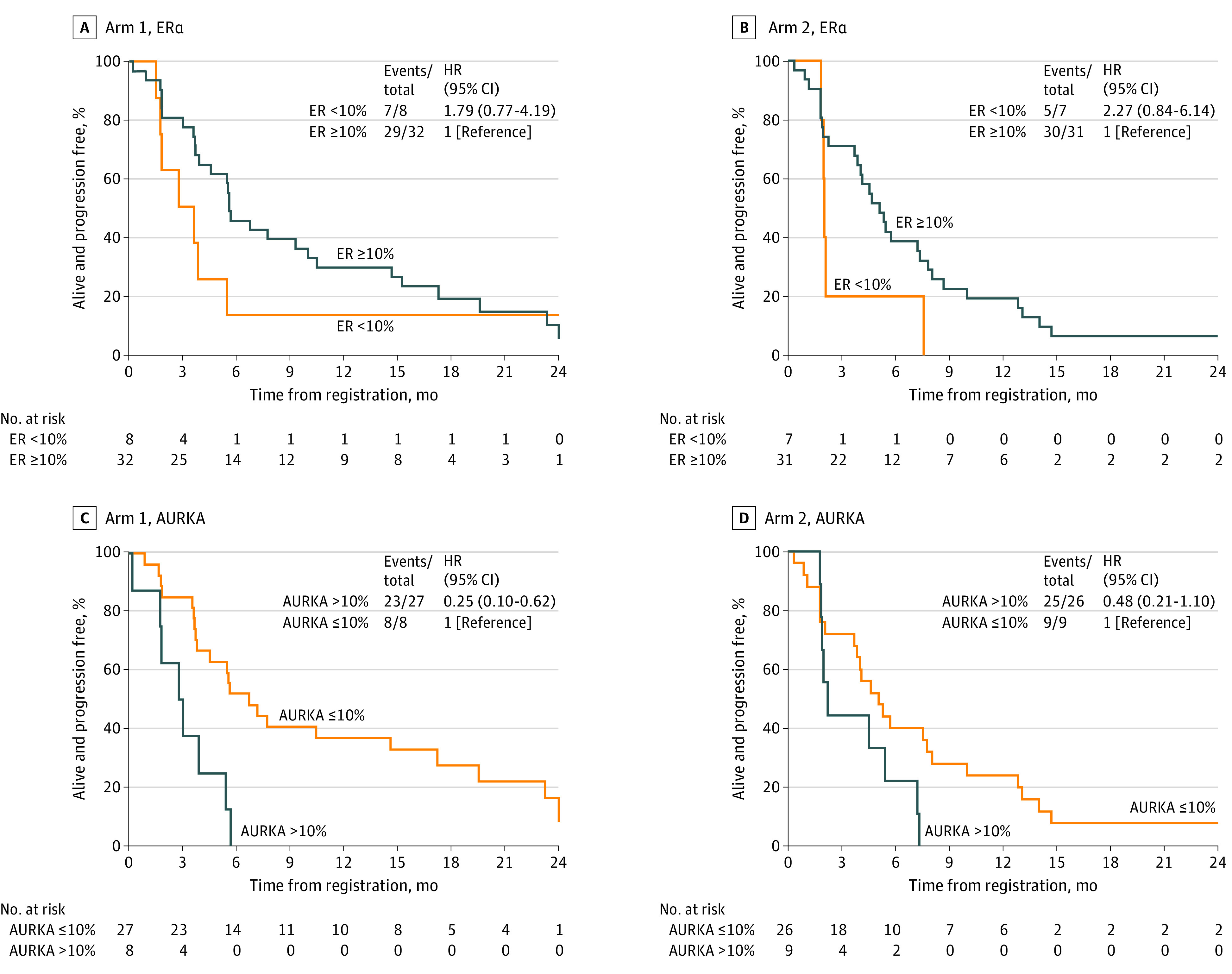
Association of Baseline Metastatic Tumor Estrogen Receptor (ER) α and Aurora A Kinase (AURKA) Expression and Progression-Free Survival (PFS) Formalin-fixed and paraffin-embedded metastatic tumor tissue blocks were sectioned and stained using antibodies against ERα or total AURKA. A log-rank test and Cox modeling were used to assess whether PFS differed regarding pretreatment metastatic tumor ERα expression of 10% or greater or AURKA expression less than 10%. A, Arm 1 (alisertib monotherapy): ERα. B, Arm 2 (alisertib + fulvestrant): ERα. C, Arm 1 (alisertib monotherapy): AURKA. D, Arm 2 (alisertib + fulvestrant): AURKA.

Expression levels of ERα and AURKA in the preregistration tumors were available for 70 patients (arm 1, 35; arm 2, 35) and 77 patients (arm 1, 39; arm 2, 38; Table 1), respectively. For arm 1, there was insufficient evidence to conclude that PFS differed regarding ERα expression (hazard ratio [HR], 1.79, 95% CI, 0.77%-4.19%; Figure 3A); however, there was evidence to suggest that PFS increased for those with AURKA negative tumors compared with those with AURKA positive tumors (HR, 0.25, 95% CI, 0.10-0.62; Figure 3C). For arm 2, there was insufficient evidence to conclude that PFS differed regarding either ERα expression (HR, 2.27, 95% CI, 0.84-6.14; Figure 3B) or AURKA expression (HR, 0.48, 95% CI, 0.21-1.10; Figure 3D).

## Discussion

In this randomized phase 2 clinical trial, the clinical efficacy and safety of alisertib alone or combined with fulvestrant was evaluated in ERBB2^−^ MBC. All patients received prior treatment with CDK 4/6i, and of those with ER^+^ MBC, all received prior fulvestrant. While the addition of fulvestrant to alisertib did not improve ORR, clinically meaningful antitumor activity was observed in both arms. Among those who received alisertib, this included a confirmed ORR of 19.6%, 24-week CBR of 41.3%, mPFS of 5.6 months, and median OS of 22.7 months.

These results confirm prior findings demonstrating the antitumor activity of alisertib in endocrine-resistant, ER^+^/ERBB2^−^ MBC. They are comparable with those observed in prior trials with established effective agents. For example, in MONARCH-1, a phase 2 study of the CDK 4/6i abemaciclib in patients with ER^+^ MBC that was previously treated with ET and 2 or fewer prior lines of chemotherapy, the confirmed ORR was 19.7% (95% CI, 13.3%-27.5%), 6-month CBR was 42.4%, mPFS was 6.0 months, and median OS was 17.7 months.^23^ These findings led to the US Food and Drug Administration approval of abemaciclib as monotherapy.

While caution must be applied when conducting cross-trial comparisons, to our knowledge, there is a paucity of clinical data regarding the efficacy of approved or investigational therapies following CDK 4/6i progression. Evaluating the current study results in this context may help to understand the potential value of alisertib in the evolving landscape of endocrine-resistant and CDK 4/6i–resistant MBC. Notably, single-agent ET has limited efficacy in this setting, as evidenced by the phase 2 VERONICA trial (n = 101) in which mPFS to fulvestrant was 1.94 months (95% CI, 1.84-3.55).^24^ In the phase 3 EMERALD trial, the oral selective ER degrader elacestrant was associated with a significantly improved mPFS compared with standard ET (fulvestrant or AI); however, mPFS was only 2.8 months.^17^

In the BYLieve trial, a single-arm study of alpelisib with fulvestrant in patients with CDK 4/6i–resistant, *PIK3CA*-variant, ER^+^ MBC, those with measurable disease (n = 100) had an ORR (calculated as best overall response) of 21% (95% CI, 14%-30%) and a 24-week CBR of 42% (95% CI, 35%-52%).^25^ In the overall cohort (n = 121), including those with the more clinically favorable nonmeasurable disease, mPFS was 7.3 months; (95% CI, 5.6-8.3) and mOS was 17.3 months (95% CI, 17.2-20.7). The duration of follow-up in BYLieve was limited, and thus the OS results may be immature. Additionally, *PIK3CA*-variant, ER^+^/ERBB2^−^ MBC is associated with shortened OS and resistance to chemotherapy compared with those with *PIK3CA* wild-type disease.^26^ While alisertib was associated with comparable clinical activity observed in the BYLieve trial, its efficacy in the setting of *PIK3CA*-variant disease is unknown and will be explored in subsequent correlative studies.

The phase 3 TROPICS-02 trial (n = 543) included patients with endocrine-resistant and CDK 4/6i–resistant MBC and reported an improvement in mPFS with the antibody drug conjugate sacituzumab govitecan (5.5 months; 95% CI, 4.2-7.0) compared with single-agent chemotherapy of the physician’s choice (4.0 months; 95% CI, 3.1-4.4).^27^ In the phase 3 DESTINY B-04 trial, among those in the analytic cohort with hormone receptor–positive/ERBB2 (HER2)^−^–low disease (n = 494), all patients were endocrine resistant, and 70.4% received prior treatment with CDK 4/6i. An improvement in mPFS was reported with the antibody drug conjugate trastuzumab deruxtecan compared with single-agent chemotherapy of the physician’s choice (10.1 vs 5.4 months; HR, 0.51, 95% CI, 0.4-0.64).^28^ Thus, the mPFS and other clinical outcomes associated with alisertib are similar to those observed in comparable patients treated with single-agent chemotherapy or sacituzumab govitecan.

An increased rate of investigational therapy discontinuation for reasons other than PD was observed in this trial among those receiving combination fulvestrant and alisertib. This was unexpected given the lack of overlapping toxic effects and favorable safety profile observed in our prior phase 1 trial of combination therapy.^14^ There were only minor differences in the most common AEs between arms. While there were more grade 3 fatigue and thromboembolic events in arm 2, they did not lead to treatment discontinuation. More patients in arm 2 than arm 1 received prior everolimus (57.8% vs 37.0%) and chemotherapy (68.9% vs 47.8%) for MBC. Thus, it is feasible that heavier pretreatment with other targeted and cytotoxic agents may have decreased tolerance to the investigational therapy. This is further strengthened by the observation that all 17 arm 1 patients who crossed over to arm 2 discontinued combination therapy due to PD, not treatment intolerance.

Given the clinical efficacy and safety results observed in TBCRC 041, continued clinical development of alisertib is warranted and planned. Further supporting this is the observation of clinical activity among those who received prior everolimus. If the clinical outcomes observed in this study were confirmed in a subsequent registration trial, alisertib may provide a new treatment option for endocrine-resistant disease to a broader cohort of patients given that alpelisib is only available for *PIK3CA*-variant MBC, which is present only in 25% to 40% of ER^+^/*ERBB2*^−^ MBC.^25,29^

The correlative studies indicated that high AURKA registration tumor expression was associated with poor clinical outcomes, which was consistent with prior studies.^9,30^ Additional correlative studies are under way to evaluate the role of *PIK3CA* variant status and assess whether pharmacodynamic-induced changes in ERα, total and phosphorylated AURKA, and other stemness biomarkers that are associated with clinical benefit from alisertib.

While alisertib did not restore sensitivity to fulvestrant as preclinical studies^5,6^ conducted before the CDK 4/6i era had suggested, it is feasible the hypothesis was not fairly tested given that accumulating clinical data demonstrate minimal antitumor activity of endocrine monotherapy following CDK 4/6i progression^17,24^ and the fact that the entire cohort received prior CDK 4/6i therapy. Furthermore, the role of continued ER blockade in combination with targeted therapies after CDK 4/6i progression is unclear.

### Limitations

Limitations to this trial include a higher percentage of women with obesity who were assigned to arm 1, while a higher percentage of women who had prior exposure to chemotherapy and/or everolimus were assigned to arm 2. Obesity, with its associated comorbidities, as well as prior exposure to chemotherapy, may have negatively affected tolerability to alisertib and the ability to continue receiving treatment. Additionally, the study cohort was confined to postmenopausal women, and participation of those of racial and ethnic minority groups was suboptimal, thus limiting generalizability. Finally, the sample size for the correlative studies was small, which limits interpretation.

## Conclusions

This phase 2 randomized clinical trial found that alisertib is among the first investigational targeted therapies demonstrating promising clinical activity and a tolerable safety profile in the setting of endocrine and CDK 4/6i–resistant MBC.

## Notes

Supplement 1. Trial protocol

Supplement 2. eTable 1. Dose levels and modifications for alisertib eTable 2. Safety stopping rule event summary and approved protocol changes

Supplement 3. Data sharing statement
