# Supplementary material for: Evaluation of Alisertib Alone or Combined With Fulvestrant in Patients With Endocrine-Resistant Advanced Breast Cancer: The Phase 2 TBCRC041 Randomized Clinical Trial
Source: JAMA Oncol. 2023 Mar 9;9(6):815–24. doi: 10.1001/jamaoncol.2022.7949 (PMC9999287; doi:10.1001/jamaoncol.2022.7949)
Supplement: Supplement 1. — Trial protocol [file jamaoncol-e227949-s001.pdf]

Randomized Phase II Trial to Evaluate Alisertib Alone or Combined with Fulvestrant for Women with Advanced, Endocrine-resistant Breast Cancer

Protocol Number	MC1431/TBCRC-041
Protocol Chair	Tufia C Haddad MD<br>Mayo Clinic<br>200 First Street SW<br>Rochester, MN 55905<br>507/284-2511
Co-Investigators	Antonio B D'Assoro MD PhD<br>Matthew P Goetz MD<br>John R Hawse PhD<br>Liewei Wang MD PhD<br>Minetta C Liu MD<br>Brendan McMenomy MD<br>Khandan Keyomarsi PhD<br>James N Ingle MD<br>Jodi M Carter MD PhD
Statistician	Vera J Suman, PhD
Supporters	Johns Hopkins University on behalf of the Translational Breast Cancer Research Consortium (TBCRC)<br>Takeda/Millennium (X14032)
Coordinating Center	Mayo Clinic<br>*Study Liaison*<br>Adrienne L Benson MBA<br>507/284-3045<br>mayotbcrc@mayo.edu

Randomized Phase II Trial to Evaluate Alisertib Alone or Combined with Fulvestrant for Women with Advanced, Endocrine-resistant Breast Cancer

- Protocol Revision Record -

**Original Protocol:**	08/19/2016 (Activation to Mayo Clinic)
Amendment 1:	01/23/2017
Amendment 2:	12/20/2017
Activation to TBCRC:	02/09/2018
Amendment 3:	06/13/2018
Amendment 4:	02/01/2019
Amendment 5:	02/25/2019
Amendment 6:	08/13/2019
Amendment 7:	03/17/2022
Amendment 8	07/06/2022

Protocol Resources

Questions:	Contact Name:
Patient eligibility*, test schedule, treatment delays/interruptions/adjustments, dose modifications, adverse events, forms completion and submission	Katie A Hervey<br>Data Manager<br>Phone: 507-266-6117<br>E-mail: hervey.katie@mayo.edu
Forms completion and submission (**Mayo Clinic only** – TBCRC sites contact Data Manager above)	Madilyn R Owen<br>Phone: 507-538-5843<br>Email: owen.madilyn@mayo.edu
Protocol document, consent form, regulatory issues	**TBCRC sites contact:**<br>Adrienne L Benson MBA<br>Phone: 507-284-3045<br>Email: mayotbcrc@mayo.edu<br><br>**Mayo Clinic sites contact:**<br>Lynn M Flickinger MA<br>Sr Research Protocol Specialist<br>Phone: 507-538-7034<br>Email: flickinger.lynn@mayo.edu
Paraffin-embedded tissue pathology	Amanda R Sand<br>Pathology Coordinator<br>Phone: 507-284-3559<br>E-mail: sand.amanda@mayo.edu
Non-paraffin biospecimens	Roxann M Neumann<br>Biospecimen Resource Manager<br>Phone: 507-538-0602<br>Email: neumann.roxann@mayo.edu
Serious Adverse Event Reporting	Serious Adverse Events Coordinator<br>E-mail: RSTP2CSAES@mayo.edu

*No waivers of eligibility will be granted

TABLE OF CONTENTS

RANDOMIZED PHASE II TRIAL TO EVALUATE ALISERTIB ALONE OR COMBINED WITH FULVESTRANT FOR WOMEN WITH ADVANCED, ENDOCRINE-RESISTANT BREAST CANCER 1

TABLE OF CONTENTS 4

SCHEMA 7

1. STUDY DESIGN/SUMMARY 8

2. OBJECTIVES 9

2.1 PRIMARY OBJECTIVE 9
2.2 SECONDARY OBJECTIVES 9
2.3 CORRELATIVE RESEARCH 9
2.4 EXPLORATORY OBJECTIVES 9

3. BACKGROUND 10

3.1 SCOPE OF THE PROBLEM OF HORMONE REFRACTORY ADVANCED BREAST CANCER 10
3.2 PRECLINICAL SCIENCE 11
3.3 CLINICAL DATA WITH FULVESTRANT 15
3.4 RATIONALE FOR COMBINATION OF ALISERTIB AND FULVESTRANT 16
3.5 RATIONALE TO ALLOW WOMEN WITH ER-NEGATIVE DISEASE TO PARTICIPATE 18
3.6 PHASE I TRIAL OF ALISERTIB AND FULVESTRANT 19
3.7 CIRCULATING TUMOR CELLS 19
3.8 STUDY SAFETY ASSESSMENT 20

4. PARTICIPANT SELECTION 22

4.1 PRE-REGISTRATION ELIGIBILITY CRITERIA 22
4.2 REGISTRATION ELIGIBILITY CRITERIA 24
4.3 REGISTRATION EXCLUSION CRITERIA 25
4.4 INCLUSION OF UNDERREPRESENTED POPULATIONS 26

5. REGISTRATION/RANDOMIZATION PROCEDURES 27

5.1 PRE-REGISTRATION (STEP 0) 27
5.2 REGISTRATION PROCEDURES 28
5.3 RANDOMIZATION PROCEDURES 30
5.4 OPTIONAL CROSSOVER PHASE 30

6. TREATMENT PLAN 31

6.1 ARM 1 (ALISERTIB MONOTHERAPY) 31
6.2 ARM 2 (ALISERTIB WITH FULVESTRANT) 32
6.3 CONCOMITANT TREATMENT AND SUPPORTIVE CARE GUIDELINES 33
6.4 TREATMENT/FOLLOW-UP DECISION AT EVALUATION OF PATIENT 34

7. EXPECTED TOXICITIES AND DOSING DELAYS/DOSE MODIFICATIONS 36

7.1 DOSE MODIFICATION BASED ON ADVERSE EVENTS 36
7.2 DOSE MODIFICATIONS – PRIOR TO EACH CYCLE OF TREATMENT 36
7.3 DOSE MODIFICATIONS – CYCLE 1 DAY 15 41

8. DRUG FORMULATION/STORAGE/SUPPLY 41

8.1 ALISERTIB (MLN8237, ML00653668) 41
8.2 FULVESTRANT (FASLODEX®) 45

9. CORRELATIVE/SPECIAL STUDIES 48

9.1 BODY FLUID BIOSPECIMENS ... 48
9.2 PATHOLOGY CONSIDERATIONS/TISSUE BIOSPECIMENS ... 51

10. SPECIMEN BANKING ... 54

11. STUDY CALENDAR ... 55

12. MEASUREMENT OF EFFECT ... 60

12.1 DEFINITIONS ... 60
12.2 DISEASE PARAMETERS ... 60
12.3 RESPONSE CRITERIA ... 62

13. ADVERSE EVENT REPORTING REQUIREMENTS ... 66

13.1 GENERAL ... 67
13.2 DEFINITIONS ... 67
13.3 REPORTING PROCEDURES ... 69
13.4 OTHER REQUIRED REPORTING ... 73
13.5 REQUIRED ROUTINE REPORTING ... 76

14. DATA AND SAFETY MONITORING ... 78

14.1 DATA MANAGEMENT AND REPORTING ... 78
14.2 ALL PAPER FORMS SHOULD BE SENT TO ... 80
14.3 LABELING ... 80
14.4 INCOMPLETE MATERIALS ... 80
14.5 OVERDUE LISTS ... 80
14.6 CORRECTIONS FORMS ... 80
14.7 EXTERNAL MONITORING ... 80

15. REGULATORY CONSIDERATIONS ... 81

15.1 PROTOCOL REVIEW AND AMENDMENTS ... 81
15.2 INFORMED CONSENT ... 81
15.3 ETHICS AND GCP ... 81

16. MULTI-CENTER GUIDELINES ... 82

16.1 STUDY DOCUMENTATION ... 82
16.2 RECORDS RETENTION ... 82
16.3 PUBLICATION ... 82

17. STATISTICAL CONSIDERATIONS AND METHODOLOGY ... 82

17.1 ASSESSING THE IMPACT OF ADDING FULVESTRANT TO ALISERTIB ... 82
17.2 SAMPLE SIZE/ ACCRUAL RATE ... 83
17.3 STRATIFICATION FACTORS ... 84
17.4 ANALYSIS OF SECONDARY ENDPOINTS ... 85
17.5 MONITORING ... 86
17.6 INCLUSION OF WOMEN AND MINORITIES ... 87

18. BUDGET ... 87

REFERENCES ... 88

APPENDICES ... 91

APPENDIX I – ECOG PERFORMANCE STATUS AND KARNOFSKY PERFORMANCE STATUS ... 91

APPENDIX II – NYHA HEART FAILURE CLASSIFICATION ... 92

APPENDIX III – MEDICATION DIARY ... 93

APPENDIX IV – PROTON PUMP INHIBITOR (PPI) AND H2 ANTAGONIST 95

SCHEMA

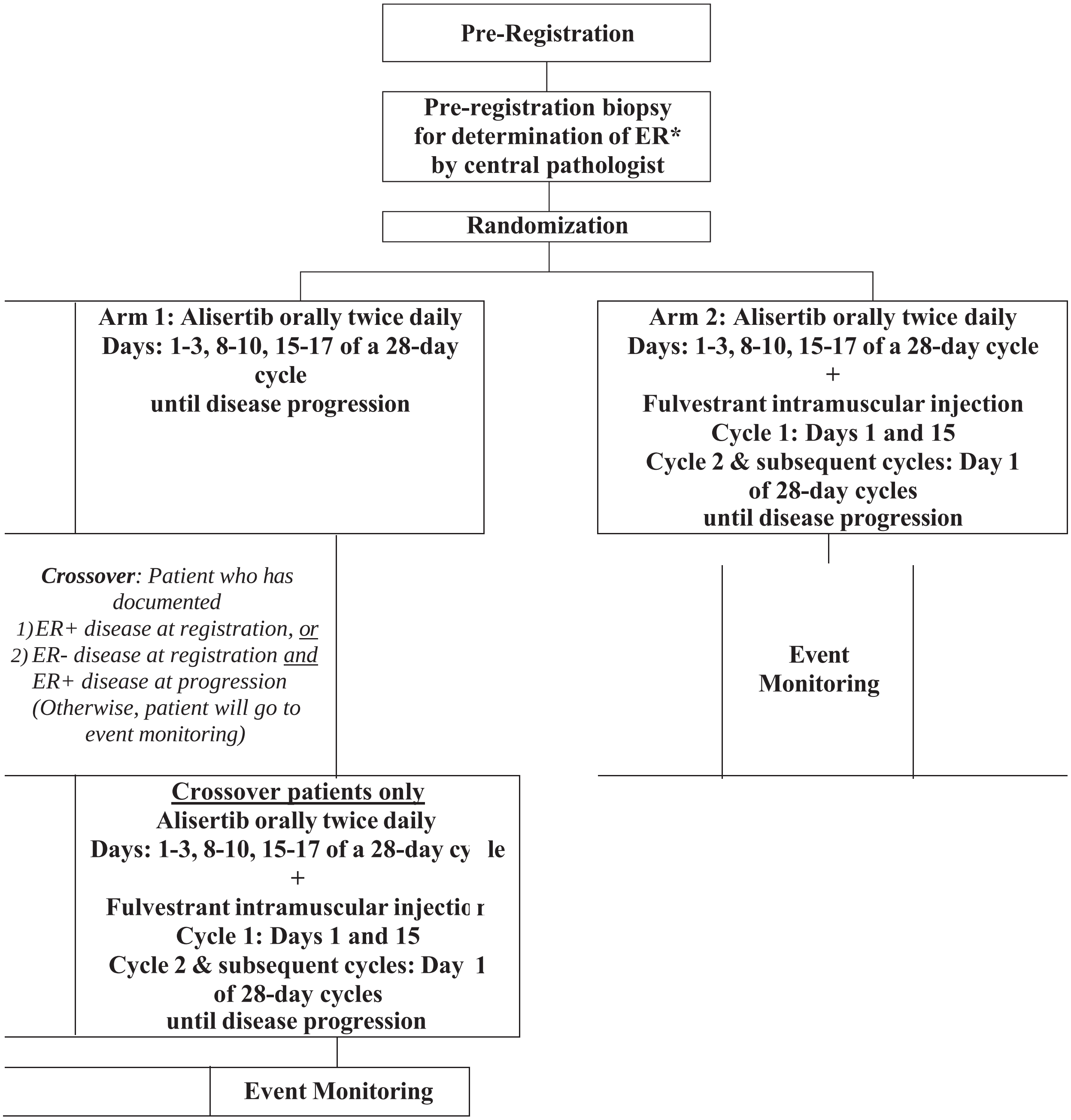

***Standard ER testing for ERα**

NOTE: If insufficient or no tissue was obtained by the pre-registration biopsy, an archival tissue specimen must be submitted for Central Laboratory ER determination prior to registration. Archived metastatic specimen preferred.

1. STUDY DESIGN/SUMMARY

Patients with ER+ metastatic breast cancer are typically treated with multiple lines of endocrine therapy, and tumor response rates to serial agents after first-line therapy tend to decrease. This coincides with ERα down-regulation, aggressive tumor behavior, and poor clinical outcomes. Novel therapies to overcome endocrine resistance are needed. Currently, there are no drugs either FDA-approved or in development to reverse endocrine resistance that results from down-regulation of the ERα.

Our preclinical data suggests that in ER+ breast cancer, Aurora A Kinase (AAK) is associated with the development of epithelial to mesenchymal transition (EMT) reprogramming and the expansion of breast tumor initiating cells that are $ER\alpha^{low/-}$ and resistant to endocrine therapy. We postulate that this is a mechanism by which ER down-regulation occurs. Thus targeted treatment with alisertib, an AAK inhibitor, is projected to reverse EMT and restore tumor ERα expression and sensitivity to endocrine therapy.

A randomized phase II trial to evaluate alisertib alone and in combination with fulvestrant is proposed. Based on our preclinical data, we project that the patients most likely to derive benefit from treatment with alisertib will be those that are resistant to endocrine therapy, so this will be our target population. Given the striking clinical activity of single agent alisertib observed in the cohort of heavily pretreated, ER+ metastatic breast cancer patients (n=26, CBR 88% and PFS 7.9 months), additional exploration of single agent activity in this patient population is warranted and justified. Based on our preclinical data, we project that the combination of alisertib and fulvestrant will result in superior clinical outcomes compared to alisertib alone. For this reason a head-to-head comparison of these two treatment strategies was selected.

Patients with a history of primary ER+ breast cancer or de novo metastatic ER+ breast cancer will be eligible. They will undergo a registration biopsy for determination of ER and HER2 status. Patients with HER2 positive disease will be ineligible. Based on historical literature, we predict that 10% of patients enrolling with a history of primary ER+ disease will have ER negative metastatic disease. These patients have a worse prognosis compared with those who retain ER+ expression. Given the preclinical data in vitro and in vivo data that supports that alisertib can restore ER+ expression and endocrine sensitivity, the investigators will offer enrollment to these patients to test this working hypothesis.

Patients will be randomized 1:1 to alisertib alone or in combination with fulvestrant. They will be stratified by (1) primary (de novo) or secondary (acquired) endocrine resistance and (2) level of ER expression (<10% or ≥10%).

Patients will remain on therapy until progression or intolerable toxicity or physician discretion. At the time of progression those on alisertib monotherapy may have the option to cross-over to the combination therapy. Those with baseline ER expression ≥10% can cross-over. Those with ER negative or <10% expression on the registration biopsy will only be allowed to cross-over if the ER status is clinically re-evaluated on the post-progression research biopsy. In that scenario only those that have disease ER ≥10% will be allowed to cross-over.

A rich panel of correlative studies will be included in the study design. Tumor biopsy and blood draw for circulating tumor cell assessment will be obtained at baseline, after completion of Cycle 1 treatment, and at the time of progression. In addition to this, in the Mayo Clinic cohort

of patients, research core biopsies will be utilized to generate patient-derived xenografts as a means to assess mechanisms of alisertib resistance.

2. OBJECTIVES

2.1 Primary Objective

To assess the impact on objective tumor response rate (using RECIST criteria) with the addition of fulvestrant to alisertib in women with endocrine resistant, advanced, estrogen receptor positive breast cancer.

2.2 Secondary Objectives

2.2.1 To evaluate the safety profile of each treatment regimen

2.2.2 To assess the impact on median progression-free survival with the addition of fulvestrant to alisertib

2.2.3 To obtain estimated tumor response rate and the median progression-free survival time during alisertib and fulvestrant treatment in the cohort of patients who progress during alisertib monotherapy, and crossover to receive the combination of alisertib and fulvestrant.

2.3 Correlative Research

2.3.1 To assess the changes in Aurora A kinase, SMAD5 and SOX2 expression and phosphorylation in tumor tissue after first cycle of assigned treatment

2.3.2 To assess the changes in ER expression and function in tumor tissue after the first cycle of assigned treatment

2.4 Exploratory Objectives

2.4.1 To generate patient derived xenografts (PDX) from tumors collected at baseline and progression of disease (PD) in order to identify mechanisms associated with both de novo and acquired alisertib resistance.
NOTE: **Xenografting will only be done for patients who enroll at Mayo Clinic in Rochester, Minnesota.**

2.4.2 After the first cycle of treatment, to assess changes in Aurora A kinase, p~SOX2 and ER expression on circulating tumor cells (CTCs), and to assess concordance between change in expression with tumor tissue and CTCs.

3. BACKGROUND

3.1 Scope of the Problem of Hormone Refractory Advanced Breast Cancer

Despite the clinical benefit of hormonal treatment in Estrogen Receptor positive (ER+) breast cancer, de novo and acquired resistance to therapies remains a significant clinical problem[1, 2]. Patients with metastatic ER+ breast cancer are typically treated with multiple lines of endocrine therapy, and tumor response rates to serial agents after first-line therapy tend to decrease[3]. This coincides with ERα down-regulation and aggressive tumor behavior and poor clinical outcomes[4,5]. Novel therapies to overcome endocrine resistance are needed.

The development of endocrine resistance is usually associated with aggressive tumor behavior and the development of distant metastases. One of the major barriers to eradicating metastatic cancer cells is overcoming their ability to self-renew and become resistant to conventional therapies.

The discovery that breast carcinomas contain a sub-population of cells harboring stem-like properties has generated excitement because these breast tumor initiating cells (BTICs) may represent a source of therapeutic failure.[6] Cancer cells that undergo the epithelial to mesenchymal transition (EMT) programming acquire a basal $CD44^{+}/CD24^{low/-}$ cancer stem-like phenotype with increased capacity for self-renewal, drug resistance, tumor initiation and metastases[7, 8]. Importantly, luminal breast cancer cells that undergo EMT display clonal expansion of BTICs that are $ER\alpha^{low/-}$ thus resulting in the generation of a "luminobasal" phenotype that is ultimately associated with the development of endocrine resistance and high risk of tumor recurrence[9, 10]. Aberrant activation of HER-2/MAPK and TGFβ/SMAD oncogenic signaling induces EMT and plays an important role in the maintenance and clonal expansion of BTICs[11-14]. This phenomenon is well established, however, the molecular mechanisms underlying ERα down-regulation and endocrine resistance by means of activation of EMT in ER+ breast cancer remain elusive and the subject of intense ongoing investigation.

Translational studies have demonstrated that breast cancer tumors with a higher population of BTICs are associated with shorter metastases-free survival in lymph node-negative, ER+/HER2- breast cancer, but not in ER- or HER2+ subtypes[15]. Additional studies demonstrate that ER+ tumor specimens collected from women following treatment with the aromatase inhibitor, letrozole, demonstrate a significant increase in mesenchymal-associated genes, as well as the $CD44^{+}/CD24^{low/-}$ cancer cell population, in the residual tumor[16]. These results suggest that the population of $CD44^{+}/CD24^{low/-}$ BTICs, particularly those surviving standard treatment with anti-estrogens, are relevant targets for novel anti-neoplastic therapeutics.

3.2 Preclinical Science

3.2.1 In Vitro and In Vivo Studies

Preclinical studies conducted by Dr. D'Assoro's research team[17] at Mayo Clinic demonstrate that constitutive expression of MAPK in MCF-7 cells (vMCF-7^{Raf1}) is associated with over-expression of the mitotic kinase Aurora A and the development of EMT and an invasive $CD44^{+}/CD24^{low/-}$ stem cell-like phenotype. Cancer cells undergoing EMT have increased motility and invasive behavior. They also are characterized by over-expression of proteins promoting EMT and stemness (SMAD5 and SOX2), as well as down-regulation of tumor suppressors involved in epithelial cell adhesion (PCDH8), inhibition of Aurora-A kinase activity (CHFR), and suppression of metastasis (TIMP2) and angiogenesis (THBS1) (Figure 1A).

In vivo studies demonstrate that vMCF-7^{Raf1} tumor xenografts display a loss of progesterone receptor (PR) and increased HER2 expression, higher tumor grade and development of spontaneous lung metastases (Figure 1C). These findings collectively reveal a novel, non-mitotic mechanism for the development of distant metastases and tumor survival by Aurora A kinase in ER+ breast cancer cells.

FIGURE 1

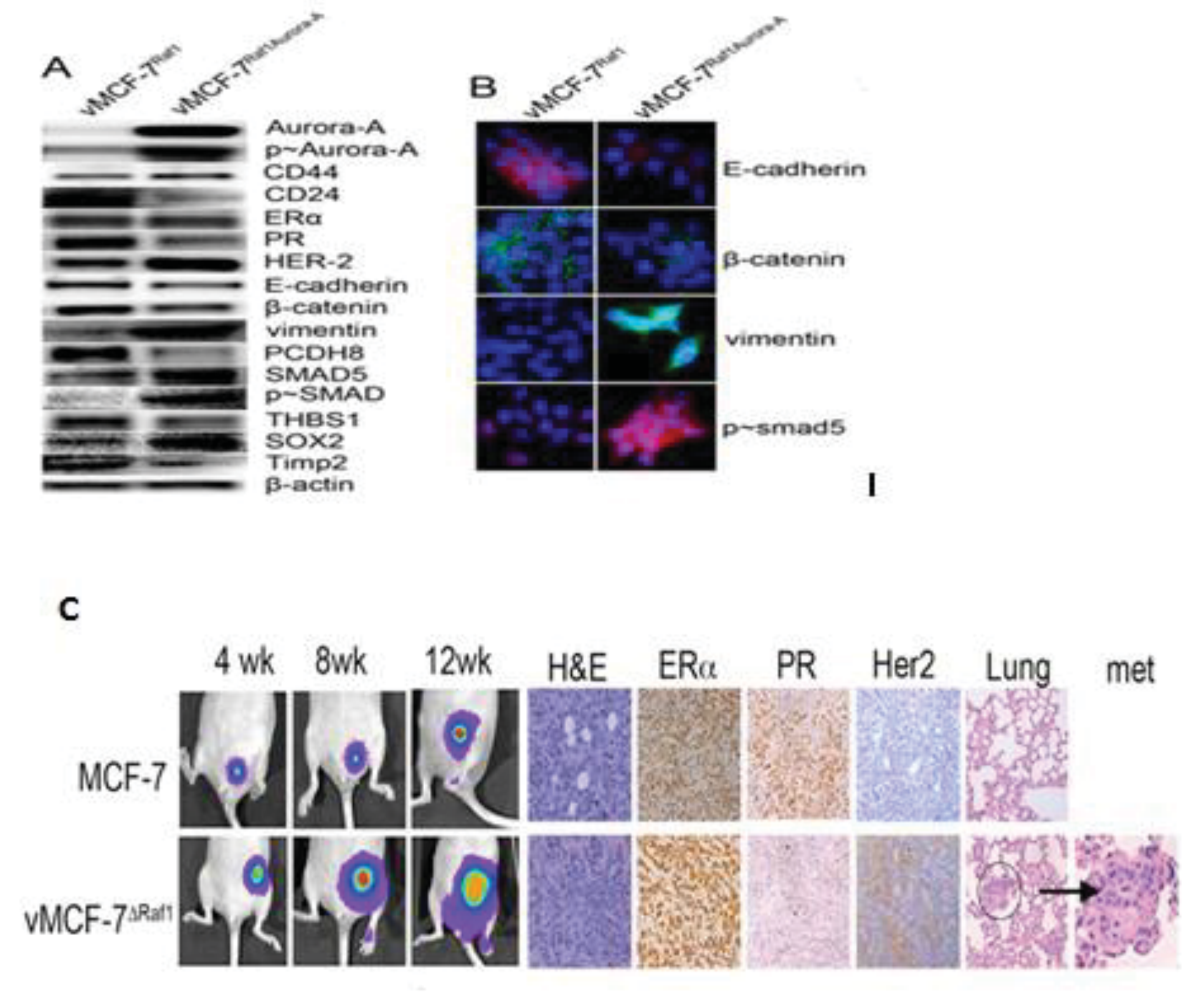

Aurora-A Over-Expression Induces the Development and Maintenance of $CD44^{+}/CD24^{low/-}$ Cancer Cells: (A) Immunoblotting analysis of MCF-7 cells over-expressing Aurora-A. (B) Immunofluorescence analysis of MCF-7 cells over-expressing Aurora-A. (C) In the raf-1 overexpressing tumor xenografts, tumors displayed retention of ER+, however, there is loss of PR and increased HER2 expression, higher tumor grade, and an association with spontaneous lung metastases.

The raf-1 overexpressing xenografts contain heterogeneity for the CD24 epithelial marker (Figure 2A-B). The $CD24^{low/-}$ cell sub-population displays overexpression and phosphorylation of Aurora A and is functionally linked to the mesenchymal phenotype; but importantly, these cells demonstrate a loss of ER expression, the primary target for endocrine therapy (Figure 2C).

Targeted treatment of $CD44^{+}/CD24^{low/-}$ breast cancer cells with alisertib, an aurora-A kinase inhibitor, suppressed EMT and restored the CD24+ epithelial

phenotype (Figure 2D-E). These tumors were furthermore characterized by loss of HER-2 expression and nuclear SMAD5 phosphorylation, as well as restoration of ER expression. To validate in vivo the therapeutic effect of molecular inhibition of Aurora-A activity in the suppression of metastases, vectors were engineered to express shRNAs to knockdown Aurora-A in the vMCF-7^{Raf1} xenograft model (Figure 2F)[18]. Inhibition of Aurora-A kinase induced tumor regression and reduced the number of lung metastases.

FIGURE 2

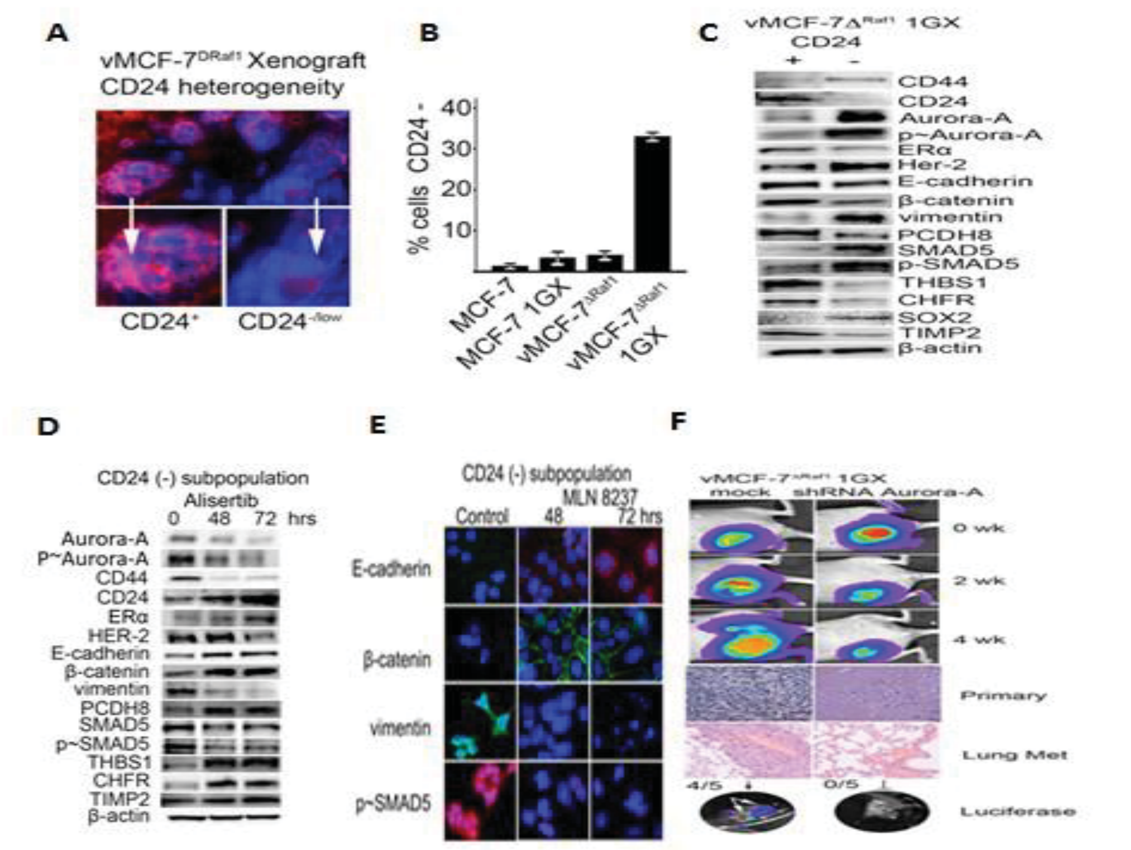

Inhibition of Aurora-A kinase activity in CD24$^{low/-}$ cells suppresses EMT, tumor self-renewal and breast cancer metastases: (A) Immunofluorescence analysis of CD24$^{low/-}$, raf-1 overexpressing MCF-7 breast cancer cells (B) Measurement of CD24$^{low/-}$ cells by flow cytometry, including cells obtained from raf-1 overexpressing MCF-7 tumor xenografts re-suspended in culture (first generation, 1GX) (C) Immunoblotting analysis of the CD24 positive and negative sub-populations of cells (D) Immunoblotting analysis of CD24$^{low/-}$ cells treated with the Aurora A kinase inhibitor, alisertib (MLN 8237) (E) Immunoflurorescence of CD24$^{low/-}$ cells treated with the Aurora A kinase inhibitor, alisertib (MLN 8237) (F) Tumor xenografts treated with empty shRNA and shRNA Aurora-A vectors.

These in vitro and in vivo pre-clinical results demonstrate that pharmacologic inhibition of Aurora-A kinase activity by alisertib offers a novel therapeutic strategy to selectively target CD44$^+$/CD24$^{low/-}$ invasive cancer cells, suppress the

early stages of EMT, and consequent metastatic dissemination to distant organs. These results have recently been suggested by others[19].

3.2.2 Evaluation of Aurora A in human breast cancer biospecimens

In translational studies by Dr. D'Assoro's team (unpublished data), Aurora A kinase, p~SMAD5, p~SOX2 expression was evaluated in a cohort of 100 tissue biospecimens obtained from women with early stage breast cancer treated with optimal locoregional therapy and 5 years of adjuvant endocrine therapy. The "bad outcome" group is defined as patients enduring early breast cancer relapse or death within 5 years of diagnosis, and the "good outcome" group is defined as those with disease-free survival of at least 7 years. Aurora A kinase is measured by immunohistochemistry on a tissue microarray, and the extent and intensity of staining is scored. Tumor samples exhibiting higher levels of Aurora A expression were associated with increased p~SMAD5 and p~SOX2 expression as well as poor clinical outcomes (Figure 3).

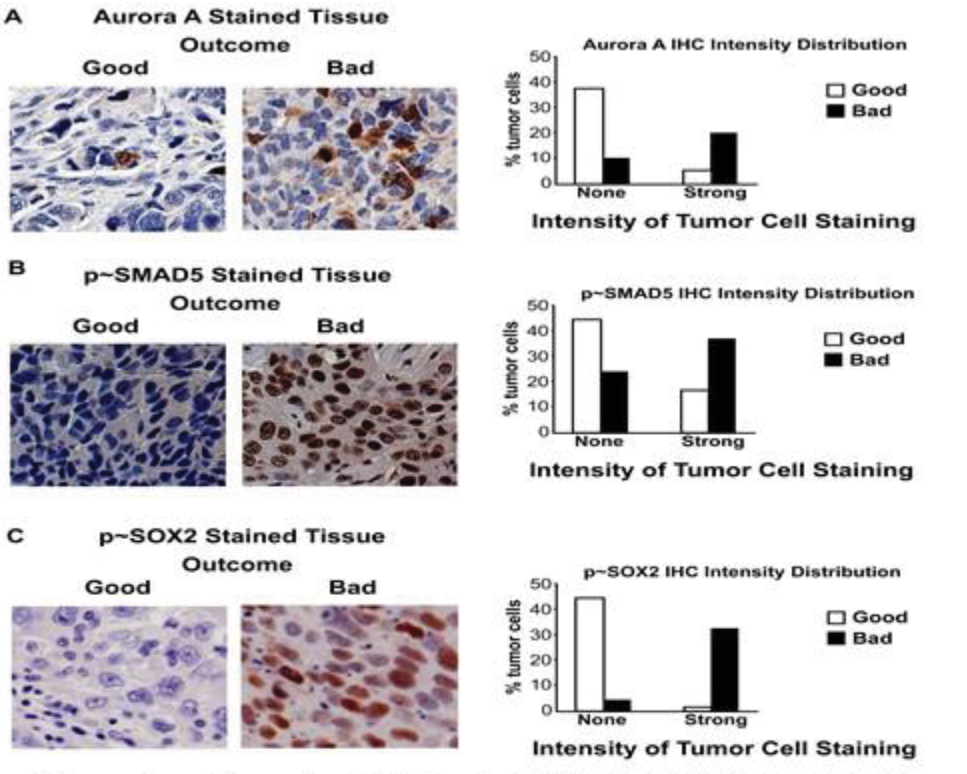

Figure3. Expression of Aurora-A, p~SMAD5 and p~SOX2 in Selected Cohorts of ER+ Human Breast Tumors: (A) Aurora-A staining and Graph showing Aurora-A intensity in breast tumors with bad and good outcome. (B) p~SMAD5 staining and Graph showing P~SMAD5 intensity in breast tumors with bad and good outcome. (C) p~SOX2 staining and Graph showing P~SOX2 intensity in breast tumors with bad and good outcome.

These findings are further supported by others[15]. In this study the tumor tissue expression of Aurora A was evaluated in three (3) separate cohorts of women with early stage breast cancer treated without adjuvant therapy (n=766). Their work demonstrated an association of Aurora A with shorter metastases-free survival. The correlation was statistically significant in ER+/HER2- breast cancer, but not in the biologically more aggressive ER-/HER2- or HER2+ molecular subtypes. Further support and validation of our preclinical and translational work is provided by Thrane et al[19]. Thus, patients with ER+ breast cancer and high levels of tumor expression of Aurora A kinase may benefit from targeted treatment with alisertib; in fact, it is feasible that patients with initial ER+ primary disease that is $ER^{low/-}$ at metastatic relapse or progression may also benefit. These women are known to have particularly inferior clinical outcomes compared with women who retain ER+ expression at relapse.[20]

3.2.2.1 Early Phase Clinical Trials of Alisertib

To date, alisertib has been extensively evaluated in up to 1100 patients with hematological malignancies and to a lesser extent in those with solid tumors, including breast cancer[21, 22]. The maximum tolerated dose has been found to be 50 mg twice daily on Days 1-7 of a 21-day cycle, and it has been used without untoward toxicities in ongoing phase II clinical trials[22]. An alternative 28-day regimen with alisertib given Days 1-3, 8-10 and 15-17 was studied in combination with paclitaxel in xenograft breast and ovarian cancer models, and it is associated with equivalent drug levels, decreased incidence of dose limiting neutropenia with negligible compromise to efficacy.[23] Results of a phase II study of single agent alisertib (21-day schedule) in relapsed/refractory metastatic breast cancer (from a cohort of all solid tumors) were presented at the San Antonio Breast Cancer Symposium, December 2013 (Abstract PD5-5) and subsequently published in 2015[24]. Of 26 patients in the heavily-pretreated, hormone receptor positive cohort, 23% had an objective response (complete or partial response), and 31% achieved disease stabilization (at least 6 months without disease progression) for a clinical benefit rate of 54%. The median PFS was 7.9 months. Therapy was generally well tolerated with the most commonly Grade 2 or less adverse events being neutropenia, fatigue, and diarrhea. Given the safety and efficacy profile of alisertib in ER+/HER2- metastatic breast cancer, further evaluation to validate these findings is warranted and justifies the inclusion of an alisertib monotherapy arm in this trial.

3.3 Clinical data with fulvestrant

Fulvestrant is an FDA-approved agent for the treatment of post-menopausal ER+ metastatic breast cancer. It is a pure ER antagonist given by intramuscular injection in a 28-day cycle. It is exceptionally well-tolerated with the most frequent side effects being mild hot flashes and arthralgias. The CONFIRM trial[25] is a published phase III randomized trial of fulvestrant (500 mg Day 1 and 15 of cycle 1 and 500 mg Day 1 of subsequent cycles) and fulvestrant (250 mg Day 1

each cycle) as first line treatment for advanced ER+ breast cancer. The trial population included patients who relapsed on adjuvant endocrine treatment or within one year of completing adjuvant endocrine therapy; for those with relapse >1year after completion of adjuvant therapy or de novo stage IV disease, progression on first line anti-estrogen therapy was required. Among 240 patients with measurable disease receiving the 500mg dose, the ORR was 13.8%. Fulvestrant can induce anti-tumor response in disease with prior hormonal treatment exposure; however, the ORR is less than desirable and in part reflects the scope of the problem of resistance to hormonal therapy.

3.4 Rationale for Combination of Alisertib and Fulvestrant

The rationale to support this phase II clinical trial evaluating alisertib in combination with fulvestrant is based on the in vitro studies from Dr. D'Assoro's lab. These experiments demonstrate decreased cell proliferation by single agent alisertib in MCF-7 ER+ breast cancer cells (Figure 3). The effect on cell proliferation is further enhanced when alisertib is combined with fulvestrant when compared to either agent alone. These preliminary results have been confirmed in tamoxifen-resistant vMCF-7^{raf-1} 1GX cells whereby combination of fulvestrant with alisertib induced a stronger inhibition of cell proliferation[18] (Figure 4).

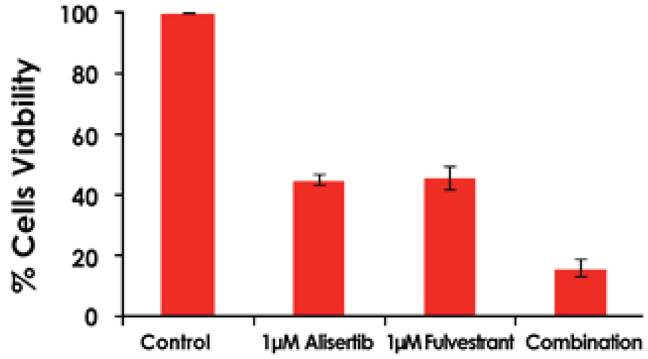

Figure 3. MCF-7 ERα+ Breast Cancer Cells treated with Alisertib and/or Fulvestrant: Cell viability was assessed by MTT assay following 7 day treatment. Graph showing the percentage of viable cells as compared to control from three independent experiments (+/- s.d).

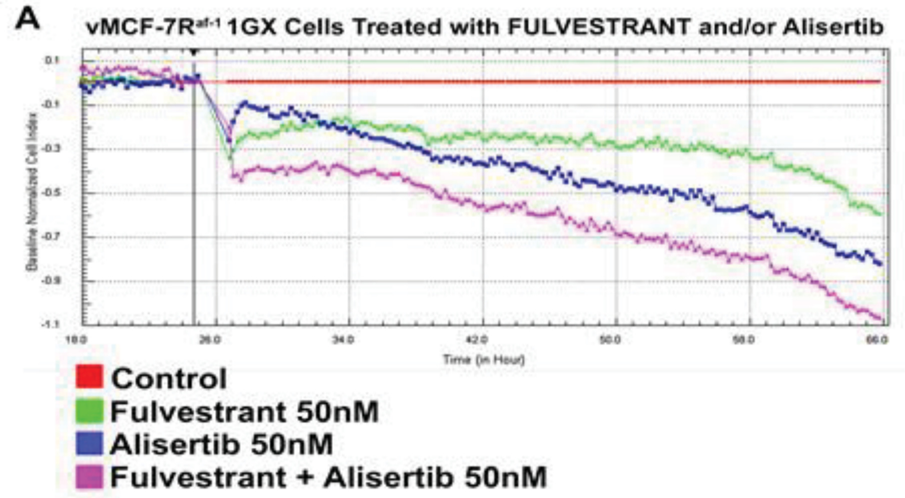

Figure 4: Restoration of Endocrine Sensitivity in vMCF-7$^{\Delta Raf-1}$ 1GX Cells

Finally, studies in Dr. D'Assoro's lab have demonstrated that alisertib restored sensitivity to fulvestrant in variant MCF-7/LCC9 *ex-vivo* cancer cells that display intrinsic resistance to tamoxifen and fulvestrant in tumor xenografts (Figure 5). Because treatment with fulvestrant induces activation of apoptosis in endocrine sensitive breast cancer cells, a real-time apoptosis assay was performed to establish the extent to which restoration of fulvestrant sensitivity was linked to activation of apoptosis in alisertib-treated MCF-7/LLC9 cells. Notably, while MCF-7/LCC9 cells treated with fulvestrant showed nominal levels of apoptosis, combination of fulvestrant with alisertib significantly increased the percentage of apoptotic cells (Figure 6). Importantly, fulvestrant resistance in MCF-7/LCC9 cells was linked to increased expression of AAK and ERα down-regulation when compared to endocrine sensitive MCF-7 parental cells (Figure 7), indicating that intrinsic endocrine resistance is associated to aberrant AAK activity leading to loss of dependence on ERα signaling.

Collectively, these preliminary findings support the rationale for the proposed combination therapy and the underlying hypothesis that in women with ER+ metastatic breast cancer that is resistant to fulvestrant, alisertib will target the subpopulation of $CD44^{+}/CD24^{low/-}$ BTICs that over-express Aurora-A kinase and restore ERα expression, functional activity, and thereby sensitivity to fulvestrant.

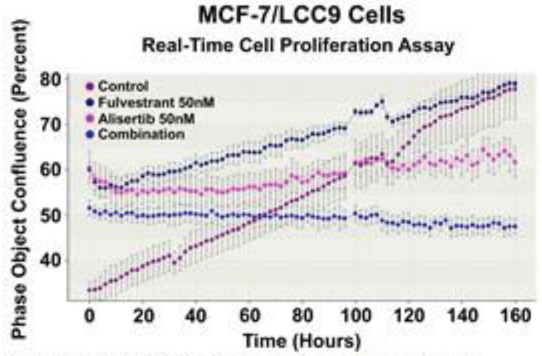

Figure 5. AAK Targeting Restores Fulvestrant Sensitivity: *In Vitro* Real-Time Cell Proliferation Assay showing restoration of endocrine sensitivity when 50nM Fulvestrant was combined with 50nM Alisertib in MCF-7/LCC9 cells. Experiments were performed in triplicate (±s.d.).

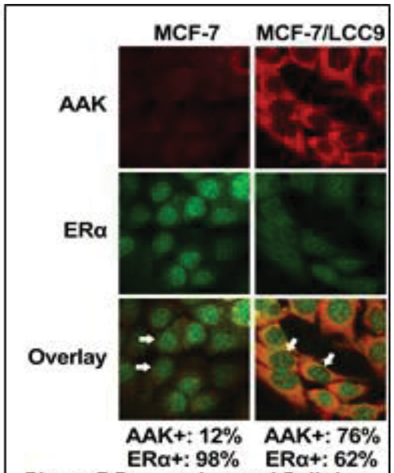

Figure 7. Expression and Cellular Localization of AAK and ERα in MCF-7 and MCF-7/LCC9 Variant cells: Immunofluorescence assay showing increased cytoplasmic expression of AAK (Red) and ERα (Green) nuclear down-regulation in MCF-7/LCC9 variant cells.

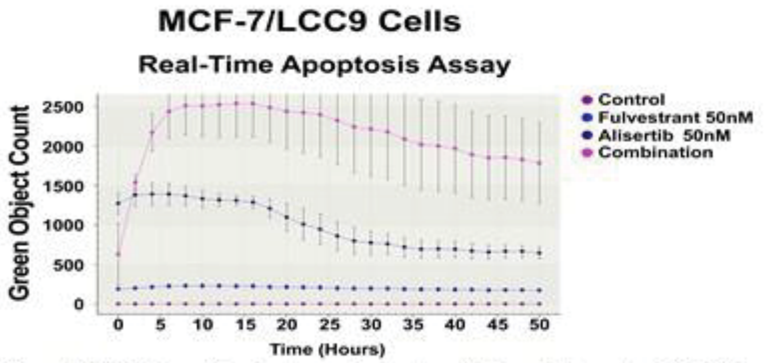

Figure 6. AAK Targeting Restores Fulvestrant-Induced Apoptosis in MCF-7/LCC9 cells: *In Vitro* Real-Time Apoptosis Assay showing stronger activation of apoptosis when 50nM Fulvestrant is combined with 50nM Alisertib in MCF-7/LCC9 cells. Apoptotic cells were stained in green with YOYO-1 Iodide and quantified using the *Cell Player System*. Experiments were performed in triplicate (± s.d.).

3.5 Rationale to Allow Women with ER-negative Disease to Participate

It is feasible that women with a history of primary, operable breast cancer that is ER+ who endure metastatic relapse that is ER <10% or ER negative, may derive benefit from alisertib. This is known to occur in up to 20% of patients. The hazard of death in women with ER+ primary breast cancer whose recurrence is ER <10% or ER negative is 1.48 times (95% CI, 1.08 to 2.05) that of woman who retain ER+ at metastatic relapse.[20] These tumors may be heavily comprised of the Aurora-A kinase over-expressing $CD44^{+}/CD24^{low/-}$ $ER\alpha^{low/-}$ BTICs that alisertib targets. In fact, alisertib may allow for reversion of the luminobasal phenotype and restoration of ERα expression, functional activity, and thus sensitivity to hormonal treatment.

3.6 Phase I Trial of Alisertib and Fulvestrant

A phase I trial to evaluate the safety of the combination of alisertib and fulvestrant is active, but has completed enrollment at the Mayo Clinic Rochester (MC1231; NCT02219789). Post-menopausal women with advanced hormone receptor-positive breast cancer were eligible, including those who have previously experienced disease progression on fulvestrant. This trial examined the pulse dose 28-day schedule and dosing of alisertib in conjunction with standard dose and 28-day schedule fulvestrant (500 mg IM on Days 1 and 15 of Cycle 1, then day 1 of all subsequent cycles). A standard 3+3 study design approach was utilized to evaluate 2 dose levels of alisertib (40 and 50mg) given oral twice daily on days 1-3, 8-10, 15-17 of a 28-day cycle. The primary endpoint was safety and tolerability assessment of the combination therapy. The RP2D was determined for the purpose of this phase II trial.

Ten patients enrolled during the period September 2014 - April 2015. One was found to be ineligible and was not included in the analysis. The median patient age was 59 (range 48, 73). Prior endocrine therapies included an aromatase inhibitor (9, 100%), fulvestrant (6, 67%), and everolimus+exemestane (5, 56%) amongst others. Eight (89%) had prior chemotherapy. There were no severe (Grade 3+) toxicities reported during cycle 1 at either dose level, thus the MTD was not reached. The recommended phase II dose of alisertib in combination with fulvestrant is 50mg. The cycle 1 grade 1/2 adverse events regardless of attribution were fatigue (6, 67%), neutropenia (5, 56%), anemia (5, 56%), diarrhea (3, 33%), nausea (3, 33%), and mucositis (1, 11%). As of December 30, 2015, a median of 10 cycles of therapy have been administered (range 2, 16+), and the 6-month clinical benefit rate was 77.8% (95% CI: 40.0-87.2%). Five patients remain on treatment with stable disease.

3.7 Circulating Tumor Cells

Because the onset of metastases results from the successful dissemination of cancer cells from the primary tumor to distant organs, the relative abundance of CTCs in the peripheral blood has strong correlation with tumor recurrence and distant metastases in both early stage and advanced breast tumors[27-29]. The development of CTCs as a “liquid biopsy” by which to perform ongoing tumor assessments in relation to treatment exposure is possible with recent technologic advances that allow for the capture of CTCs with epithelial, EMT-like, and/or stem cell like phenotypes, and for molecular characterization by immunocytochemistry, fluorescence in situ hybridization, RT-PCR, and next generation sequencing. Mayo Clinic Rochester has access to several platforms for CTC capture and analysis with the goal of studying the most clinically relevant population of CTCs for a given translational research question. Preliminary studies at Mayo demonstrate that p~SOX2 nuclear expression in CTCs from a patient with MBC can be observed (Figure 8).

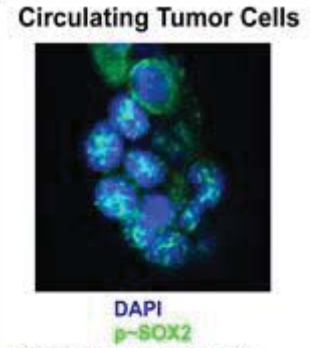

Figure 8. Expression of p~SOX2 in CTCs: p~SOX2 nuclear expression in CTCs isolated from a MBC patient. Nuclear phosphorylation of SOX2 was detected by Immunofluorescence employing the Zeiss Confocal Fluorescent Microscope.

3.8 Study Safety Assessment

The safety stopping boundary was crossed after 12 patients had received at least one cycle of treatment, with 5 of these 12 patients developing a Grade 4 toxicity or dying on treatment. The study was temporarily closed to accrual on 02Apr2018 for the protocol chair and the study biostatistician to review all the AE data.

The findings are summarized below:

Alisertib monotherapy arm:

Three of the 5 patients randomized to this treatment arm had adverse events associated with the safety stopping rule: two deaths on study and one patient with Grade 4 toxicities. The two patients who died on study were symptomatic of their disease at time of enrollment.

- The first death was due to Grade 5 hemolysis (thrombotic microangiopathy) felt to be likely due to disease progression; however, it was attributed by the treating physician as ‘possibly’ related to alisertib.
- The second death was a Grade 5 acute respiratory failure as initial manifestation of an acute coronary syndrome, attributed as ‘possibly’ related to alisertib.
- The third event was a patient who developed Grade 4 toxicities during Cycle 1, including neutropenia on Day 15, but also a Grade 4 cerebral edema due to newly diagnosed brain metastases. The latter event was not attributed to the drug, and the patient came off treatment due to disease progression.
- Of note: Two additional patients have been treated in Arm 1, having received 4-5 cycles of therapy without any severe (grade ≥4) toxicities.

In review of the investigator’s brochure, in over 1300 patients treated with alisertib, hemolysis had not been previously reported prior to this event, and there was only a single report of acute respiratory failure and acute coronary syndrome each. It remains unknown if alisertib contributed to any of these toxicities.

Alisertib in combination with fulvestrant arm:

Two of the 7 patients randomized to this treatment arm had adverse events associated with the safety stopping rule: one death on treatment and one patient with Grade 4 toxicities.

- The patient who died did so on Day 8 of Cycle 1. She was not symptomatic of her disease at enrollment; however, she went on to have declining performance and respiratory status during her 21-day wash out from chemotherapy prior to registration. She was subsequently hospitalized days after initiating treatment due to increasing lethargy and declining respiratory condition attributed to an aspiration pneumonia event. She died due to disease progression.
- The second event was a patient who was symptomatic of her hepatic metastasis and had evidence of pseudocirrhosis at enrollment. She received 4 cycles of therapy on study during which time she experienced several Grade 4 toxicities, including cytopenias, hyperbilirubinemia, as well as hyponatremia. The

hematologic toxicities were attributed to alisertib, but exacerbated by her underlying liver disease. She came off study due to disease progression.

- Of note: Five additional patients have been treated in Arm 2, having received 2-4 cycles of therapy without any severe (grade ≥4) toxicities.

Decision:

After some discussions, the study team has revised the protocol (Amendment #3) to tighten the eligibility criteria such that patients who are moderately to severely symptomatic of their disease (clinically or by laboratory analysis) or experiencing a decline in performance status would not be eligible to participate. An adverse event assessment will be performed and the laboratory evaluation will be repeated at the time of registration insuring washout from the most recent therapy to reduce the risk of having a patient with rapidly progressive disease participating.

4. PARTICIPANT SELECTION

4.1 Pre-registration Eligibility Criteria

4.1.1 Females age ≥18 years

4.1.2 Post-menopausal defined as

- Age ≥60 and amenorrhea >12 consecutive months
  OR
- Previous bilateral oophorectomy
  OR
- Age <60 and amenorrhea >12 consecutive months and documented follicle stimulating hormone (FSH) level within post-menopausal range according to institutional standard

NOTE: If FSH level not within post-menopausal range according to institutional standard, but there is other evidence of post-menopausal status, exceptions may be granted by the PI.

4.1.3 Histologic proof of metastatic or locally advanced, unresectable breast cancer

4.1.4 History of ER positive (+) (≥10% of cells positive on H&E), HER2 negative (−) breast cancer disease, either as a

(1) History of primary, operable ER+/HER2− invasive breast cancer

OR

(2) History of *de novo* metastatic breast cancer that is ER+/HER2−

Note: HER2− (negative) disease defined as one of the following:

- HER2 IHC expression of 0, 1+ and ISH non-amplified
- HER2 IHC expression of 0, 1+ and ISH not done
- HER2 IHC expression of 2+ and ISH non-amplified
- IHC not done and ISH non-amplified

Note: supporting documentation such as a pathology report from their primary diagnosis that indicates ER+/Her2- invasive breast cancer or a biopsy report of de novo metastatic breast cancer that is ER+/HER2− should be submitted through the RAVE database

4.1.5 Prior treatment

- No more than two prior chemotherapy regimens in the metastatic setting.
- Prior treatment with fulvestrant in the metastatic setting is required, except for patients with a history of ER-negative metastatic breast cancer
- Unlimited prior endocrine therapy regimens in the metastatic setting are allowed

- No prior treatment with an Aurora Kinase inhibitor (either an Aurora A or pan-Aurora kinase inhibitor)

4.1.6 Disease that is measurable where:
-A non-nodal lesion is considered measurable if its longest diameter can be accurately measured as ≥2.0 cm with chest x-ray, or as ≥1.0 cm with CT scan, CT component of a PET/CT, or MRI.
-A malignant lymph node is considered measurable if its short axis is ≥1.5 cm when assessed by CT scan (CT scan slice thickness recommended to be no greater than 5 mm).

Note: Tumor lesions in a previously irradiated area are not considered measurable disease.

Note: Disease that is measurable by physical examination only is not eligible.

4.1.7 No history of tumors involving spinal cord or heart.

4.1.8 Fully recovered from acute, reversible effects of prior therapy regardless of interval since last treatment
EXCEPTION: Neuropathies – if Grade 2 neuropathies have been stable for at least 3 months since completion of prior treatment, patient is eligible.

4.1.9 a ECOG Performance Status: 0 or 1(See Appendix I)

4.1.9b No requirement for constant administration of proton pump inhibitor, H2 antagonist, or pancreatic enzymes (see Appendix IV)

4.1.9c Willing to limit daily alcohol intake to the following: one 12-oz glass of beer, one 6-oz glass of wine, or one 1.5-oz portion of 80-proof alcohol.

4.1.9d No uncontrolled intercurrent illness including, but not limited to:
- ongoing or active infection
- symptomatic congestive heart failure
- unstable angina pectoris
- uncontrolled symptomatic cardiac arrhythmia
- uncontrolled hypertension (defined as blood pressure >160/90)

4.1.9e No history of uncontrolled sleep apnea syndrome and other conditions that could result in excessive daytime sleepiness, such as severe chronic obstructive pulmonary disease, or requirement for supplemental oxygen.

4.1.9f No other active second malignancy other than non-melanoma skin cancers and in situ cervical cancers within 5 years of registration.
NOTE: A second malignancy is not considered active if all treatment for that malignancy is completed and the patient has been disease-free for at least 5 years prior to registration

4.1.9g Ability to provide written informed consent.

4.1.9h Willing to return to enrolling institution for follow-up during the active treatment. Event monitoring following completion of therapy may occur outside the enrolling institution.

4.1.9i No history of myocardial infarction ≤6 months prior to pre-registration or New York Heart Association (NYHA) Class III or IV heart failure (see Appendix II), uncontrolled angina, severe uncontrolled ventricular arrhythmias, or electrocardiographic evidence of acute ischemia or active conduction system abnormalities

4.1.9j No prior allogeneic bone marrow or organ transplantation

4.1.9k No known clinical finding or suspicion of human immunodeficiency virus (HIV) infection, hepatitis B, or hepatitis C.

4.1.9l No co-morbid systemic illnesses or other severe concurrent disease which, in the judgment of the investigator, would make the patient inappropriate for entry into this study or interfere significantly with the proper assessment of safety and toxicity of the prescribed regimens.

4.1.9m Able to swallow oral medication

4.1.9n No known GI disease or GI procedures that could interfere with the oral absorption or tolerance of alisertib. Examples include, but are not limited to partial gastrectomy, history of small intestine surgery, and celiac disease.

4.1.9o No visceral crisis: Visceral crisis is moderate-to-severe organ dysfunction as assessed by symptoms and signs, laboratory studies, and rapid progression of disease.

4.1.9p Willing to undergo a biopsy of a metastatic site of breast disease for central laboratory determination of ER and correlative research purposes. NOTE: If insufficient or no tissue is obtained by the pre-registration biopsy, an archival tissue specimen (preferably from a metastatic site) must be available to submit for Central Laboratory ER determination prior to registration.

4.2 Registration Eligibility Criteria

4.2.1 ≤28 days post pre-registration

4.2.2 Central Laboratory ER determination completed.

4.2.3 The following laboratory values obtained ≤14 days prior to randomization:

- Absolute neutrophil count (ANC) ≥1500/mm^3
- Platelet count ≥100,000/mm^3
- Hemoglobin ≥9.0 g/dL
- Total bilirubin ≤ULN
  NOTE: If total bilirubin is out of range then direct bilirubin must be ≤ULN

- Alanine transaminase (ALT) ≤2.5 x ULN (≤5 x ULN for patients with liver involvement)
- Calculated creatinine clearance must be ≥45 ml/min using the Cockcroft-Gault formula below:

Cockcroft-Gault Equation:

Creatinine clearance for females = $\frac{(140 - \text{age})(\text{weight in kg})(0.85)}{(72)(\text{serum creatinine in mg/dL})}$

4.2.4 ECOG Performance Status: 0 or 1 (See Appendix I)

4.2.5 Life expectancy of ≥6 months.

4.2.6 Willing to provide blood and tissue for correlative research purposes.

4.3 Registration Exclusion Criteria

4.3.1 Any of the following therapies prior to registration:
- Chemotherapy ≤21 days
- Immunotherapy ≤21 days
- Biologic therapy (includes monoclonal antibodies) ≤21 days
- Hormonal therapy ≤14 days except fulvestrant ≤21 days
- Targeted therapies including CDK 4/6 inhibitors (e.g. palbociclib, abemaciclib, ribociclib, etc.) and mTOR inhibitors (e.g. everolimus) ≤21 days
- Other investigational agents ≤21 days
- Radiation therapy ≤14 days
- Minor surgical or interventional procedure ≤7 days
- Major surgical procedure ≤21 days

4.3.2 Administration of myeloid growth factors or platelet transfusion ≤14 days prior to registration.

4.3.3 Systemic infection requiring IV antibiotic therapy ≤14 days prior to registration.

4.3.4 Treatment with clinically significant enzyme inducers, such as the enzyme-inducing antiepileptic drugs phenytoin, carbamazepine or phenobarbital, or rifampin, rifabutin, rifapentine or St. John's wort ≤14 days prior to registration.

4.3.5 Require constant administration of proton pump inhibitor, H2 antagonist, or pancreatic enzymes (see Appendix IV).

4.3.6 Development of visceral crisis since pre-registration.
NOTE: Visceral crisis is moderate-to-severe organ dysfunction as assessed by symptoms and signs, laboratory studies, and rapid progression of disease.

4.3.7 History of brain metastases.

4.4 Inclusion of Underrepresented Populations

Individuals of all races and ethnic groups are eligible for this trial. There is no bias towards age or race in the clinical trial outlined. This trial is open to the accrual of women only.

5. REGISTRATION/RANDOMIZATION PROCEDURES

5.1 Pre-Registration (Step 0)

5.1.1 TBCRC Institutions other than Mayo Clinic

To pre-register a patient, fax (507-284-0885) a completed eligibility checklist to the Mayo Clinic Cancer Center (MCCC) Registration Office between 8 a.m. and 4:30 p.m. Central Time Monday through Friday.

5.1.2 Mayo Clinic Institutions

To register a patient, access the Mayo Clinic Cancer Center (MCCC) web page and enter the registration/randomization application. The registration/ randomization application is available 24 hours a day, 7 days a week. Back up and/or system support contact information is available on the website. If unable to access the website, call the MCCC Registration Office at (507) 284-2753 between the hours of 8 a.m. and 5:00 p.m. Central Time (Monday through Friday).

The instructions for the registration/randomization application are available on the MCCC web page (http://ccswww.mayo.edu/ccs/training) and detail the process for completing and confirming patient registration. Prior to initiation of protocol treatment, this process must be completed in its entirety and an MCCC subject ID number must be available as noted in the instructions. It is the responsibility of the individual and institution registering the patient to confirm the process has been successfully completed prior to release of the study agent. Patient registration via the registration/randomization application can be confirmed in any of the following ways:

- Contact the MCCC Registration Office (507) 284-2753. If the patient was fully registered, the MCCC Registration Office staff can access the information from the centralized database and confirm the registration.
- Refer to “Instructions for Remote Registration” in section “Finding/Displaying Information about A Registered Subject.”

5.1.3 Prior to accepting the pre-registration, the registration/randomization application will verify the following:

- IRB approval at the registering institution
- Patient pre-registration eligibility
- Existence of a signed consent form
- Existence of a signed authorization for use and disclosure of protected health information

5.1.4 Pre-registration tests/procedures (see Section 11.0) must be completed within the guidelines specified on the test schedule.

5.2 Registration Procedures

5.2.1 TBCRC Institutions other than Mayo Clinic

5.2.1.1 Registration

To register a patient, fax (507-284-0885) a completed eligibility checklist to the Mayo Clinic Cancer Center (MCCC) Registration Office between 8 a.m. and 4:30 p.m. Central Time Monday through Friday.

5.2.1.2 Correlative Research

A mandatory correlative research component requiring blood and tissue (see Section 9) is part of this study. The patient will be registered onto this component by the MCCC Registration Office.

5.2.2 Mayo Clinic Institutions

5.2.2.1 Registration

To register a patient, access the Mayo Clinic Cancer Center (MCCC) web page and enter the registration/randomization application. The registration/ randomization application is available 24 hours a day, 7 days a week. Back-up and/or system support contact information is available on the Web site. If unable to access the Web site, call the MCCC Registration Office at (507) 284-2753 between the hours of 8 a.m. and 5:00 p.m. Central Time (Monday through Friday).

The instructions for the registration/randomization application are available on the MCCC web page (http://ccswww.mayo.edu/training/) and detail the process for completing and confirming patient registration. Prior to initiation of protocol treatment, this process must be completed in its entirety and a MCCC subject ID number must be available as noted in the instructions. It is the responsibility of the individual and institution registering the patient to confirm the process has been successfully completed prior to release of the study agent. Patient registration via the registration/randomization application can be confirmed in any of the following ways:

- Contact the MCCC Registration Office (507) 284-2753. If the patient was fully registered, the MCCC Registration Office staff can access the information from the centralized database and confirm the registration.
- Refer to "Instructions for Remote Registration" in section "Finding/Displaying Information about A Registered Subject."

5.2.2.2 Correlative Research

A mandatory tissue and blood correlative research component is part of this study. The patient will be automatically registered onto this component (see Section 9*)*.

5.2.3 All Institutions

5.2.3.1 Documentation of IRB approval must be on file in the MCCC Registration Office before an investigator may register any patients.

In addition to submitting initial IRB approval documents, ongoing IRB approval documentation must be on file (no less than annually) at the Registration Office (fax: 507-284-0885). If the necessary documentation is not submitted in advance of attempting patient registration, the registration will not be accepted and the patient may not be enrolled in the protocol until the situation is resolved.

When the study has been permanently closed to patient enrollment, submission of annual IRB approvals to the Registration Office is no longer necessary.

5.2.3.2 Prior to accepting the registration, registration/randomization application will verify the following:

- IRB approval at the registering institution
- Patient eligibility
- Existence of a signed consent form
- Existence of a signed authorization for use and disclosure of protected health information

5.2.3.3 At the time of registration, the following will be recorded:

- Patient has/has not given permission to store and use his/her sample(s) for future research of cancer.
- Patient has/has not given permission to store and use his/her sample(s) for future research to learn, prevent, or treat other health problems.
- Patient has/has not given permission for someone to contact them about future participation in more research
- Patient has/has/not given permission to have coded genetic information and coded medical information placed in password protected, secured database for research analyses.

5.2.3.4 Treatment on this protocol must commence at a TBCRC institution under the supervision of a medical oncologist.

5.2.3.5 Treatment cannot begin prior to randomization and must begin **≤7** days after randomization.

5.2.3.6 Pretreatment tests/procedures (see Section 11) must be completed within the guidelines specified on the test schedule. Prior to registration/ randomization a research biopsy of metastatic breast tissue must be taken for central pathologic review.

5.2.3.7 All required baseline symptoms must be documented and graded.

5.2.3.8 Study drug is available on site.

5.2.3.9 Blood draw kit is available on site.

5.3 Randomization Procedures

All institutions

5.3.1 The factors defined in Section 17.3, together with the registering membership, will be used as stratification factors.

5.3.2 After the patient has been registered into the study, the values of the stratification factors will be recorded, and the patient will be assigned to one of the following treatment groups using the Pocock and Simon dynamic allocation procedure[26] which balances the marginal distributions of the stratification factors between the treatment groups.

- *Alisertib alone*
- *Alisertib in combination with Fulvestrant*

5.4 Optional Crossover Phase

5.4.1 Patients in Arm 1 who meet the criteria in Section 6.1.2 are eligible to crossover.

5.4.2 If the patient and physician want to cross over to Arm 2, fax (507-284-0885) a completed continuation phase eligibility checklist to the Registration Office between 8 a.m. and 4:30 p.m. central time Monday through Friday.

5.4.3 Treatment cannot begin prior to registering to the crossover phase and will ideally begin ≤7 days after registration for the crossover phase.

6. TREATMENT PLAN

For both treatment arms:

- Study treatment by a local medical doctor (LMD) is not allowed. Routine and acute clinical care may be provided by LMD and reported to treating institution.
- If there is any interruption of treatment of ≥21 days contact Study Chair to determine whether the patient may restart treatment or will enter the event monitoring phase of the study.
- If hepatic panel on C1D1 indicates progressive liver involvement (increase in total bilirubin to >1.5 x ULN or increase in ALT to >5 x ULN), then patient is not allowed to start study treatment.
  Note to study staff: A comment should be added to the CRF stating that the patient had unacceptable liver enzyme levels on Cycle 1 Day 1 and did not start treatment.
- If CBC on C1D1 indicates new or worsening cytopenia(s), then starting dose of alisertib may be reduced based on discussion with overall study chair (Dr. Haddad).
- Patients who have achieved complete response, partial response, or stable disease for ≥2 years may reduce imaging to every fourth cycle.

6.1 Arm 1 (Alisertib Monotherapy)

One treatment cycle=28 (±3) days

Alisertib Monotherapy Alone

Agent	Dose	Schedule	Route	Retreatment
Alisertib	50 mg BID*	Days 1-3, 8-10, 15-17	orally	every 28 (±3) days until disease progression

*Doses should be taken 12 hours apart with a minimum of 6 hours in between

Note: Cycle 1 Day15: The morning dose of alisertib should be taken prior to Cycle 1 Day15 blood draws

6.1.1 Patients randomized to Arm 1 will initially receive alisertib until progression (PD) or intolerability.

6.1.2 At the time of progression on alisertib treatment, patients will be allowed to add fulvestrant to alisertib if all 3 of the following conditions (a thru c) are met:

a) Pre-registration biopsy finding of ER+ disease
OR
Pre-registration biopsy finding of ER <10% positive or ER negative disease and a biopsy performed at the time of progression on alisertib demonstrates ER+ ≥10% disease

b) Recovered from any non-hematologic toxicities to ≤Grade 1 and blood chemistries are such that

- Absolute neutrophil count (ANC) ≥1500/mm^3
- Platelet count ≥100,000/mm^3
- Hemoglobin >9.0 g/dL
- Total bilirubin ≤ULN
  NOTE: If total bilirubin is out of range then direct bilirubin must be ≤ULN
- Alanine transaminase (ALT) ≤2.5 x ULN(≤5 x ULN for patients with liver involvement)
- Calculated creatinine clearance ≥45 ml/min

c) Treatment with alisertib and fulvestrant must start at most 35 days after documentation of disease progression on alisertib alone

6.1.2.1 Patients who are not eligible or choose not to continue on with the combination of fulvestrant and alisertib will go to Event Monitoring (see Section 14.0).

6.1.3 Crossover Combination Therapy

One treatment cycle=28 (±3) days

6.1.3.1 On the days both fulvestrant and alisertib are given, alisertib should be taken before fulvestrant is administered.

Agent	Dose	Schedule	Route	Retreatment
Fulvestrant	500 mg	Day 1 and Day 15 of 1st crossover cycle; Day 1 of all subsequent cycles	intramuscular injection	every 28 (±3) days until disease progression
Alisertib	at dose level last used when given as a single agent*	Days 1-3, 8-10, 15-17	orally	

* Doses should be taken 12 hours apart with a minimum of 6 hours in between

Note: Cycle 1 Day15: The morning dose of alisertib should be taken prior to Cycle 1 Day15 blood draws.

6.2 Arm 2 (Alisertib with Fulvestrant)

One treatment cycle=28 (±3) days

6.2.1 Patients randomized to Arm 2 will receive the combination of fulvestrant and alisertib until progression (PD) or intolerability and then enter the event monitoring phase of the protocol.

6.2.2 Patients will receive a loading dose of fulvestrant unless fulvestrant was the most recent therapy prior to registration and most recent dose was ≤30 days and >21 days prior to C1D1.

6.2.3 On the days both fulvestrant and alisertib are given, alisertib should be taken before fulvestrant is administered.

Agent	Dose	Schedule	Route	Retreatment
Fulvestrant	500 mg	Day 1 and Day 15 of Cycle 1<br>Day 1 of Cycle 2 and all cycles thereafter	intramuscular injection	every 28 (±3) days until disease progression
Alisertib	50 mg BID*	Days 1-3, 8-10, 15-17	orally	every 28 (±3) days until disease progression

* Doses should be taken 12 hours apart with a minimum of 6 hours in between

Note: Cycle 1 Day15: The morning dose of alisertib should be taken prior to Cycle 1 Day15 blood draws.

6.3 Concomitant Treatment and Supportive Care Guidelines

6.3.1 Pre-treatment anti-emetics are not recommended prior to Cycle 1. Supportive care pre-treatment medications may be added with subsequent cycles per discretion of the treating oncologist.

6.3.2 Blood products and growth factors should be utilized as clinically warranted and following institutional policies and recommendations. The use of growth factors should follow published guidelines of the American Society of Clinical Oncology (42) 2006 Update of Recommendation for the Use of White Blood Cell Growth Factors: An Evidence-Based Clinical Practice Guideline Journal of Clinical Oncology 2006; 24(19):3187-3205.

6.3.3 Patients should receive full supportive care while on this study. This includes blood product support, antibiotic treatment, and treatment of other newly diagnosed or concurrent medical conditions. All blood products and concomitant medications such as antidiarrheals, analgesics, and/or antiemetics received from the first day of study treatment administration until 30 days after the final dose will be recorded in the medical records.

6.3.4 Diarrhea: This event could be managed conservatively with loperamide. The recommended dose of loperamide is 4 mg at first onset, followed by 2 mg every 2-4 hours until diarrhea free (maximum 16 mg/day).

In the event of Grade 3 or 4 diarrhea, the following supportive measures are allowed: hydration, octreotide, and antidiarrheals.

If diarrhea is severe (requiring intravenous rehydration) and/or associated with fever or severe neutropenia (Grade 3 or 4), broad-spectrum antibiotics must be prescribed. Patients with severe diarrhea or any diarrhea associated with severe nausea or vomiting **should be hospitalized** for intravenous hydration and correction of electrolyte imbalances.

6.3.5 Zoledronic acid, pamidronate, or denosumab may be administered per institutional guidelines as appropriate for patients with bone metastases.

6.3.6 PPI and histamine-2 (H2) receptor antagonists are not permitted.

6.3.7 Neutralizing antacids and calcium-containing supplements cannot be taken from 2 hours prior to alisertib dosing until up to 2 hours after dosing.

6.3.8 Concomitant use of moderate or strong CYP3A4 inhibitors (including but not limited to: clarithromycin, itraconazole, voriconazole, ketoconazole,

posaconazole, nefazodone, telithromycin, aprepitant, erythromycin, fluconazole, verapapmil, diltiazem) or grapefruit juice/grapefruit-containing products should be avoided. Selection of an alternate concomitant medication with no or minimal CYP3A4 inhibition potential is recommended. If co-administration of moderate or strong CYP3A4 inhibitors is medically necessary, investigators should monitor for toxicities and follow the dose modifications per study protocol.

6.4 Treatment/Follow-up Decision at Evaluation of Patient

6.4.1 Patients who have not had systemic disease progression and have experienced acceptable toxicity will be eligible for re-treatment at their current dose level until disease progression, unacceptable toxicity, or refusal.

6.4.2 Those patients who have not progressed and who have experienced unacceptable toxicity may be eligible for re-treatment at a lower dose. See Section 7.

6.4.3 Patients randomized to the alisertib alone arm that progress may begin treatment with fulvestrant and alisertib if the conditions listed in Section 6.1 are met.

Patients who are not eligible to continue on to fulvestrant plus alisertib or who choose not to continue on to fulvestrant plus alisertib will proceed to the Event Monitoring phase where patients will be followed every 6 months until death or a maximum of 5 years post-randomization for disease progression and death information.

Patients who proceed with fulvestrant plus alisertib treatment will continue until: disease progression, patient request, intolerability, or refusal.

6.4.4 Criteria for discontinuation of all protocol therapy include:

- Tumor progression during combination treatment with fulvestrant and alisertib
- Arm 1 only: tumor progression during alisertib and patient will not be switching to combination treatment with fulvestrant and alisertib
- Request by the patient to withdraw from all protocol treatment
- Unacceptable adverse events
- Treatment delay of >21 days (exception: recovery from Cycle 1 Day 15 severe neutropenia where treatment delay of >30 days triggers treatment discontinuation [see Section 7.3])
- Intercurrent illness which would, in the judgment of the investigator, affect assessments of clinical status to a significant degree that require discontinuation of drug
- Administration of radiotherapy, non-protocol chemotherapy, or an experimental drug during the trial

Patients who discontinue treatment for any of the above reasons will go to event monitoring. Once a patient has entered the event monitoring phase of the trial, his/her therapy is at the discretion of the treating physician.

6.4.5 If an eligible patient refuses treatment following registration (and is classified as a "cancel" by the Research Base), all on-study materials must be submitted to the Research Base.

6.4.6 A patient is deemed *ineligible* if at the time of registration, the patient did not satisfy each and every eligibility criteria for study entry.

- If the patient never received any study treatment, on-study materials (with the exception of biospecimens) must be submitted. No further follow-up is required.
- If the patient is receiving study treatment at the time the ineligibility is discovered, the patient may continue on study at the discretion of the treating physician as long as there are no safety concerns, and the patient was properly registered. All protocol procedures must be followed. Otherwise, the patient enters the event monitoring phase of the study.

6.4.7 If protocol requirements regarding treatment in Cycle 1 of the initial therapy are severely violated to the point the Study Team finds that the patient is not evaluable for primary end point, the patient will be flagged in to study database as a protocol violation. The patient may continue on study at the discretion of the treating physician as long as there are no safety concerns, and the patient was properly registered. All protocol procedures must be followed. Otherwise, no further follow-up is required.

6.4.8 If a patient withdraws consent prior to receiving any study treatment or is found to have unacceptable liver enzyme levels [either (1) total bilirubin >1.5 x ULN or (2) alanine transaminase >5 x ULN] on Cycle 1 Day 1 and does not receive protocol treatment, the patient will be deemed a *cancel.* On-study material (with the exception of biospecimens) and the End of Active Treatment/Cancel Notification Form must be submitted. For patients who are cancelled due to unacceptable liver enzyme levels on Cycle 1 Day 1. A comment should be added to the CRF stating that the patient had unacceptable liver enzyme levels on Cycle 1 Day 1 and did not start treatment. No further data submission is necessary.

7. EXPECTED TOXICITIES AND DOSING DELAYS/DOSE MODIFICATIONS

7.1 Dose Modification Based on Adverse Events

7.1.1 Dose modifications based on Adverse Event documented prior to each cycle of treatment. Strictly follow the modification in the table provided below until individual treatment tolerance can be ascertained. If multiple adverse events occur, administer the dose based on the greatest dose reduction required for any single adverse event observed. Dose modifications apply to the treatment given in the preceding cycle and are based on adverse events observed in the prior dose.

7.1.2 Note that fulvestrant administration is not modified for any adverse event.

- If alisertib is held due to toxicity then fulvestrant should also be held. Both agents should be restarted together.
- If alisertib is discontinued due to intolerability then fulvestrant is also discontinued.

7.1.3 If treatment is held greater than 21 days without resolution of any adverse events other than severe neutropenia or a 3rd dose reduction is required then study treatment is to be discontinued* and the patient will enter the Event Monitoring phase of the study.(see Section 14.0).
*Note: If patient is deriving benefit from study treatment and the event can be managed safely, consult Study Chair (Dr. Haddad) regarding continuation.

7.1.4 Dose reduction levels

Dose Level	Alisertib (twice daily) Days 1-3, 8-10, 15-17
-1	40 mg
-2	30 mg

→ → Use the NCI Common Terminology Criteria for Adverse Events (CTCAE) version 4.0* unless otherwise specified ← ←

* Located at http:/ctep.cancer.gov/protocolDevelopment/electronic_applications.ctc.htm

7.2 Dose Modifications – Prior to each cycle of treatment

Strictly follow the modification in the table provided below until individual treatment tolerance can be ascertained. If multiple adverse events occur, administer the dose based on the greatest dose reduction required for any single adverse event observed. Dose modifications apply to the treatment given in the preceding cycle and are based on adverse events observed in the prior dose**.**

CTCAE System/ Organ/ Class (SOC)	ADVERSE EVENT	ACTION**
Blood and lymphatic system disorders	Anemia Grade 3 with hemoglobin <7.0 g/dL or Grade 4	First or Second Episode: Hold alisertib administration until recovery to ≤Grade 2 or baseline then decrease by 1 dose level<br>If not recovered within 14 days discontinue alisertib and proceed to event monitoring phase*<br>Third Episode: Discontinue study treatment (see note*).<br>*NOTE: If patient is deriving benefit from study treatment and event can be managed safely, consult Study Chair regarding continuation.
Investigations	Neutrophil Count Decreased Grade 3 (neutrophils <1000 - 500 cells/mm^3)	First Episode: Hold alisertib administration and check neutrophil counts within 5-7 days of report of Grade 3 ANC (referred to as ANC recheck #1).<br>(a) results of ANC recheck #1<br>– If neutrophils ≥1500 cells/mm^3, resume alisertib at current dose<br>– If neutrophils <1500 cells/mm^3, continue to hold alisertib and re-check neutrophils counts again within the next 7 days but at most 14 days after the first report of Grade 3 ANC (ANC recheck #2)<br>(b) results of ANC recheck #2<br>▪ If neutrophils ≥1500 cells/mm^3, resume alisertib at one dose lower<br>▪ If neutrophils remain <1500 cells/mm^3 14 days after first report of Grade 3 ANC , discontinue study treatment*<br>Second Episode: Hold alisertib administration and check neutrophil count within 5-7 days of report of Grade 3 ANC (referred to as ANC recheck #1).<br>(a) results of ANC recheck #1<br>– If neutrophils ≥1500 cells/mm^3, resume alisertib at first dose level below current dose level<br>– If neutrophils <1500 cells/mm^3, continue to hold alisertib and re-check neutrophils counts again within the next 7 days but at most 14 days after the first report of Grade 3 ANC (ANC recheck #2)<br>(b) results of ANC recheck #2<br>▪ If neutrophils ≥1500 cells/mm^3, resume alisertib at first dose level below current dose<br>▪ If neutrophils remain <1500 cells/mm^3 14 days after first report of Grade 3 ANC in this second episode, discontinue study treatment*<br>Third Episode: discontinue study treatment (see note*)<br>*NOTE: If patient is deriving benefit from study treatment and event can be managed safely, consult Study Chair regarding continuation.

Investigations	Neutrophil count decreased Grade 4 (neutrophils <500 cells/mm^3)	First Episode: Hold alisertib administration and check neutrophil counts within 5-7 days of report of Grade 4 ANC (referred to as ANC recheck #1).<br>(a) results of ANC recheck #1<br>▪ If neutrophils ≥1500 cells/mm^3, resume alisertib at first dose level below at current dose<br>▪ If neutrophils <1500 cells/mm^3, continue to hold alisertib and re-check neutrophils counts again within the next 7 days but at most 14 days after the first report of Grade 4 ANC (ANC recheck #2)<br>(b) results of ANC recheck #2<br>▪ If neutrophils ≥1500 cells/mm^3, resume alisertib at first dose level below at current dose<br>▪ If neutrophils remain <1500 cells/mm^3 14 days after first report of Grade 4 ANC , discontinue study treatment*<br>Second Episode: Hold alisertib administration and check neutrophil counts within 5-7 days of report of Grade 4 ANC results of ANC recheck #1<br>(a) results of ANC recheck #1<br>▪ If neutrophils ≥1500 cells/mm^3, resume alisertib at first dose level below current dose<br>▪ If neutrophils <1500 cells/mm^3, continue to hold alisertib and re-check neutrophils counts again within the next 7 days but at most 14 days after the first report of Grade 4 ANC (ANC recheck #2)<br>(b) results of ANC recheck #2<br>▪ If neutrophils ≥1500 cells/mm^3, resume alisertib at first dose level below at current dose<br>▪ If neutrophils remain <1500 cells/mm^3 14 days after first report of Grade 4 ANC , discontinue study treatment*<br>Third Event: Discontinuestudy treatment (see note*)<br>*NOTE: If patient is deriving benefit from study treatment and event can be managed safely, consult Study Chair regarding continuation.
Investigations	Platelet count decreased Grade 1 and 2 (<100,000 cells/mm^3 but >50,000 cells/mm^3)	Hold alisertib administration and check platelet counts every 3-5 days until platelets ≥100,000 cells/mm^3 or for a maximum of 21 days after Grade 1-2 decrease in PLT reported<br>If platelet count recovered to ≥100,000 cells/mm^3 within 21 days then resume alisertib at current dose<br>Otherwise discontinue study treatment *<br>*NOTE: If patient is deriving benefit from study treatment and event can be managed safely, consult Study Chair regarding continuation

Investigations	Platelet count decreased Grade 3 (25,000 – 50,000 cells/mm³)	First Episode: Hold alisertib administration and check neutrophil counts within 5-7 days of report of Grade 3 PLT (referred to as PLT recheck #1)<br>(a) results of PLT recheck #1<br>▪ If platelets ≥100,000 cells/mm³, resume alisertib at current dose<br>▪ If platelets <100,000 cells/mm³, continue to hold alisertib and re-check platelet count again within the next 5- 7 days but at most 14 days after the first report of Grade 3 ANC (ANC recheck #2)<br>(b) results of PLT recheck #2<br>▪ If platelets ≥100,000 cells/mm³, resume alisertib at first dose level below at current dose<br>▪ If platelets remains <100,000 cells/mm³ 14 days after first report of Grade 3 PLT decrease, discontinue study treatment<br>Second Episode: Hold alisertib administration and check platelet counts within 5-7 days of report of Grade 1 ANC results of ANC recheck #1<br>(a) results of PLT recheck #1<br>▪ If platelets ≥100,000 cells/mm³, resume alisertib at current dose<br>▪ If platelets <100,000 cells/mm³, continue to hold alisertib and re-check platelet counts again within the next 7 days but at most 14 days after the first report of Grade 3 PLT (PLT recheck #2)<br>(b) results of PLT recheck #2<br>▪ If platelets ≥100,000 cells/mm³, resume alisertib at first dose level below at current dose<br>▪ If platelets remains <100,000 cells/mm³ 14 days after first report of Grade 3 PLT decrease, discontinued 14 days after first report of Grade 3 ANC, discontinue study treatment<br>Third Event: Discontinue all study treatment (see note*)<br>*NOTE: If patient is deriving benefit from study treatment and event can be managed safely, consult Study Chair regarding continuation.
	Platelet count decreased Grade 4 (<25,000/mm³) or Grade 3 with bleeding	Discontinue all study treatment*<br>*NOTE: If patient is deriving benefit from study treatment and event can be managed safely, consult Study Chair regarding continuation.

Blood and lymphatic system disorders	Febrile neutropenia<br>Grade 3<br>(neutrophils <1000 cells/mm^3 with a single temperature of >38.3°C (101°F) or a sustained temperature of ≥38°C (100.4°F) for more than one hour)<br>Grade 4<br>(Life threatening consequences)	Discontinue all study treatment*<br>*NOTE: If patient is deriving benefit from study treatment and event can be managed safely, consult Study Chair regarding continuation.
Gastrointestinal disorders	Mucositis oral Grade 3 or 4	First event:<br>Hold alisertib administration until recovered to ≤Grade 1 or pre-treatment level<br>If recovered Grade 1 or baseline within 21 days, continue alisertib one dose level below current dose level<br>Second event:<br>Hold alisertib administration until recovered to ≤Grade 1 or pre-treatment level<br>If recovered to ≤Grade 1 or baseline within 21 days, continue alisertib one dose level below current dose level<br>If not, discontinue all study treatment*<br>Third event: Discontinue all study treatment (see note*)<br>*NOTE: If patient is deriving benefit from study treatment and event can be managed safely, consult Study Chair regarding continuation.
Nervous system disorders	Somnolence ≥Grade 3	Hold alisertib administration until recovered to ≤Grade 1 or pre-treatment level<br>If recovered to ≤Grade 1 or baseline within 14 days, continue alisertib one dose level below current dose level<br>If not, discontinue all study treatment (see note*)<br>NOTE: Evaluate whether patient alcohol consumption or utilization of benzodiazepines are contributing. If so, counsel patient to restrict alcohol and discontinue benzodiazepines with provider guidance.<br>*NOTE: If patient is deriving benefit from study treatment and event can be managed safely, consult Study Chair regarding continuation
Other unspecified adverse event reduction instructions	≥Grade 3	Hold alisertib administration until recovered to ≤Grade 1 or pre-treatment level (baseline level)<br>If recovered to ≤Grade 1 or baseline within 14 days, continue alisertib one dose level below current dose level<br>If not, discontinue all study treatment *<br>*NOTE: If patient is deriving benefit from study treatment and event can be managed safely, consult Study Chair regarding continuation

7.3 Dose Modifications – Cycle 1 Day 15

The following dose modifications apply to adverse events documented on Cycle 1 Day 15 and the first crossover Cycle 1 Day 15.

Investigations	Neutrophil count decreased<br>Grade 3 or 4 (neutrophils <1000 cells/mm^3)	Omit Cycle 1 Days 15-17 administration of alisertib<br>Administer fulvestrant loading dose<br>Check neutrophil count on Cycle 2 Day 1

8. DRUG FORMULATION/STORAGE/SUPPLY

8.1 Alisertib (MLN8237, ML00653668)

Alisertib is an investigational agent and will be supplied free-of-charge from Millennium.

8.1.1 **Background**: Alisertib is a selective small molecule inhibitor of Aurora A kinase inhibitor that is being developed for the treatment of advanced malignancies. Aurora A is expressed and active during mitosis. Alisertib is also expected to be toxic to proliferating normal tissues, such as the bone marrow, GI epithelium, and hair follicles, since any cell that is in mitosis, where Aurora A is expressed and active, should be susceptible to the effects of an Aurora A kinase inhibitor. The Aurora A kinase gene is amplified, overexpressed, or both, in many tumors including: colon, breast, pancreatic, and bladder cancers, as well as certain lymphomas, leukemias, and myeloma.

8.1.2 **Formulation**: Alisertib is supplied as enteric-coated tablets, formulated with the sodium salt of MLN8237 (MLN8237-004).

The enteric-coated tablet (ECT) dosage form of alisertib is available as 10 and 15 mg strengths, expressed as MLN8237 free acid. The key formulation excipients that aid in the in vivo absorption of the drug are sodium bicarbonate, sodium lauryl sulfate, and enteric coating. The other formulation excipients such as povidone, microcrystalline cellulose, croscarmellose sodium, and sodium stearyl fumarate serve as manufacturing aids.

Open-label investigational product will be labeled to fulfill all requirements specified by applicable governing regulations.

8.1.3 **Preparation and storage**: Alisertib is a cytotoxic compound, and should be handled with caution. Sites should follow institutional hazardous substance procedures for handling, disposal and spill clean-up.

Enteric-coated tablets are packaged in 60-cc HDPE bottles with rayon coil, induction seal, desiccant packs and polypropylene child-resistant caps.

The oral solution is filled in 2-oz. amber polyethylene terephthalate bottles, sealed with 24-mm polypropylene child-resistant caps and polyester liners.

Alisertib should be stored at controlled room temperature (20-25ºC) with excursions permitted to 15-30 ºC, and used before the retest date provided by the manufacturer.

8.1.4 **Administration:** Retain alisertib in the bottle provided until use. Alisertib should be administered as provided and is not intended to be manipulated in any way. Alisertib is a cytotoxic anticancer drug. As with other potentially toxic compounds, caution should be exercised when handling. It is recommended that gloves and protective garments be worn during preparation when dispensed in the clinic.

Alisertib may be administered regardless of the timing of meals, unless otherwise specified in the protocol. The dose should be swallowed with 250 mL (about 1 cup) of water. Twice daily doses should ideally be taken every 12 hours, and doses are required to be separated by a minimum of 6 hours. Vomited doses will **not** be made up. Patients will take their next dose at the regularly scheduled time.

8.1.5 **Pharmacokinetic information**: Pharmacokinetics properties of alisertib in patients with hematologic malignancies are generally consistent with those observed in patients with non-hematologic malignancies.

a) Absorption –Clinical development has transitioned to the enteric-coated tablet formulation which is designed to bypass the stomach and delay dissolution until delivery to the upper small intestine, due to reduced solubility of an acidic drug in acidic pH. ECT absorption is fast with an overall median T_{max} of 2.5 hours post-dose.
b) Distribution – Alisertib is highly bound to plasma proteins with percentage of unbound alisertib measured at 2.5% in human plasma. Based on the results of in vitro blood-to-plasma partitioning studies in human whole blood, the mean blood-to-plasma ratio was 0.57, indicating preferential distribution into human plasma.
c) Metabolism – Alisertib is metabolized by both oxidation and glucuronidation pathways, and multiple cytochrome P450 (CYP) isozymes (CYP3A4, 2C8, 2C9, 2C19, and1A2) and uridine diphosphate-glucuronosyltransferase (UGT) isozymes (UGT1A1, 1A3, and 1A8) are involved. Phase 1 oxidative metabolism was identified to be of quantitative importance, with an estimated contribution of CYP3A4 of approximately 60% to overall apparent oral clearance in humans. Therefore, moderate to strong inhibitors of CYP3A4 and clinically significant inducers may alter systemic exposures of alisertib. Given a partial contribution of UGT1A1 to the metabolism of alisertib based on in vitro studies, genotyping for the reduced expression allele UGT1A1 *28 has been prospectively performed in the clinical program across phase 1 and phase 2 studies. The results of these analyses indicate that pharmacogenetic variation in UGT1A1 is not a meaningful contributor to the variability in the clinical PK of alisertib.
d) Excretion – Overall mean steady-state terminal half-life following multiple-dose administration of enteric coated tablets in patients with non-hematologic

malignancies was approximately 21 hours. Preliminary results indicate the mean terminal half-life for the oral solution is 22 hours. Negligible urinary excretion of alisertib was observed in humans. The predominant route of elimination for alisertib was fecal, consistent with hepatic metabolism and biliary excretion of alisertib.

8.1.6 **Potential Drug Interactions**: Oxidative metabolism is the predominant route of elimination for alisertib. Results of subsequent in vitro drug metabolism studies have shown that the oxidative metabolism of alisertib is mainly mediated via CYP3A4. Study results have shown that co-administration of alisertib with rifampin (a strong inducer of CYP3A), and itraconazole (a strong inhibitor of CYP3A) have a significant effect on the systemic exposure of alisertib. Therefore, it is recommended that the concomitant use of strong CYP3A inhibitors (e.g., ketoconazole, itraconazole, clarithromycin, conivaptan, nefazodone, telithromycin, voriconazole, posaconazole, boceprevir, telaprevir, and grapefruit juice/grapefruit containing products) and strong CYP3A inducers (e.g., rifampin, enzyme-inducing antiepileptic agents), be avoided in patients receiving alisertib. Selection of an alternate concomitant medication with no or minimal CYP3A inhibition potential is recommended. If co-administration of strong CYP3A inhibitors is medically necessary, investigators should monitor for toxicities and follow the dose modifications for toxicity per study protocol.

Alisertib exhibits pH-dependent solubility with decreased solubility under acidic pH conditions. Results from a completed study evaluating the effect of esomeprazole on alisertib PK indicated that co-administration of alisertib with esomeprazole resulted in a 28% increase in total systemic exposure of alisertib. Co-administration with drugs that alter intragastric pH (e.g. PPIs, H2 antagonists) is not permitted in ongoing clinical studies. Studies also prohibit concomitant use of pancreatic enzymes or conditions (such as pancreatectomy) that could potentially impact drug absorption in the upper bowel.

8.1.7 **Known potential toxicities**:
Very common (>10%): anemia, febrile neutropenia, leukopenia, neutropenia, thrombocytopenia, abdominal pain, diarrhea, nausea, oral pain, stomatitis, vomiting, asthenia, fatigue, pyrexia, decreased appetite, dehydration, somnolence, disorientation (and associated memory loss), alopecia

Common (≥1% and <10%): lymphopenia, pancytopenia, dyspepsia, mucosal inflammation, URTI, UTI, pneumonia, oral candidiasis, sepsis (including fatal sepsis), cellulitis, oral herpes, sinusitis, nasopharyngitis, bronchitis, herpes zoster, influenza, conjunctivitis, esophagitis, skin infection, fungal skin infection, oral fungal infection, septic shock, increased AST, increased ALT, increased blood bilirubin, increased alkaline phosphatase, increased GGT, gait disturbance, confusional state, rash, pruritic rash, macular rash, maculo-papular rash, dermatitis acneiform, palmar-plantar erythrodysesthesia syndrome

Uncommon (≥0.1% and <1%): sedation, dermatitis bullous

Reproductive toxicology studies have not been conducted with alisertib. Given the outcomes associated with Aurora A kinase inhibition and the effects of alisertib observed in reproductive tissues in repeat-dose toxicology studies, it is anticipated that alisertib could be a reproductive and developmental toxicant.

Because alisertib may cause anemia, neutropenia, leukopenia, lymphopenia, and thrombocytopenia, patients on alisertib may experience the anticipated sequelae of infection, hemorrhage, or bleeding.

Because of the effects of alisertib on the gastrointestinal mucosa, there is a potential risk of mucosal bleeding, GI bleeding, and the associated infections and sepsis.

Cases of cardiac abnormalities have been infrequently observed in patients receiving treatment with alisertib, including decreased left ventricular ejection fraction, tachycardia, and 1 case of QT prolongation. The causal relationship between cardiac dysfunction and alisertib has been confounded by patients' prior history of other cardiac abnormalities.

Alisertib is structurally related to the benzodiazepines and may cause somnolence, confusion, dizziness or gait disturbance associated with the onset of maximal plasma concentration (about 3 hrs post-dose). For this reason, patients should not drive, operate dangerous machinery, or perform any other task requiring full alertness and/or coordination if these effects are experience while enrolled on study. Sedative effects have been managed by administration of divided doses and dose administration at bedtime, although dose reductions have sometimes been required.

8.1.8 **Drug procurement and ordering:**

Open-label alisertib will be provided free of charge to patients by Takeda/Millennium Pharmaceuticals, Inc.

Each institution will order the drug through drug suppliers. (See Pharmacy Manual for order form.)

NOTE: Local drug destruction may occur if the site provides a copy of their local drug destruction policy to the sponsor (Mayo Clinic) and the respective drug suppliers (Takeda/Millennium).

8.1.9 **Nursing Guidelines:**

8.1.9.1 Cytopenias have been seen with this agent. Monitor CBC and instruct patients to report any signs or symptoms of infection and/or abnormal bruising or bleeding to the study team.

8.1.9.2 GI side effects, including abdominal pain, diarrhea, dyspepsia, nausea, and vomiting have been seen. Treat symptomatically and assess for effectiveness.

8.1.9.3 Warn patients that they may experience somnolence, confusion or memory loss. Patient should be instructed not to operate a motor vehicle,

heavy machinery or participate in other activities that require mental sharpness until they know how this agent affects them.

8.1.9.4 Instruct patients that they may experience alopecia.

8.1.9.5 Monitor LFTs. Report increases in AST, ALT, bilirubin, GGT and Alk Phos to MD. Rarely hepatic veno-occlusive disease is seen.

8.1.9.6 Patients may experience palmar plantar erythrodysesthesia (hand-foot syndrome). Instruct patients to report any redness, pain, or skin issues of the hands or feet to the study team.

8.1.9.7 Alisertib can be administered regardless of the timing of food, unless otherwise specified. BID dosing should be taken every 12 hours and must be separated by at least 6 hours. Additionally doses should be swallowed with 250 mL (about 1 cup) of water and vomited doses should not be made up.

8.1.9.8 Neutralizing antacids should not be taken until 2 hours after alisertib is administered.

8.1.9.9a Gloves and protective garments should be worn during preparation and when dispensed in the clinic.

8.1.9.9b Do not open, crush or manipulate the pills in any way.

8.1.9.9c Alisertib can be given regardless of meals, unless the protocol dictates otherwise. Pills should be taken with at least 250 ml (1 cup) of water. Vomited doses should not be made up. Instruct patients that they should take their next dose at the regularly scheduled time.

8.2 Fulvestrant (Faslodex®)

Fulvestrant is commercially available.

8.2.1 Background

Fulvestrant is an estrogen receptor antagonist. Fulvestrant competitively binds to estrogen receptors on tumors and other tissue targets, producing a nuclear complex that causes a dose-related down-regulation of estrogen receptors and inhibits tumor growth.

8.2.2 Formulation, preparation and storage

Commercially available for injection as: Injection, solution: 50 mg/mL (5 mL) [contains benzyl alcohol, benzyl benzoate, castor oil, ethanol 10% w/v]

8.2.3 **Preparation and storage**

Refer to package insert for complete preparation and dispensing instructions. Store in original carton under refrigeration at 2°C to 8°C (36°F to 46°F). Protect from light.

8.2.4 **Administration**

Refer to the treatment section for specific administration instructions. Administer IM only; do not administer IV, SubQ, or intra-arterially. Administer 500 mg dose as two 5 mL injections (one in each buttocks) slowly over 1-2 minutes per injection.

8.2.5 **Pharmacokinetic information**
Distribution: V_d: ~3-5 L/kg
Protein binding: 99%; to plasma proteins (FLDL, LDL and HDL lipoprotein fractions)
Metabolism: Hepatic via multiple biotransformation pathways (CYP3A4 substrate involved in oxidation pathway, although relative contribution to metabolism unknown); metabolites formed are either less active or have similar activity to parent compound.
Half-life elimination: 250 mg: ~40 days
Excretion: Feces (~90%); urine (<1%)

8.2.6 **Potential Drug Interactions**
Cytochrome P450 Effect: Substrate of CYP3A4 (minor). There are no known interactions where it is recommended to avoid concomitant use.

8.2.7 **Known potential toxicities**
Consult the package insert for the most current and complete information.

Common known potential toxicities, >10%
Endocrine & metabolic: Fertility; Hot flushes
Hepatic: Alkaline phosphatase increased, transaminases increased
Local: Injection site pain
Neuromuscular & skeletal: Joint disorders

Less common known potential toxicities, 1% - 10%
Cardiovascular: Ischemic disorder
Central nervous system: Fatigue, headache
Gastrointestinal: Nausea, anorexia, vomiting, constipation, weight gain
Genitourinary: Urinary tract infection
Neuromuscular & skeletal: Bone pain, arthralgia, back pain, extremity pain, musculoskeletal pain, weakness
Respiratory: Cough, dyspnea

Rare known potential toxicities, <1% (Limited to important or life-threatening)
Angioedema, hypersensitivity reactions, leukopenia, myalgia, osteoporosis, thrombosis, urticaria, vaginal bleeding

8.2.8 Drug procurement and ordering

Commercial supplies. Pharmacies or clinics shall obtain supplies from normal commercial supply chain or wholesaler.

8.2.9 Nursing Guidelines

8.2.9.1 Rarely a blood-tinged vaginal discharge has been reported infrequently during therapy. Advise patients that this is a possibility and that studies have shown no effects on vagina or endometrium.

8.2.9.2 Monitor LFTs based on protocol requirements. Report increased LFTs to treating physician.

8.2.9.3 Hot packing the injection site for a short while after injection may prevent the possible bruising, tenderness, and/or erythema at the IM injection site.

8.2.9.4 It is recommended that pregnancy be avoided during treatment. Instruct patient and partner on adequate methods of birth control, if needed.

8.2.9.5 Instruct patient to report any signs or symptoms of blood clots. Patients with calf tenderness or burning, and/or chest pain (pulmonary embolus) should be evaluated by a physician immediately.

8.2.9.6 Warn patient of possible hot flashes. Assess severity and discuss non-hormonal treatments with treating physician. Assess treatment for efficacy.

8.2.6.7 May cause mild GI symptoms (nausea, anorexia, vomiting, constipation). Treat symptomatically and monitor for effectiveness of intervention.

9. CORRELATIVE/SPECIAL STUDIES

9.1 Body Fluid Biospecimens

9.1.1 Summary of the blood biospecimen collection schedule

9.1.1.1 All Patients

	Cycle 1, Day 1 prior to start of treatment (Baseline)	At the end of Cycle 1 (prior to start of Cycle 2)	At time of disease progression	Submit to:	Shipping/storage instructions
**CTC**	One 10 mL blood sample collected in a CellSave® tube and one 10 mL blood sample collected in an AccuCyte tube	One10 mL blood sample collected in CellSave® tube and one 10 mL blood sample collected in an AccuCyte tube	One 10 mL blood sample collected in CellSave® tube and one 10 mL blood sample collected in an AccuCyte tube	BAP freezer Mayo Clinic	Samples should be kept at ambient temperature after collection and during shipping. Specimens must arrive at Mayo within 72 hours of collection.
**cfDNA**	Two 10 mL blood samples collected in Streck® tubes	Two 10 mL blood samples collected in Streck® tubes	Two 10 mL blood samples collected in Streck® tubes	BAP freezer Mayo Clinic	Samples should be kept at ambient temperature after collection and during shipping. Specimens must arrive at Mayo within 72 hours of collection.
**SNP**	One 6 mL blood sample collected in a K2 EDTA tube. Gently invert tube 6-8 times - Do not centrifuge	-	-	BAP Mayo Clinic	Upon processing, refrigerate until shipment; ship refrigerated

**9.1.1.2 For those patients with crossover from alisertib to the combination of alisertib and fulvestrant**					
	**Cycle 1, Day 1 of alisertib and fulvestrant, (if missed at time of progression on alisertib)**	**At the end of Cycle 1 of alisertib and fulvestrant**	**At time of disease progression on alisertib plus fulvestrant**	**Submit to:**	**Shipping/storage instructions**
**CTC**	One 10 mL blood sample collected in a CellSave® tube and one 10 mL blood sample collected in an AccuCyte tube	One 10 mL blood sample collected in a CellSave® tube and one 10 mL blood sample collected in an AccuCyte tube	One 10 mL blood sample collected in a CellSave® tube and one 10 mL blood sample collected in an AccuCyte tube	BAP freezer Mayo Clinic	Samples should be kept at ambient temperature after collection and during shipping Specimens must arrive at Mayo within 72 hours of collection
**cfDNA**	Two 10 mL blood samples collected in Streck® tubes	Two 10 mL blood samples collected in Streck® tubes	Two 10 mL blood samples collected in Streck® tubes	BAP freezer Mayo Clinic	Samples should be kept at ambient temperature after collection and during shipping Specimens must arrive at Mayo within 72 hours of collection

See Lab Manual for detailed instructions

Ship blood samples to: BAP Freezer
Stabile SL-16
Mayo Clinic
150 Third Street SW
Rochester, MN 55902
Telephone: 507-538-0602

Samples should be collected and shipped Monday – Thursday. However, if the subject can only be seen on Fridays, please ensure that Saturday delivery is marked clearly on the shipping boxes.

9.1.2 Methods

Circulating Tumor Cell Capture and Phenotyping

The relative abundance of CTCs in the peripheral blood has strong correlation with tumor recurrence and distant metastases in both early stage and advanced breast tumors[29]. We will prospectively isolate CTCs from blood specimens collected from study participants at three time points for phenotypic and possibly genotypic analyses.

One 10 mL blood sample will be collected in the CellSave® Preservation Tube for CTC isolation using the Veridex CellSearch™ system (Janssen; Raritan, NJ). CellSave® contains an optimized preservative that stabilizes cells for up to 96 hours at room temperature. The CellSearch™ system (http://veridex.com) allows for the

immunomagnetic selection, fluorescence staining, concentration, and enrichment of CTCs for enumeration and analysis. The FDA cleared process uses the CellSearch™ Epithelial Cell Kit, which contains anti-EpCAM (epithelial cell adhesion molecule) Ferrofluid, a fluorescent nucleic acid dye (DAPI), and fluorescently labeled monoclonal antibodies specific for leukocytes (CD45) and epithelial cells (cytokeratins 8, 18, and 19) to identify CTCs as those that are cytokeratin positive, DAPI positive, and CD45 negative with the appropriate morphology. At least two-thirds of patients with metastatic breast cancer have >1 CTC per 7.5 mL blood assayed with this technology, and the enumeration of CTCs relative to a threshold of 5 CTCs per 7.5 mL blood is associated with clinical outcomes in this patient population[27-29]. The standard process has been modified to allow for CTC retrieval and subsequent assessment for additional protein markers of interest (e.g., Aurora A kinase, ER, p-SMAD5, and p-SOX2).

CTCs vary in the degree of EpCAM expression, so malignant cells in the circulation may be missed by the CellSearch technology. Toward that end, other methodologies that do not rely on EpCAM for capture are now available. Therefore, a second 10 mL blood sample will be collected in the AccuCyte® Blood Collection Tube for CTC isolation using the AccuCyte® Platform (RareCyte; Seattle, WA). The blood collection tube contains an optimized preservative that stabilizes cells for up to 96 hours at ambient temperature. The AccuCyte® Platform (http://rarecyte.com) is a comprehensive, reproducible, and highly sensitive platform for enriching, identifying, and isolating CTCs for downstream molecular analyses (ref: Campton DE et al. BMC Cancer 2015. 15:360.). The system relies on density gradient centrifugation and allows for virtually complete harvesting of the buffy coat into a small volume for application to a microscopic slide without cell lysis or washing. Immunofluorescent staining for selected markers (e.g., cytokeratins, EpCAM, DAPI, CD45, HER2, ER, Aurora A kinase, p-SMAD5, and p-SOX2) is used for the efficient positive and negative identification of CTCs via automated scanning digital microscopy and image analysis. An integrated device provides for mechanically precise CTC retrieval for advanced genomic analyses, including next generation sequencing, on single or pooled CTCs.

9.2 Pathology Considerations/Tissue Biospecimens

9.2.1 Summary Table of Research Tissue Specimens to be Prospectively Collected for this Protocol

Note: Only send registration tissue if the patient qualifies for the study

Correlative Study (Section for more information)	Mandatory or Optional	Type of Tissue to Collect	Slides, Core, etc. (# of each to submit)	Pre-registration (biopsy)[1]	Upon completion of Cycle 1[2]	At Disease Progression	At disease progression after combination (ARM 1 ONLY)	Process at site?	Temperature Conditions for Storage /Shipping
Aurora A Kinase, SMAD5, SOX2 and ERα Expression	Mandatory	Formalin Fixed Paraffin Embedded	10 unstained slides (2 to 4 cores)	X	X	X	X	Yes	Ambient via Pathol coordinator
Real time PCR for ER target genes	Mandatory	Flash Frozen	1 core (3-4 mm)	X	X	X	X	Yes	Pathol coordinator
Patient Derived Xenograft	Mandatory for Mayo Clinic Subjects Only	Fresh	2 cores	X		X		Yes	In PBS or DMEM medium at room temperature within 30 minutes between the time when samples are obtained and the time samples are delivered to the xenograft team

Correlative Study (Section for more information)	Mandatory or Optional	Type of Tissue to Collect	Slides, Core, etc. (# of each to submit)	Pre-registration (biopsy)[1]	Upon completion of Cycle 1[2]	At Disease Progression	At disease progression after combination (ARM 1 ONLY)	Process at site?	Temperature Conditions for Storage /Shipping
LMW-E expression (MDACC)	Mandatory	Formalin Fixed Paraffin Embedded	1-2 unstained slides	X	X	X	X	Yes	MDACC Contact

1. If pre-registration biopsy is attempted and either aborted or insufficient tissue is obtained, the patient may still be eligible. In this case, the site may submit slides from archival specimens for central lab determination of ER status for patient stratification. Refer to Lab Manual for detailed instructions.
   NOTE: If biopsy does not obtain tissue AND no archived tissue is available for Central Laboratory review, patient is not eligible. A repeat biopsy may be attempted at the discretion of the treating physician and patient.
2. Performed once up to 4 days prior to the start of Cycle 2. Must be performed prior to initiation of treatment for Cycle 2.

Ship frozen tissue samples to:
BAP Freezer
Stabile SL-16
Mayo Clinic
150 Third Street SW
Rochester MN 55902

Phone: 507-538-0602

Ship tissue slides/cores to Mayo Clinic Cancer Center Operations Office
ATTN: PC Office (MC1431)
RO_FF_03_24-CC
Mayo Clinic
200 First Street SW
Rochester, MN 55905

9.2.2 Correlative Tumor Tissue Collection

In All Subjects:

9.2.2.1 Selection of metastatic lesion site for biopsy

A lung lesion should only be pursued for research biopsy in the event there is no other site of disease amenable to biopsy. If a lung lesion is the only site of disease amenable to biopsy, then peripheral lesions should be carefully selected due to risk of pneumothorax. Biopsy of central lesions is not advised. If a lung lesion is the only site of disease amenable to biopsy, then only 2 of the 3 mandatory biopsies will be required-- the pre-treatment/baseline and post-Cycle 1 biopsies. The biopsy at disease progression will be not be required, nor is it optional.

9.2.2.2 Two cores will be obtained with each research biopsy

One core will be formalin-fixed, paraffin-embedded (FFPE) and the tissue block will be sent to the Mayo Clinic Pathology Research Core (PRC). Sections (5 micron) will be cut from the tumor tissue block to obtain 10 unstained slides that contain at least 75% tumor tissue. These slides will be used for IHC assessment of the following biomarkers: Aurora A Kinase, SMAD5, SOX2 and ERα. One to two slides will be submitted to the laboratory of co-investigator, Dr. Khandan Keyomarsi (MDACC), for evaluation of both full length (nuclear) and LMW cyclin E (cytoplasmic) by IHC in FFPE tissue biospecimens.

One core will be flash frozen and sent to the Mayo Clinic PRC. RNA will be isolated from the biospecimen. Following cDNA synthesis, the expression levels of well characterized ERα target genes (TFF1, Progesterone Receptor, and amphiregulin) will be assessed by quantitative real-time PCR. Results will be expressed relative to an internal control housekeeping gene (TBP).

9.2.2.3 Aurora A Kinase, SMAD5, SOX2 and ERα Expression

Antibodies to detect total Aurora-A kinase, ERα, SMAD5 and SOX2, and phospho-SMAD5 and phospho-SOX2, are available through the Mayo PRC or will be purchased from Cell Signaling Technology, Boston, MA, USA and processed according to package insert. The phospho antibodies will be optimized through standard procedure with the PRC. Normal human breast tissues from reduction mammoplasty will serve as negative control and triple-negative breast tumor tissues as positive control. The intensity of biomarkers expression will be scored as strong, moderate, or weak-to-none using digital image analysis employing an innovative *ImageScope viewing software (Leica Biosystems)* to accurately measure Aurora-A, SMAD5, SOX2 and ERα nuclear expression in breast tumor clinical samples.

In Mayo Clinic Subjects Only:

9.2.2.4 Patient Derived Xenografts (PDX)

To generate individual patient derived xenografts (PDX), 2 additional research core specimens will be obtained during the baseline (pre-treatment) and progression of disease (PD) biopsies. The fresh tumor tissue will be immediately injected into 3 immunodeficient *scid* mice for the creation of human tissue xenografts (per IACUC A18014). The xenograft tumor samples will be used to evaluate mechanisms of de novo and acquired resistance to alisertib. These PDX models will be also be used for future research purposes, such as testing novel compounds and different regimens. All the tumor tissues from xenografts will be stored in the Mayo Clinic, Dr. Liewei Wang's laboratory Gonda 19 466E.

10. SPECIMEN BANKING

Any leftover study blood and tissue samples may be stored for future research studies. The subjects will consent to the future use of samples in the consent form for the study. Any samples will only be released for use in future studies after approval by the TBCRC and other regulatory bodies, as appropriate.

The study PI and collaborators have approval by the TBCRC to use all research bio-specimens collected during the conduct of this trial to address the research questions described in the protocol document. All future use of residual or repository specimens collected in this trial for purposes not prospectively defined will require review and approval by the TBCRC according to its established policies, whether the specimens are stored in a central site or at a local institution in a virtual repository.

Secondary use of bio-specimens for new endpoints must be submitted to the TBCRC Central Office for possible review by the TBCRC Correlative Science Review Committee.

11. STUDY CALENDAR

11.1 Test Schedule for Arm 1: Alisertib

			Alisertib Treatment Phase		***For patients who meet requirements set out in Section 6.1.2*[1]**<br>Alisertib + Fulvestrant Treatment Phase	
**Tests and procedures**	≤28 days prior to Pre-registration	Pre-registration period (spans the first 28 days after pre-registration)[2]	Day 1 of each treatment cycle*[3]	At PD or discontin-uation of alisertib	Day 1 of each treatment cycle[4]	At PD or discontinuation of alisertib + fulvestrant
History and exam, wt, PS	X		X[5]	X	X	X
Adverse event assessment		X[6]	X[6]	X	X	X
Height	X					
Follicle-stimulating hormone (FSH) level[7]		X				
Hematology group<br>– Hemoglobin<br>– White blood cell count<br>– Absolute neutrophil count<br>– Platelets		X	X[8]	X	X[9]	X

[1] For crossover registration procedure, see Section 5.4

[2] Hematology and chemistry blood tests and adverse event assessment for registration must be done ≤21 days prior to registration

[3]Labs, scans, tests and observations may be obtained ≤4 days prior to Day 1 of each cycle.

[4] Labs, H&E, wt, PS and AE assessment completed prior to switch to the combination of fulvestrant and alisertib may be used for Day 1 of "new" Cycle 1 tests if obtained ≤14 days prior to start of combination treatment.

[5] For Cycle 1, Day 1, nurse assessment may replace "History and exam, wt and PS" IF most recent physician/provider exam is ≤14 days prior to C1D1.

[6] Baseline AE assessment is to be performed anytime in the window of ≤4 days prior to registration. AE assessment need not be repeated Cycle 1 Day 1.

[7] For women <60 years of age with amenorrhea for >12 months

[8] Hematologic parameters will be assessed on Cycle 1 Day 15 (±1 day) after the patient has taken their morning dose of alisertib

[9] Hematologic parameters will be assessed on Day 15 (±1 day) of first cycle in the crossover phase any time after the patient has taken their morning dose of alisertib prior to the administration of the loading dose of fulvestrant.

			Alisertib Treatment Phase		***For patients who meet requirements set out in Section 6.1.2*[1]** Alisertib + Fulvestrant Treatment Phase	
**Tests and procedures**	≤28 days prior to Pre-registration	Pre-registration period (spans the first 28 days after pre-registration)[2]	Day 1 of each treatment cycle*[3]	At PD or discontin-uation of alisertib	Day 1 of each treatment cycle[4]	At PD or discontinuation of alisertib + fulvestrant
Chemistry group<br>– Sodium<br>– Potassium<br>– BUN<br>– Creatinine<br>– Calcium<br>– Glucose<br>– Albumin<br>– AST<br>– ALT[10]<br>– Alkaline Phosphatase<br>– Total Bilirubin[10]		X	X	X	X	X
Tumor measurement[11]	X		X	X	X[12]	X
Alisertib			X		X	
Fulvestrant					X[13]	
Patient Medication Diary (Appendix III)[14]			X	X	X	X

[10] FOR CYCLE 1 DAY 1 ONLY: If either to the following holds: (1) total bilirubin >1.5 x ULN or (2) Alanine transaminase >5 x ULN, then the patient should not begin protocol treatment. The patient should go off study and will be considered a cancel (see Section 6.4.8).

[11] The same imaging modality (CT scan, CT component of a PET/CT, or MRI) must be used throughout the study. Baseline imaging should be obtained ≤28 days prior to Registration. Tumor measurements are to be done at the completion of Cycles 2, 4, 6, etc., for first two years or until disease progression, whichever is first. Patients who have achieved complete response, partial response, or stable disease for ≥2 years may reduce imaging to every fourth cycle.

[12] Scans are not required Day 1 of Cycle 1 of fulvestrant and alisertib. The scans taken at the time of progression on alisertib will serve as the baseline scan for the fulvestrant and alisertib treatment phase of the study.

[13] Fulvestrant will be given on Day 1 of every cycle and on Day 15 (±1 day) of Cycle 1 only.

[14] The diary must begin the day the patient starts taking the medication and must be completed per protocol and returned to the treating institution at the completion of each cycle of treatment (approximately every 28 days). Compliance must be documented in the medical record by any member of the care team.

			Alisertib Treatment Phase		***For patients who meet requirements set out in Section 6.1.2*[1] Alisertib + Fulvestrant Treatment Phase	
**Tests and procedures**	≤28 days prior to Pre-registration	Pre-registration period (spans the first 28 days after pre-registration)[2]	Day 1 of each treatment cycle*[3]	At PD or discontin-uation of alisertib	Day 1 of each treatment cycle[4]	At PD or discontinuation of alisertib + fulvestrant
Mandatory tumor tissue specimens for research (see Section 9.0)		X[15]	X[16]	X[17]	X	X[18]
Research Correlative blood studies (collection for CTCs and cf-DNA)		X	X[19]	X	X[20]	X

* Cycle length is 28 days ±3 days

[15] The pre-registration biopsy will be utilized for central ER evaluation and correlative studies.
[16] Performed once up to 4 days prior to the start of Cycle 2. Must be performed prior to initiation of treatment for Cycle 2.
[17] Performed once up to 14 days after progressive disease is identified. Must be performed prior to initiation of next therapy, which may be the combination of fulvestrant and alisertib for those eligible and electing to crossover, or may be standard therapy for those who elect to come off study or are not eligible for cross-over. If a lung lesion is the only site of disease amenable to a biopsy, then this biopsy will not be mandatory (except for patients with ER <10% or ER negative on the pre-registration biopsy who are desiring and eligible for cross-over).
[18] Biopsy at PD after crossover is optional, except for patients with a lung lesion as the only site of disease amenable to biopsy in which case the biopsy at PD after crossover is not allowed.
[19] At the end of Cycle 1, prior to treatment initiation on Day 1 of Cycle 2 (only). May be drawn with clinical labs ≤4 days prior to C2D1.
[20] At the end of the first cycle of combination therapy, prior to treatment initiation on Day 1 of the second cycle of combination therapy (only).

11.2 Test Schedule for Arm 2: Alisertib + Fulvestrant

**Tests and procedures**	≤28 days prior to Pre-registration	Pre-registration period spans the first 28 days after pre-registration[21]	Day 1 of each treatment cycle[22]*	At PD or discontinuation of therapy
History and exam, wt, PS	X		X[23]	X
Adverse event assessment		X	X[24]	X
Height	X			
Follicle-stimulating hormone (FSH) level[25]		X		
Hematology group<br>– Hemoglobin<br>– White blood cell count<br>– Absolute Neutrophil count<br>– Platelets		X	X[26]	X
Chemistry group<br>– Sodium<br>– Potassium<br>– BUN<br>– Creatinine<br>– Calcium<br>– Glucose<br>– Albumin<br>– AST and ALT[27]<br>– Alkaline Phosphatase		X	X	X

[21] Hematology and chemistry blood tests and adverse event assessment for registration must be done ≤21 days prior to registration.
[22] Labs, scans, tests and observations may be obtained ≤4 days prior to Day 1 of each cycle.
[23] For Cycle 1, Day 1, nurse assessment may replace "History and exam, wt and PS" IF most recent physician/provider exam is ≤14 days prior to C1D1.
[24] Baseline AE assessment is to be performed anytime in the window of ≤4 days prior to registration. Adverse event assessment need not be repeated prior to Cycle 1, Day 1.
[25] For women <60 years of age with amenorrhea for >12 months.
[26] Hematologic parameters will be assessed on Day 15 of Cycle 1 any time after the patient has taken their morning dose of alisertib but before administration of fulvestrant.
[27] FOR CYCLE 1 DAY 1 ONLY: If either to the following holds: (1) total bilirubin >1.5 x ULN or (2) alanine transaminase >5 x ULN, then the patient should not begin protocol treatment. The patient should go off study and will be considered a cancel (see Section 6.4.8).

**Tests and procedures**	≤28 days prior to Pre-registration	Pre-registration period spans the first 28 days after pre-registration[21]	Day 1 of each treatment cycle[22]*	At PD or discontinuation of therapy
– Total Bilirubin[27]				
Tumor measurement[28]	X		X	X
Alisertib			X	
Fulvestrant			X[29]	
Patient Medication Diary (Appendix III)[30]			X	X
Mandatory tumor tissue specimens for research (see Section 9)		X[31]	X[32]	X[33]
Correlative blood studies (collection for CTCs and cf-DNA)		X	X[34]	X

* Cycle length is 28 ±3 days

[28] The same imaging modality (CT scan, CT component of a PET/CT, or MRI) must be used throughout the study. Baseline imaging should be obtained ≤28 days prior to Registration. Tumor measurements are to be done at the completion of Cycles 2, 4, 6 etc., for first two years or until disease progression, whichever is first. Patients who have achieved complete response, partial response, or stable disease for ≥2 years may reduce imaging to every fourth cycle.

[29] Fulvestrant will be given on Day 1 of every cycle and on Day 15 of Cycle 1 only.

[30] The diary must begin the day the patient starts taking the medication and must be completed per protocol and returned to the treating institution at the completion of each cycle of treatment (approximately every 28 days). Compliance must be documented in the medical record by any member of the care team.

[31] The pre-registration biopsy will be utilized for central ER evaluation and correlative studies.

[32] Biopsy must be performed ≤4 days prior to initiation of Cycle 2 treatment

[33] Performed once up to 14 days after progressive disease is identified. Must be performed prior to initiation of next therapy. If a lung lesion is the only site of disease amenable to rebiopsy, then this biopsy will not be mandatory.

[34] At the end of Cycle 1, prior to treatment initiation on Day 1 of Cycle 2 (only). May be drawn with clinical labs ≤4 days prior to C2D1

12. MEASUREMENT OF EFFECT

For the purposes of this study, patients should be re-evaluated for response after every 2 cycles of treatment.

NOTE: Patients who have achieved complete response, partial response, or stable disease for ≥2 years may reduce imaging to every fourth cycle.

Response and progression will be evaluated in this study using the international criteria proposed by the revised Response Evaluation Criteria in Solid Tumors (RECIST) Committee (version 1.1).(62) Changes in only the largest diameter (unidimensional measurement) of the tumor lesions are used in the RECIST criteria.

12.1 Definitions

Evaluable for toxicity: All patients will be evaluable for toxicity from the time of their first treatment with alisertib.

12.2 Disease Parameters

12.2.1 Measurable

12.2.1.1 A non-nodal lesion is considered measurable if its longest diameter can be accurately measured as ≥2.0 cm with chest x-ray, or as ≥1.0 cm with CT scan, CT component of a PET/CT, or MRI.

12.2.1.2 A superficial non-nodal lesion is measurable if its longest diameter is ≥1.0 cm in diameter as assessed using calipers (e.g. skin nodules) or imaging. In the case of skin lesions, documentation by color photography, including a ruler to estimate the size of the lesion, is recommended.

12.2.1.3 A malignant lymph node is considered measurable if its short axis is ≥1.5 cm when assessed by CT scan (CT scan slice thickness recommended to be no greater than 5 mm).

NOTE: Tumor lesions in a previously irradiated area are not considered measurable disease.

12.2.2 Non-measurable

All other lesions (or sites of disease) are considered non-measurable disease, including pathological nodes (those with a short axis ≥1.0 to <1.5 cm). Bone lesions, leptomeningeal disease, ascites, pleural/pericardial effusions, lymphangitis cutis/pulmonis, inflammatory breast disease, and abdominal masses (not followed by CT or MRI), are considered as non-measurable as well.

Note: 'Cystic lesions' thought to represent cystic metastases can be considered as measurable lesions, if they meet the definition of measurability described above. However, if non-cystic lesions are present in the same patient, these are preferred for selection as target lesions. In addition, lymph

nodes that have a short axis <1.0 cm are considered non-pathological (i.e., normal) and should not be recorded or followed.

12.2.3 Specifications by methods of measurements

12.2.3.1 Measurement of lesions

- All measurements should be recorded in metric notation (i.e., decimal fractions of centimeters) using a ruler or calipers.
- The same method of assessment and the same technique must be used to characterize each identified and reported lesion at baseline and during follow-up. For patients having only lesions measuring at least 1 cm to less than 2 cm must use CT imaging for both pre- and post-treatment tumor assessments.

12.2.4 Method of assessment

- Conventional CT and MRI: This guideline has defined measurability of lesions on CT scan based on the assumption that CT slice thickness is 5 mm or less. If CT scans have slice thickness greater than 5 mm, the minimum size for a measurable lesion should be twice the slice thickness.
- As with CT, if an MRI is performed, the technical specifications of the scanning sequences used should be optimized for the evaluation of the type and site of disease. The lesions should be measured on the same pulse sequence. Ideally, the same type of scanner should be used and the image acquisition protocol should be followed as closely as possible to prior scans. Body scans should be performed with breath-hold scanning techniques, if possible.
- PET-CT: If the site can document that the CT performed as part of a PET-CT is of identical diagnostic quality to a diagnostic CT (with IV and oral contrast), then the CT portion of the PET-CT can be used for RECIST measurements and can be used interchangeably with conventional CT in accurately measuring cancer lesions over time.
- Chest X-ray: Lesions on chest x-ray are acceptable as measurable lesions when they are clearly defined and surrounded by aerated lung. However, CT scans are preferable.
- FDG-PET: FDG-PET scanning is allowed to complement CT scanning in assessment of progressive disease [PD] and particularly possible 'new' disease. A ‘positive’ FDG-PET scanned lesion is defined as one which is FDG avid with an update greater than twice that of the surrounding tissue on the attenuation corrected image; otherwise, an FDG-PET scanned lesion is considered ‘negative.’ New lesions on the basis of FDG-PET imaging can be identified according to the following algorithm:

  a. Negative FDG-PET at baseline with a positive FDG-PET at follow-up is a sign of PD based on a new lesion.

  b. No FDG-PET at baseline and a positive FDG-PET at follow-up:

     i. If the positive FDG-PET at follow-up corresponds to a new site of disease confirmed by CT, this is PD.

ii. If the positive FDG-PET at follow-up is not confirmed as a new site of disease on CT at the same evaluation, additional follow-up CT scans (i.e., additional follow-up scans at least 4 weeks later) are needed to determine if there is truly progression occurring at that site. In this situation, the date of PD will be the date of the initial abnormal PDG-PET scan.

iii If the positive FDG-PET at follow-up corresponds to a pre-existing site of disease on CT that is not progressing on the basis of the anatomic images, it is not classified as PD.

12.2.5. Measurement at Follow-up Evaluation:

- The cytological confirmation of the neoplastic origin of any effusion that appears or worsens during treatment when the measurable tumor has met criteria for response or stable disease is mandatory to differentiate between response or stable disease (an effusion may be a side effect of the treatment) and progressive disease.
- Cytologic and histologic techniques can be used to differentiate between PR and CR in rare cases (e.g., residual lesions in tumor types such as germ cell tumors, where known residual benign tumors can remain.)

12.3 Response Criteria

12.3.1 Target lesions and target Lymph Nodes

- Measurable lesions (as defined in Section 12.2.1) up to a maximum of 5 lesions, representative of all involved organs, should be identified as “Target Lesions” and recorded and measured at baseline. These lesions can be non-nodal or nodal (as defined in 12.2.1), where no more than 2 lesions are from the same organ and no more than 2 malignant nodal lesions are selected.

  Note: If fewer than 5 target lesions and target lymph nodes are identified (as there often will be), there is no reason to perform additional studies beyond those specified in the protocol to discover new lesions.
- Target lesions and target lymph nodes should be selected on the basis of their size, be representative of all involved sites of disease, but in addition should be those that lend themselves to reproducible repeated measurements. It may be the case that, on occasion, the largest lesion (or malignant lymph node) does not lend itself to reproducible measurements in which circumstance the next largest lesion (or malignant lymph node) which can be measured reproducibly should be selected.
- Baseline Sum of Dimensions (BSD): A sum of the longest diameter for all target lesions plus the sum of the short axis of all the target lymph nodes will be calculated and reported as the baseline sum of

dimensions (BSD). The BSD will be used as reference to further characterize any objective tumor response in the measurable dimension of the disease.

- Post-Baseline Sum of the Dimensions (PBSD): A sum of the longest diameter for all target lesions plus the sum of the short axis of all the target lymph nodes will be calculated and reported as the post-baseline sum of dimensions (PBSD). If the radiologist is able to provide an actual measure for the target lesion (or target lymph node), that should be recorded, even if it is below 0.5 cm. If the target lesion (or target lymph node) is believed to be present and is faintly seen but too small to measure, a default value of 0.5 cm should be assigned. If it is the opinion of the radiologist that the target lesion or target lymph node has likely disappeared, the measurement should be recorded as 0 cm.
- The minimum sum of the dimensions (MSD) is the minimum of the BSD and the PBSD.

12.3.2. Evaluation of Target Lesions and Target Lymph Nodes

12.3.2.1 All target lesions and target lymph nodes followed by CT/MRI/PET-CT/Chest X-ray/ must be measured on re-evaluation. Specifically, a change in objective status to either a PR or CR cannot be done without re-measuring target lesions and target lymph nodes.

Note: Non-target lesions and non-target lymph nodes should be evaluated at each assessment, especially in the case of first response or confirmation of response. In selected circumstances, certain non-target organs may be evaluated less frequently. For example, bone scans may need to be repeated only when complete response is identified in target disease or when progression in bone is suspected.

12.3.2.2 Definitions

Complete Response (CR):	All of the following must be true:<br>a. Disappearance of all target lesions.<br>b. Each target lymph node must have reduction in short axis to <1.0 cm.
Partial Response (PR):	At least a 30% decrease in PBSD (sum of the longest diameter for all target lesions plus the sum of the short axis of all the target lymph nodes at current evaluation) taking as reference the BSD (*see* Section 12.3.1).
Progression (PD):	At least one of the following must be true:<br>a. At least one new malignant lesion, which also includes any lymph node that was normal at baseline (< 1.0 cm short axis) and increased to ≥ 1.0 cm short axis during follow-up.

b. At least a 20% increase in PBSD (sum of the longest diameter for all target lesions plus the sum of the short axis of all the target lymph nodes at current evaluation) taking as reference the MSD (Section 12.31). In addition, the PBSD must also demonstrate an absolute increase of at least 0.5 cm from the MSD.

c. See Section 12.2.4 for details in regards to the requirements for PD via FDG-PET imaging.

Stable Disease (SD): Neither sufficient shrinkage to qualify for PR, nor sufficient increase to qualify for PD taking as reference the MSD

12.3.3 Non-target lesions and Non-Target Lymph Nodes:

Non-measurable sites of disease (Section 12.2.2) are classified as non-target lesions or non-target lymph nodes and should also be recorded at baseline. These lesions and lymph nodes should be followed in accordance with 12.3.2.1.

12.3.3.1 Evaluation of Non-Target Lesions and Non-Target Lymph Nodes

12.3.3.2 Definitions

Complete Response (CR): All of the following must be true:

a. Disappearance of all non-target lesions

b. Each non-target lymph node must have a reduction in short axis to <1.0 cm.

Non-CR/Non-PD: Persistence of one or more non-target lesions or non-target lymph nodes.

Progression (PD): At least one of the following must be true:

a. At least one new malignant lesion, which also includes any lymph node that was normal at baseline (<1.0 cm short axis) and increased to ≥1.0 cm short axis during follow-up.

b. Unequivocal progression of existing non-target lesions and non-target lymph nodes. (NOTE: Unequivocal progression should not normally trump target lesion and target lymph node status. It must be representative of overall disease status change.)

c. See Section 12.2.4 for details in regards to the requirements for PD via FDG-PET imaging.

Table 3: Time point response

Target Lesions & Target Lymph Nodes	Non-Target Lesions & Non-Target Lymph Nodes	New Sites of Disease	Overall Objective Status
CR	CR	No	CR
CR	Non-CR/Non-PD	No	PR
PR	CR<br>Non-CR/Non-PD	No	PR
CR/PR	Not All Evaluated*	No	PR
SD	CR<br>Non-CR/Non-PD<br>Not All Evaluated*	No	SD
Not all Evaluated	CR<br>Non-CR/Non-PD<br>Not All Evaluated*	No	Not Evaluated (NE)
PD	Unequivocal PD<br>CR<br>Non-CR/Non-PD<br>Not All Evaluated*	Yes or No	PD
CR/PR/SD/PD/Not all Evaluated	Unequivocal PD	Yes or No	PD
CR/PR/SD/PD/Not all Evaluated	CR<br>Non-CR/Non-PD<br>Not All Evaluated*	Yes	PD

CR= complete response, PR= partial response, PD= progressive disease, SD= stable disease, NE= not evaluable.
*See Section 12.3.3

12.3.4 Symptomatic Deterioration

Patients with global deterioration of health status requiring discontinuation of treatment without objective evidence of disease progression at that time, and not either related to study treatment or other medical conditions, should be reported as PD due to "symptomatic deterioration." Every effort should be made to document the objective progression even after discontinuation of treatment due to symptomatic deterioration. A patient is classified as having PD due to "symptomatic deterioration" if any of the following occur that are not either related to study treatment or other medical conditions:

- Weight loss >10% of body weight.
- Worsening of tumor-related symptoms.
- Decline in performance status of >1 level on ECOG scale.

13. ADVERSE EVENT REPORTING REQUIREMENTS

The site principal investigator is responsible for reporting any/all serious adverse events to the sponsor as described within the protocol, regardless of attribution to study agent or treatment procedure.

The sponsor/sponsor-investigator is responsible for notifying FDA and all participating investigators in a written safety report of any of the following:

- Any suspected adverse reaction that is both serious and unexpected.
- Any findings from laboratory animal or *in vitro* testing that suggest a significant risk for human subjects, including reports of mutagenicity, teratogenicity, or carcinogenicity.
- Any findings from epidemiological studies, pooled analysis of multiple studies, or clinical studies, whether or not conducted under an IND and whether or not conducted by the sponsor, that suggest a significant risk in humans exposed to the drug.
- Any clinically important increase in the rate of a serious suspected adverse reaction over the rate stated in the protocol or Investigator's Brochure (IB).

Summary of Adverse Event Reporting for this Study

WHO:	WHAT form:	WHERE to send:
**All sites**	**Pregnancy Reporting** http://ctep.cancer.gov/protocolDevelopment/electronic_applications/docs/PregnancyReportFormUpdated.pdf	**Takeda:** TakedaOncoCases@cognizant.com<br>**Mayo Sites – attach to MCCC Electronic SAE Reporting Form** http://livecycle2.mayo.edu/workspace/?startEndpoint=MC4158-56/Processes/MC4158-56-Process.MC4158-56<br>**Non Mayo sites – complete and forward to** RSTP2CSAES@mayo.edu and TakedaOncoCases@cognizant.com
**Mayo Clinic Sites**	Mayo Clinic Cancer Center SAE Reporting Form: http://livecycle2.mayo.edu/workspace/?startEndpoint=MC4158-56/Processes/MC4158-56-Process.MC4158-56<br>AND attach<br>MedWatch 3500A: http://www.fda.gov/downloads/AboutFDA/ReportsManualsForms/Forms/UCM048334.pdf	**Takeda**: TakedaOncoCases@cognizant.com<br>Mayo CC AE Form will automatically be sent to<br>Mayo Clinic CC Regulatory and Compliance Office
**Non-Mayo Clinic Sites**	**MedWatch 3500A**: http://www.fda.gov/downloads/AboutFDA/ReportsManualsForms/Forms/UCM048334.pdf	**Takeda**: TakedaOncoCases@cognizant.com<br>**AND**<br>**Mayo Clinic CC:** RSTP2CSAES@mayo.edu

13.1 General

CTCAE term (AE description) and grade: The descriptions and grading scales found in the revised NCI Common Terminology Criteria for Adverse Events (CTCAE) version 4.0 will be utilized for AE reporting. All appropriate treatment areas should have access to a copy of the CTCAE version 4.0. A copy of the CTCAE version 4.0 can be downloaded from the CTEP website (http://ctep.cancer.gov/protocolDevelopment/electronic_applications/ctc.htm)

a. Identify the grade and severity of the event using the CTCAE version 4.0.
b. Determine whether the event is expected or unexpected (see Section 13.2.3).
c. Determine if the adverse event is related to the study intervention (agent, treatment or procedure) (see Section 13.2.4).
d. Determine whether the event must be reported as an expedited report. If yes, determine the timeframe/mechanism (see Section 13.3).
e. Determine if other reporting is required (see Section 13.4).
f. Note: All AEs reported via expedited mechanisms must also be reported via the routine data reporting mechanisms defined by the protocol (see Section 13.5).

NOTE: A severe AE, as defined by the grading scale below, is **NOT** the same as serious AE which is defined in the table in Section 13.2.

13.2 Definitions

Adverse Event
Adverse event (AE) means any untoward medical occurrence in a patient or subject administered a medicinal product; the untoward medical occurrence does not necessarily have a causal relationship with this treatment. An AE can therefore be any unfavorable and unintended sign (including an abnormal laboratory finding), symptom, or disease temporally associated with the use of a medicinal (investigational) product whether or not it is related to the medicinal product. This includes any newly occurring event, or a previous condition that has increased in severity or frequency since the administration of study drug.

An abnormal laboratory value will not be assessed as an AE unless that value leads to discontinuation or delay in treatment, dose modification, therapeutic intervention, or is considered by the investigator to be a clinically significant change from baseline.

Suspected Adverse Reaction
Serious AE (SAE) means any untoward medical occurrence that at any dose:

- Results in **death.**
- Is **life-threatening** (refers to an AE in which the patient was at risk of death at the time of the event. It does not refer to an event which hypothetically might have caused death if it were more severe).

- Requires inpatient **hospitalization or prolongation of an existing hospitalization** (see clarification in the paragraph below on planned hospitalizations).
- Results in **persistent or significant disability or incapacity**. (Disability is defined as a substantial disruption of a person's ability to conduct normal life functions).
- Is a **congenital anomaly/birth defect**.
- Is a **medically important event**. This refers to an AE that may not result in death, be immediately life threatening, or require hospitalization, but may be considered serious when, based on appropriate medical judgment, may jeopardize the patient, require medical or surgical intervention to prevent 1 of the outcomes listed above, or involves suspected transmission via a medicinal product of an infectious agent. Examples of such medical events include allergic bronchospasm requiring intensive treatment in an emergency room or at home, blood dyscrasias or convulsions that do not result in inpatient hospitalization, or the development of drug dependency or drug abuse; any organism, virus, or infectious particle (e.g., prion protein transmitting Transmissible Spongiform Encephalopathy), pathogenic or nonpathogenic, is considered an infectious agent.

Expedited Reporting
Events reported to sponsor within 24 hours, 5 days or 10 days of study team becoming aware of the event.

Routine Reporting
Events reported to sponsor via case report forms

Events of Interest
Events that would not typically be considered to meet the criteria for expedited reporting, but that for a specific protocol are being reported via expedited means in order to facilitate the review of safety data (may be requested by the FDA or the sponsor).

13.2.1 Expected vs. Unexpected Events

Expected events - are those described within the Section 8.0 of the protocol, the study specific consent form, package insert (if applicable), and/or the investigator brochure, (if an investigator brochure is not required, otherwise described in the general investigational plan).

Unexpected adverse events or suspected adverse reactions are those not listed in Section 8.0 of the protocol, the study specific consent form, package insert (if applicable), or in the investigator brochure (or are not listed at the specificity or severity that has been observed); if an investigator brochure is not required or available, is not consistent with the risk information described in the general investigational plan.

Unexpected also refers to adverse events or suspected adverse reactions that are mentioned in the investigator brochure as occurring with a class of drugs but have

not been observed with the drug under investigation.

An investigational agent/intervention might exacerbate the expected AEs associated with a commercial agent. Therefore, if an expected AE (for the commercial agent) occurs with a higher degree of severity or specificity, expedited reporting is required.

13.2.2 Attribution

Attribution is the relationship between an adverse event or serious adverse event and the study treatment. When assessing whether an adverse event (AE) is related to a medical agent(s) medical or procedure, the following attribution categories are utilized:

- Definite – The AE is clearly related to the study treatment.
- Probable – The AE is likely related to the study treatment.
- Possible – The AE may be related to the study treatment.
- Unlikely - The AE is doubtfully related to the study treatment.
- Unrelated - The AE is clearly NOT related to the study treatment.

Events determined to be possibly, probably or definitely attributed to a medical treatment suggest there is evidence to indicate a causal relationship between the drug and the adverse event.

13.3 Reporting Procedures

13.3.1 AEs Experienced Utilizing Investigational Agent(s) and Commercial Agent(s) during treatment with Alisertib alone

- An AE that occurs on an arm using an investigational agent /intervention under an IND/IDE must be assessed in accordance with the guidelines for investigational agents.
- An AE that occurs on an arm using a commercial agent on a separate treatment arm must be assessed as specified in the protocol**.**

Commercial agent expedited reports must be submitted to the FDA via MedWatch.3500A
http://www.fda.gov/downloads/AboutFDA/ReportsManualsForms/Forms/UCM048334.pdf
or
http://www.fda.gov/AboutFDA/ReportsManualsForms/Forms/ListFormsAlphabetically/default.htm

Instructions for completing the MedWatch 3500A:
http://www.fda.gov/downloads/Safety/MedWatch/HowToReport/DownloadForms/UCM387002.pdf

13.3.2 AEs Experienced Utilizing Investigational Agents and Commercial Agent(s) during treatment with the combination of fulvestrant and alisertib

NOTE: When a commercial agent(s) is (are) used on the same treatment arm as the investigational agent/intervention (also, investigational drug, biologic, cellular product, or other investigational therapy under an IND),

the **entire combination is then considered an investigational intervention for reporting.**

- An AE that occurs on a combination study must be assessed in accordance with the guidelines for **investigational** agents/interventions.
- An AE that occurs prior to administration of the investigational agent/ intervention must be assessed as specified in the protocol. In general, only Grade 4 and 5 AEs that are unexpected with at least possible attribution to the commercial agent require an expedited report, unless hospitalization is required. Refer to Section 10.4 for specific AE reporting requirements or exceptions.
- An investigational agent/intervention might exacerbate the expected AEs associated with a commercial agent. Therefore, if an expected AE (for the commercial agent) occurs with a higher degree of severity or specificity, expedited reporting is required.
- An increased incidence of an expected adverse event (AE) is based on the patients treated for this study at their site. A list of known/expected AEs is reported in the package insert or the literature, including AEs resulting from a drug overdose.
- Commercial agent expedited reports must be submitted to the FDA via MedWatch 3500A for Health Professionals (complete all three pages of the form).

  http://www.fda.gov/downloads/AboutFDA/ReportsManualsForms/Forms/UCM048334.pdf

  or

  http://www.fda.gov/AboutFDA/ReportsManualsForms/Forms/ListFormsAlphabetically/default.htm

  Instructions for completing the MedWatch 3500A:
  http://www.fda.gov/downloads/Safety/MedWatch/HowToReport/DownloadForms/UCM387002.pdf

13.3.3 Procedures for Reporting Serious Adverse Events

AEs may be spontaneously reported by the patient and/or in response to an open question from study personnel or revealed by observation, physical examination, or other diagnostic procedures. Any clinically relevant deterioration in laboratory assessments or other clinical finding is considered an AE. When possible, signs and symptoms indicating a common underlying pathology should be noted as one comprehensive event. AEs which are serious must be reported to Millennium Pharmacovigilance (or designee) from the first dose of <*alisertib*> up to and including <*30*> days after administration of the last dose of <*alisertib*>. Any SAE that occurs at any time after completion of <*alisertib*> treatment or after the designated follow-up period that the sponsor-investigator and/or sub-investigator considers to be related to any study drug must be reported to Millennium Pharmacovigilance (or designee). Planned hospital admissions or surgical

procedures for an illness or disease that existed before the patient was enrolled in the trial are not to be considered AEs unless the condition deteriorated in an unexpected manner during the trial (e.g., surgery was performed earlier or later than planned). All SAEs should be monitored until they are resolved or are clearly determined to be due to a patient's stable or chronic condition or intercurrent illness(es).

Since this is an investigator-initiated study, the principal investigator, Dr. Tufia Haddad, also referred to as the sponsor-investigator, is responsible for reporting serious adverse events (SAEs) to any regulatory agency and to the sponsor-investigator's EC or IRB.

Regardless of expectedness or causality, all SAEs must also be reported to Millennium Pharmacovigilance or designee:

Fatal and Life Threatening SAEs within 24 hours but no later than 4 calendar days of the sponsor-investigator's observation or awareness of the event

All other serious (non-fatal/non life threatening) events within 4 calendar days of the sponsor-investigator's observation or awareness of the event

The SAE report must include at minimum:

- **Event term(s)**
- **Serious criteria**
- **Intensity of the event(s):** Sponsor-investigator's or sub-investigator's determination. Intensity for each SAE, including any lab abnormalities, will be determined by using the NCI CTCAE version specified in the protocol, as a guideline, whenever possible. The criteria are available online at http://ctep.cancer.gov/reporting/ctc.html.
- **Causality of the event(s):** Sponsor-investigator's or sub-investigator's determination of the relationship of the event(s) to study drug administration.

Follow-up information on the SAE may be requested by Millennium.

In the event that this is a multisite study, the sponsor-investigator is responsible to ensure that the SAE reports are sent to Millennium Pharmacovigilance (or designee) from all sites participating in the study. Sub-investigators must report all SAEs to the sponsor-investigator so that the sponsor-investigator can meet his/her foregoing reporting obligations to the required regulatory agencies and to Millennium Pharmacovigilance, unless otherwise agreed between the sponsor-investigator and sub-investigator(s).

Relationship to all study drugs for each SAE will be determined by the investigator or sub-investigator by responding yes or no to the question: Is there a reasonable possibility that the AE is associated with the study drug(s)?

Sponsor-investigator must also provide Millennium Pharmacovigilance with a copy of all communications with applicable regulatory authorities related to the study product(s) as soon as possible but no later than 4 calendar days of such communication.

General Reporting instructions:

1. For all agents:
   Submit form Medwatch 3500A to the FDA, 5600 Fishers Lane, Rockville, MD 20852-9787, by fax at 1-800-332-0178 or online at http://www.fda.gov/Safety/MedWatch/HowToReport/default.htm.
2. Submit a copy of the completed MedWatch form and all other related forms to *CANCERCROSAFETYIN@mayo.edu*. For questions please see contact listed on page 3 of the protocol.
3. Send completed MedWatch form to Takeda/Millennium (see contact below) no later than 4 calendar days.

Mayo Clinic Cancer Center (MCCC) Institutions:

Use Mayo Clinic Cancer Center SAE Reporting Form: http://livecycle2.mayo.edu/workspace/?startEndpoint=MC4158-56/Processes/MC4158-56-Process.MC4158-56 to automatically submit to the following email address: CANCERCROSAFETYIN@mayo.edu. This email will be managed by the SAE, IND and Safety Reporting Coordinators.

Non-MCCC Institutions: Submit copies to RSTP2CSAES@mayo.edu AND *CANCERCROSAFETYIN@mayo.edu*.

**Millennium Pharmacovigilance or Designee**<br>**SAE and Pregnancy Reporting Contact Information**<br>**FAX Number 1-800-963-6290Email: TakedaOncoCases@cognizant.com**

13.3.4 Expedited Reporting Requirements for IND/IDE Agents

Phase 1 and Early Phase 2 Studies: Expedited Reporting Requirements for Adverse Events that Occur on Studies under an IND/IDE within 30 Days of the Last Administration of the Investigational Agent/Intervention [1, 2]

FDA REPORTING REQUIREMENTS FOR SERIOUS ADVERSE EVENTS (21 CFR Part 312)

NOTE: Investigators **MUST** immediately report to the sponsor **ANY** Serious Adverse Events, whether or not they are considered related to the investigational agent(s)/intervention (21 CFR 312.64)

An adverse event is considered serious if it results in **ANY** of the following outcomes:

1) Death
2) A life-threatening adverse event
3) An adverse event that results in inpatient hospitalization or prolongation of existing hospitalization for ≥ 24 hours
4) A persistent or significant incapacity or substantial disruption of the ability to conduct normal life functions
5) A congenital anomaly/birth defect.
6) Important Medical Events (IME) that may not result in death, be life threatening, or require hospitalization may be considered serious when, based upon medical judgment, they may jeopardize the patient or subject and may require medical or surgical intervention to prevent one of the outcomes listed in this definition. (FDA, 21 CFR 312.32; ICH E2A and ICH E6).

ALL SERIOUS adverse events that meet the above criteria MUST be immediately reported to the sponsor within the timeframes detailed in the table below.

Hospitalization	Grade 1 and Grade 2 Timeframes	Grade 3-5 Timeframes

Resulting in Hospitalization ≥24 hrs	7 Calendar Days	24-Hour 3 Calendar Days
Not resulting in Hospitalization ≥24 hrs	Not required	

Expedited AE reporting timelines are defined as:

- “24-Hour; 3 Calendar Days” - The AE must initially be reported within 24 hours of learning of the AE, followed by a complete expedited report within 3 calendar days of the initial 24-hour report.
- “7 Calendar Days” - A complete expedited report on the AE must be submitted within 7 calendar days of learning of the AE.

[1]Serious adverse events that occur more than 30 days after the last administration of investigational agent/intervention and have an attribution of possible, probable, or definite require reporting as follows:

Expedited 24-hour notification followed by complete report within 3 calendar days for:

- All Grade 3, 4, and Grade 5 AEs

Expedited 7 calendar day reports for:

- Grade 2 AEs resulting in hospitalization or prolongation of hospitalization

[2] For studies using PET or SPECT IND agents, the AE reporting period is limited to 10 radioactive half-lives, rounded UP to the nearest whole day, after the agent/intervention was last administered. Footnote “1” above applies after this reporting period.

Effective Date: May 5, 2011

Additional instructions:

The Mayo IND Coordinator will assist the sponsor-investigator in the processing of expedited adverse events and forwarding of suspected unexpected serious adverse reactions (SUSARs) to the FDA and IRB.

13.4 Other Required Reporting

13.4.1 Unanticipated Problems Involving Risks to Subjects or Others (UPIRTSOs)

UPIRTSOs in general, include any incident, experience, or outcome that meets **all** of the following criteria:

1. Unexpected (in terms of nature, severity, or frequency) given (a) the research procedures that are described in the protocol-related documents, such as the IRB-approved research protocol and informed consent document; and (b) the characteristics of the subject population being studied;
2. Related or possibly related to participation in the research (in this guidance document, possibly related means there is a reasonable possibility that the incident, experience, or outcome may have been caused by the procedures involved in the research); and
3. Suggests that the research places subjects or others at a greater risk of harm (including physical, psychological, economic, or social harm) than was previously known or recognized.

Some unanticipated problems involve social or economic harm instead of the physical or psychological harm associated with adverse events. In other cases,

unanticipated problems place subjects or others at increased *risk* of harm, but no harm occurs.

Note: If there is no language in the protocol indicating that pregnancy is not considered an adverse experience for this trial, and if the consent form does not indicate that subjects should not get pregnant/impregnate others, then any pregnancy in a subject/patient or a male patient's partner (spontaneously reported) which occurs during the study or within 120 days of completing the study should be reported as a UPIRTSO.

Mayo Clinic Cancer Center (MCCC) Institutions:
If the event meets the criteria for IRB submission as a Reportable Event/UPIRTSO, use Mayo Clinic Cancer Center SAE Reporting Form: http://livecycle2.mayo.edu/workspace/?startEndpoint=MC4158-56/Processes/MC4158-56-Process.MC4158-56 to automatically and provide the appropriate documentation automatically to CANCERCROSAFETYIN@mayo.edu. The Mayo Regulatory Affairs Office will review and process the submission to the Mayo Clinic IRB.

Non-MCCC Institutions*:*
Submit to your IRB as required by your institutional policies.
Submit copies to CANCERCROSAFETYIN@mayo.edu

13.4.2 Death

Note: A death on study requires both routine and expedited reporting regardless of causality, unless as noted below. Attribution to treatment or other cause must be provided.

Any death occurring within 30 days of the last dose, regardless of attribution to an agent/intervention under an IND/IDE requires expedited reporting within 24-hours.

Any death occurring greater than 30 days with an attribution of possible, probable, or definite to an agent/intervention under an IND/IDE requires expedited reporting within 24-hours.

Reportable categories of Death

- Death attributable to a CTCAE term.
- Death Neonatal: A disorder characterized by cessation of life during the first 28 days of life.
- Death NOS: A cessation of life that cannot be attributed to a CTCAE term associated with Grade 5.
- Sudden death NOS: A sudden (defined as instant or within one hour of the onset of symptoms) or an unobserved cessation of life that cannot be attributed to a CTCAE term associated with Grade 5.
- Death due to progressive disease should be reported as **Grade 5 "Neoplasms benign, malignant and unspecified (including cysts and polyps) – Other (Progressive Disease)"** under the system organ class

(SOC) of the same name. Evidence that the death was a manifestation of underlying disease (e.g., radiological changes suggesting tumor growth or progression: clinical deterioration associated with a disease process) should be submitted.

13.4.3 Secondary Malignancy

- A ***secondary malignancy*** is a cancer caused by treatment for a previous malignancy (e.g., treatment with investigational agent/intervention, radiation or chemotherapy). A secondary malignancy is not considered a metastasis of the initial neoplasm.
- All secondary malignancies that occur following treatment with an agent under an IND/IDE will be reported. Three options are available to describe the event:
  - Leukemia secondary to oncology chemotherapy (e.g., Acute Myelocytic Leukemia [AML])
  - Myelodysplastic syndrome (MDS)
  - Treatment-related secondary malignancy
- Any malignancy possibly related to cancer treatment (including AML/MDS) should also be reported via the routine reporting mechanisms outlined in each protocol.

13.4.4 Second Malignancy

A second malignancy is one unrelated to the treatment of a prior malignancy (and is NOT a metastasis from the initial malignancy). Second malignancies require ONLY routine reporting unless otherwise specified.

13.4.5 Pregnancy

Procedures for Reporting Drug Exposure during Pregnancy and Birth Events

If a woman becomes pregnant or suspects that she is pregnant while participating in this study, she must inform the investigator immediately and permanently discontinue study drug. The sponsor-investigator must fax a completed Pregnancy Form to the Millennium Department of Pharmacovigilance or designee. The pregnancy must be followed for the final pregnancy outcome.

If a female partner of a male patient becomes pregnant during the male patient's participation in this study, the sponsor-investigator must also immediately fax a completed Pregnancy Form to the Millennium Department of Pharmacovigilance or designee. Every effort should be made to follow the pregnancy for the final pregnancy outcome.

13.4.6 Product Complaints

A product complaint is a verbal, written, or electronic expression that implies dissatisfaction regarding the identity, strength, purity, quality, or stability of a drug product. Individuals who identify a potential product complaint situation should immediately contact MedComm Solutions (see below) and report the

event. Whenever possible, the associated product should be maintained in accordance with the label instructions pending further guidance from a Millennium Quality representative.

For Product Complaints
1-844-ONC-TKDA (1-844-662-8532)
(US and International)
E-mail: ***GlobalOncologyMedinfo@takeda.com***

Product complaints in and of themselves are not AEs. If a product complaint results in an SAE, an SAE form should be completed and sent to Millennium Pharmacovigilance (refer to Section 8.2).

A product complaint is a verbal, written, or electronic expression that implies dissatisfaction regarding the identity, strength, purity, quality, or stability of a drug product. Individuals who identify a potential product complaint situation should immediately contact MedComm Solutions (see below) and report the event. Whenever possible, the associated product should be maintained in accordance with the label instructions pending further guidance from a Millennium Quality representative.

A medication error is a preventable event that involves an identifiable patient and that leads to inappropriate medication use, which may result in patient harm. While overdoses and underdoses constitute medication errors, doses missed inadvertently by a patient do not. Individuals who identify a potential medication error situation should immediately contact MedComm Solutions (see below) and report the event.

13.5 Required Routine Reporting

Pretreatment symptoms/conditions to be graded at baseline and adverse events to be graded at each evaluation per CTCAE v4.0 grading **unless** otherwise stated in the table below:

CTCAE System Organ Class (SOC)	Adverse event/Symptoms	Baseline	Each evaluation
Blood and Lymphatic System Disorders	Anemia	X	X
Gastrointestinal Disorders	# of stools	X	
	Diarrhea		X
	Nausea	X	X
	Vomiting	X	X
	Mucositis Oral	X	X
General disorders and administration site conditions	Fatigue	X	X
	Injection site reaction		X
Investigations	Platelet Count Decreased	X	X
	Neutrophil Count Decreased	X	X
	White Blood Cell Decreased	X	X

CTCAE System Organ Class (SOC)	Adverse event/Symptoms	Baseline	Each evaluation
Metabolism and nutrition disorders	Anorexia	X	X
Renal and urinary disorders	Acute Kidney Injury	X	X
Skin and subcutaneous tissues	Rash acneiform	X	X
	Rash maculo-papular	X	X

13.5.1 Submit via appropriate MCCC Case Report Forms (i.e., paper or electronic, as applicable) the following AEs experienced by a patient and not specified in Section 13.5:

13.5.1.1 Grade 2 AEs deemed *possibly, probably, or definitely* related to the study treatment or procedure.

13.5.1.2 Grade 3 and 4 AEs regardless of attribution to the study treatment or procedure.

13.5.1.3 Grade 5 AEs (Deaths)

13.5.1.3.1 Any death within 30 days of the patient's last study treatment or procedure regardless of attribution to the study treatment or procedure.

13.5.1.3.2 Any death more than 30 days after the patient's last study treatment or procedure that is felt to be at least possibly treatment related must also be submitted as a Grade 5 AE, with a CTCAE type and attribution assigned.

13.5.2 Late Occurring Adverse Events

Refer to the instructions in the Forms Packet (or electronic data entry screens, as applicable) regarding the submission of late occurring AEs following completion of the Active Monitoring Phase (i.e., compliance with Test Schedule in Section 11).

14. DATA AND SAFETY MONITORING

14.1 Data Management and Reporting

Submission Timetables

14.1.1 Pre-Registration Material(s)

Case Report Form (CRF)	Active-Monitoring Phase (Compliance with Test Schedule Section 4.0)
Pre-Registration Screening Failure	Complete only if patient is NOT registered after she is pre-registered
Pathology materials	See Section 17.0 (for central review, eligibility, etc. confirmation prior to Registration)

14.1.2 Initial Material(s)

CRF	Active-Monitoring Phase (Compliance with Test Schedule Section 11)
Pathology Materials (including local pathology report)	See Section 9 (for central review, eligibility, etc. confirmation prior to Registration/Randomization)
On-Study Form	≤2 weeks after registration
Adverse Events: Baseline	
Institutional Contact	
On-Study: Prior Disease History (Left Breast) as applicable	
On-Study: Prior Disease History (Right Breast) as applicable	
On-Study: Prior Neoadjuvant or Adjuvant Therapy	
On-Study: Prior Systemic Therapy for Metastatic Disease	
Metastatic Disease Status	
RECIST Measurement: Baseline	
Supporting Documentation: Baseline	
Specimen Submission: Tissue (see Section 9)	
Specimen Submission: Blood	
Patient Status: Baseline	
Off Treatment	Submit ≤2 weeks after registration if withdrawal/ refusal occurs prior to beginning protocol therapy

14.1.3 Test Schedule Material(s)

CRF	At each evaluation during treatment	At end of treatment
Treatment (Intervention)	X[1]	X
Treatment (Intervention): Dose Modifications, Omission and Delays[2]	X	X
Adverse Event: Solicited	X	X
RECIST Measurements	X	X
RECIST Measurements: Cross-over Baseline[3] – Arm 1 patients that cross-over only	X	
RECIST Measurements: Cross-over Arm 1 patients that cross-over only	X	X
Specimen Submission: Tissue	X (see Section 9)	
Specimen Submission: Blood	X (see Section 9)	
Supporting Documentation	X	X
Patient Status: Treatment (Intervention)	X	X
End of Initial Treatment[2] – Arm 1 patients that cross-over only		X
Off Treatment		X
Consent Withdrawal (choose appropriate withdrawal form): Specimen Only, All Follow-Up[2]	If applicable	
Lost to Follow-Up[2]	If applicable	
ADR/AER	At each occurrence (see Section 13)	

1. Complete at each evaluation during Active Treatment (see Section 11).
2. Complete only if applicable
3. At initial cross-over for Arm 1 patients only

14.1.4 Follow-up Material(s)

CRF	Event Monitoring Phase[1]		
	Every 6 months until Death	Death	New Primary
Patient Status: Survival and Disease Status Follow-Up/Event Monitoring	X	X	
Consent Withdrawal (choose appropriate withdrawal form): Specimen Only, All Follow-Up[2]	X		
Lost to Follow-Up[2]	X		
Notice of New Primary[2]			At each occurrence

1. If a patient is still alive 5 years after registration, no further follow-up is required.
2. Complete only if applicable

14.2 All paper forms should be sent to:

MC1431
Operations Office
Mayo Clinic Cancer Center
NW Clinic 3rd Floor
200 First Street SW
Rochester, MN 55905

14.3 Labeling

Each co-sponsor/participant will be responsible for insuring that all materials contain the patient's initials, MCCC registration number, and MCCC protocol number. Patient's name must be removed.

14.4 Incomplete materials

Any materials deemed incomplete by the MCCC Operations Office will be considered "not received" and will not be edited or otherwise processed until the missing information is received. A list of the missing documents will be made available to the appropriate co-sponsor/participant.

14.5 Overdue lists

A list of overdue materials and forms for study patients will be generated monthly. The listings will be sorted by location and will include the patient study registration number. The appropriate co-sponsor/participant will be responsible to obtain the overdue material.

14.6 Corrections forms

If a correction is necessary the QAS will query the co-sponsor/participant. The query will be sent to the appropriate co-sponsor/participant who will make the correction and return the query and documentation of correction back to the QAS.

14.7 External Monitoring

External monitoring of participating sites will occur at pre-determined time points. A data monitoring plan will be generated by a contract research organization. This document will be independent of the protocol. This document will be provided to all study sites for their submission to their local scientific review committees and IRBs as their particular requirements dictate.

15. REGULATORY CONSIDERATIONS

15.1 Protocol Review and Amendments

Information regarding study conduct and progress will be reported to the Institutional Review Board (IRB) per the current institutional standards of each participating center.

Any changes to the protocol will be made in the form of an amendment and must be approved by the IRB of each institution prior to implementation.

The Protocol Chair (or his designee) is responsible for the coordination and development of all protocol amendments, and will disseminate this information to the participating centers.

15.2 Informed Consent

The investigator (or his/her designee) will explain to each subject the nature of the study, its purpose, the procedures involved, the expected duration, the potential risks and benefits involved and any discomfort it may entail. Each subject will be informed that participation in the study is voluntary, that s/he may withdraw from the study at any time, and that withdrawal of consent will not affect her subsequent medical treatment or relationship with the treating physician(s) or institution. The informed consent will be given by means of a standard written statement, written in non-technical language, which will be IRB approved. The subject should read and consider the statement before signing and dating it, and will be given a copy of the document. No subject will enter the study or have study-specific procedures done before his/her informed consent has been obtained.

In accordance with the Health Information Portability and Accountability Act (HIPAA), the written informed consent document (or a separate document to be given in conjunction with the consent document) will include a subject authorization to release medical information to the study sponsor and supporting agencies and/or allow these bodies, a regulatory authority, or Institutional Review Board access to subjects' medical information that includes all hospital records relevant to the study, including subjects' medical history.

15.3 Ethics and GCP

This study will be carried out in compliance with the protocol and Good Clinical Practice, as described in:

1. ICH Harmonized Tripartite Guidelines for Good Clinical Practice 1996.
2. US 21 Code of Federal Regulations dealing with clinical studies (including parts 50 and 56 concerning informed consent and IRB regulations).
3. Declaration of Helsinki, concerning medical research in humans (Recommendations Guiding Physicians in Biomedical Research Involving Human Subjects, Helsinki 1964, amended Tokyo 1975, Venice 1983, Hong Kong 1989, Somerset West 1996).

The investigator agrees to adhere to the instructions and procedures described in it and thereby to adhere to the principles of Good Clinical Practice that it conforms to.

16. MULTI-CENTER GUIDELINES

16.1 Study Documentation

Each participating site is responsible for submitting copies of all relevant regulatory documentation to the Coordinating Center. The required documents include, but are not limited to the following: local IRB approvals (i.e., protocol, consent form, amendments, patient brochures and recruitment material, etc.), IRB membership rosters, summary of unanticipated problems or protocol deviations, and documentation of expertise of the investigators. The Coordinating Center will provide each participating site with a comprehensive list of the necessary documents. It is the responsibility of the participating sites to maintain copies of all documentation submitted to the Coordinating Center.

16.2 Records Retention

Following closure of the study, each participating center will maintain a copy of all site study records in a safe and secure location.

16.3 Publication

It is understood that any manuscript or releases resulting from the collaborative research will be circulated to all participating sites prior to submission for publication or presentation.

17. STATISTICAL CONSIDERATIONS AND METHODOLOGY

The aims of this clinical trial include assessing the impact of adding fulvestrant to alisertib in terms of clinical benefit (response rate and progression-free survival) and safety.

Among the patients who are assigned to alisertib monotherapy (Arm 1) who progress and choose to receive the combination of fulvestrant and alisertib, the primary aims are to examine response and the differences in time to progression with alisertib and that with fulvestrant and alisertib.

Initial treatment refers to alisertib alone for those randomized to Arm 1 and alisertib with fulvestrant for those randomized to Arm 2. Crossover treatment only applies to patients who progress during treatment with alisertib alone on Arm 1.

17.1 Assessing the impact of adding fulvestrant to alisertib

17.1.1 Primary Endpoint

The primary endpoint is tumor response rate with initial treatment. That is,

- Arm 1: Tumor response rate is defined as 100% times the number of patients who meet the criteria for CR or PR (using RECIST criteria version 1.1) on 2 consecutive evaluations approximately 8 weeks apart during treatment with alisertib monotherapy divided by the number of patients who started alisertib monotherapy.
- Arm 2: Tumor response rate is defined as 100% times the number of patients who meet the criteria for CR or PR (using RECIST criteria version 1.1) on 2 consecutive evaluations approximately 8 weeks apart

during treatment with combination of alisertib and fulvestrant divided by the number of patients who started treatment with the combination of alisertib and fulvestrant.

The study will test the hypothesis that the addition of fulvestrant to alisertib will increase the tumor response rate over that of alisertib alone by at least 20%.

17.1.2 Secondary Endpoints

17.1.2.1 Duration of response during initial treatment- time from randomization to disease progression among those patients whose disease meets the RECIST criteria for CR or PR on 2 consecutive evaluations approximately 8 weeks apart during initial treatment.

17.1.2.2 Clinical Benefit Rate (CBR) during initial treatment - the proportion of patients who have completed 6 cycles of treatment without disease progression (that is, their objective disease status is a CR, PR, or stable for 6 cycles or more) during initial treatment.

17.1.2.3 Progression-free survival (PFS) - time from randomization to disease progression or death due to any cause. If a patient has not had a disease evaluation within 3 months of death, the patient will be censored at the time of last disease evaluation for estimating the distribution of PFS time.

17.1.2.4 Safety Profile: The CTCAE version 4.0 will be used to grade and assign attribution to each adverse event reported during initial treatment and crossover treatment separately.

17.2 Sample Size/ Accrual Rate

Melichar et al reported the results of a Phase II clinical trial of alisertib (administered at 50 mg BID for 7 days of a 21 day cycle) monotherapy in women with chemo-resistant metastatic breast cancer[24]. The response rate among the 14 women with triple negative breast cancer was 7.1 % (median PFS: 1.5 months). The response rate among the 26 women with hormone receptor positive disease was 23.1% (median PFS: 7.9 months). Given these results and the expectation that approximately 10% of the patients who will be enrolled onto this trial will have triple negative breast cancer, we anticipate the response rate with alisertib alone will be 20%.

A randomized phase II clinical trial design (proposed by Jung et al[30]) which incorporates a prospective control treatment and a futility stopping rule was chosen to assess whether the tumor response rate with the addition of fulvestrant to alisertib will be greater than that of alisertib alone by at least 20%, when the tumor response rate for alisertib alone is assumed to be 20%. The randomized phase II clinical trial design with a prospective control group proposed by Jung takes into account the interim analysis for futility so that overall design parameters are met.

With a sample size of 45 patients per arm, a one sided alpha=0.15 sequential binomial test of the difference in two independent proportions will have a 85% chance of detecting that the response rate with the addition of fulvestrant to alisertib is at least 20% or greater

than the response rate with alisertib alone, when the true response rate for alisertib alone is 20% or less. The null hypothesis that the difference in tumor responses is less than 20% would be rejected if at least 4 more tumor responses are seen with the combination of fulvestrant and alisertib than with alisertib alone.

It is anticipated that none of the patients who are pre-registered will fail to obtain an ER result from the biopsy of a metastatic lesion. However, some patient may cancel participation prior to registration/randomization or after randomization but prior to the start of assigned treatment and other patients may be found to be ineligible. These patients will be replaced. It is anticipated that an additional 6 patients may be enrolled to account for these situations.

Futility stopping rule: If, after the first 28 patients on each treatment arm have been enrolled and completed at least 6 cycles of treatment, the difference in the number of tumor responses between the treatment arms is one or fewer, enrollment will be halted and the DSMB will be consulted to formulate patient treatment recommendations.

Other Considerations that might lead to early closure: Toxicity, patterns of treatment failure observed in this study and scientific discoveries or changes in standard care will be taken into account in any recommendation to the DSMB to terminate this trial earlier than anticipated.

17.2.1 Crossover: Patients who are assigned to alisertib only arm who progress may choose to receive the combination of fulvestrant and alisertib. We anticipate that at least 14 (if there is no signal that combination therapy may be more beneficial) and as many as 22 patients will choose to receive combination therapy. As such, a 95% binomial confidence interval will be constructed for both the tumor response rate and clinical benefit rate during crossover treatment.

17.3 Stratification Factors

This study will use the Pocock and Simon dynamic allocation procedure[30] to allocate an equal number of patients to each of the treatment strategies. This procedure will balance the marginal distributions of the stratification factors, clinical endocrine resistance and ER status, between these treatment strategies.

- Clinical Resistance to Endocrine Therapy:
  1= Primary endocrine resistance- recurrence within the first 2 years of adjuvant endocrine therapy, or progression within the first 6 months of initiating first-line endocrine therapy for MBC
  2= Secondary endocrine resistance- recurrence during Years 2-5 of adjuvant endocrine therapy (or within 12 months of completing adjuvant endocrine therapy) or progression occurring 6 or more months after initiating endocrine therapy for MBC.
- ER findings from registration biopsy
  1 = ER positive (≥10% staining)
  2 = ER weakly positive (1 to 9.9% staining) or ER negative (0% staining)
- Prior therapy with a CDK 4/6 inhibitor (palbociclib, ribociclib or abemaciclib)
  1=yes

2=no

17.4 Analysis of Secondary Endpoints

17.4.1 For each treatment arm, the distribution of PFS times, overall survival times, and duration of response will be estimated using the Kaplan-Meier method.

17.4.2 Clinical benefit rate

For initial treatment in each arm, the clinical benefit rate at 24 weeks will be defined as the proportion of patients who completed 6 cycles of treatment without documentation of disease progression. A 90% confidence interval for the CBR will be constructed using the Duffy-Santner approach to take into account the sequential nature of the study design.

17.4.3 Examination of baseline and 4 week on initial treatment tumor biopsy specimens

Spearman rank correlation coefficient will be used to examine the association between ERα expression and the biomarkers: CD44, CD24, total and phosphorylated expression of AURKA, SMAD5, and SOX2 at baseline. Assuming that 90% of the 90 patients enrolled onto this trial will have sufficient baseline tissue to complete these analyses; there will be 81 patients will data available for these analyses. With a sample size of 81, a two-sided alpha=0.05 test of Spearman rank correlation coefficient will have a 90% chance of rejecting the true correlation is 0 when the true correlation is 35% or more.

A two sample test of the difference in proportions will be used to examine whether weak or no phosphorylated expression of AURKA, SMAD5, and SOX2 after one cycle of alisertib is associated with clinical benefit (CR +PR + SD on treatment for at least 6 months). The clinical response rate in ER+ MBC with single agent alisertib was reported to be 54%. As such we will assume that approximately 50% of the patients enrolled onto each treatment arm will have a documented clinical response. If, in a given treatment arm, 20 patients have a clinical response and 20 patients do not, a two sided alpha=0.05 test of the difference in two independent proportions will have at least an 85% chance of detecting a difference of 45% between the percentage of patients with a no or little expression of the biomarker among those who had clinical benefit and the percentage of patients with a no or little expression of the biomarker among those who did not have clinical benefit when the true percentage of patients with a no or little expression of the biomarker among those who did not have clinical benefit is 10% (or 20% or 25%).

17.4.4 Examination of changes in tumor biomarkers after one cycle of initial treatment

For each treatment arm, a Spearman rank correlation coefficient will be used to examine the association of maximum percentage of tumor shrinkage during treatment with the percent change after 1 cycle of treatment in AAK expressing cells, as well as percent changes after 1 cycle of treatment in tissue ERα, SMAD5, SOX2 expression and phosphorylation.

17.4.5 Examination of changes in blood biomarkers after one cycle of initial treatment

At the end of Cycle 1, the percent change in CTC expression of Aurora A kinase, ER, and phospho- SOX2 expression from pre-treatment levels will be determined for each patient.

Bland-Altman plots and weighted kappa statistics will be used to examine the concordance between the percent change in Aurora A kinase, ER, and phospho-SOX2 expression from pre-treatment levels in CTC and in tumor tissue.

A plot of the percent change expression of Aurora A kinase, ER, and phospho-SOX2 expression from pre-treatment levels in CTCs and that in tissue will be constructed with using different symbols for each type of clinical response (CR, PR, SD or PD) to visually assess for trends.

17.5 Monitoring

17.5.1 Study Monitoring

The study chair and faculty statistician will review the trial data every 3 months to identify accrual, toxicity, and endpoint problems that might be developing. The faculty statistician will prepare a report containing accrual, adverse events, and efficacy data which will be submitted to the MCCC Data and Safety Monitoring Board (DSMB) on a semi-annual basis.

17.5.2 Safety Stopping Rule

Pre-Amendment 3: Safety stopping rules will be applied to each treatment arm separately. If 3 or more of the first 10 patients randomized to a given treatment arm or 30% of more of patients randomized to that treatment arm thereafter develops a grade 4 hematologic or non-hematologic toxicity possibly, probably or definitely related to treatment, the enrollment to the trial will be temporarily suspended so that all AE data can be examined. The study chair and the faculty statistician will formulate a trial recommendation to present to the IRB for approval. These instances will also be reported to the MCCC DSMB.

Post-Amendment 3: This safety stopping rule will be applied to each treatment arm separately for the patients enrolled after the activation of Amendment 3. If 2 or more of the first 6 patients randomized to a given treatment arm or 30% of more of patients randomized to that treatment arm thereafter develops a Grade 4 hematologic or non-hematologic toxicity possibly, probably or definitely related to treatment, the enrollment to the trial will be temporarily suspended so that all AE data can be examined. The study chair and the faculty statistician will formulate a trial recommendation to present to the IRB for approval. These instances will also be reported to the MCCC DSMB.

17.6 Inclusion of Women and Minorities

This study will be available to all eligible patients regardless of race or ethnic group. The expected number of patients per racial/ethnicity categories are presented in the following table. The sample size for this trial was not increase in order to provide additional power for analyses by race or ethnicity.

Accrual Targets			
	Sex/Gender		
**Ethnic Category**	**Females**	**Males**	**Total**
Hispanic or Latino	7		7
Not Hispanic or Latino	89		89
**Ethnic Category: Total of all subjects**	96		96
**Racial Category**			
American Indian or Alaskan Native	1		1
Asian	6		6
Black or African American	7		7
Native Hawaiian or other Pacific Islander	0		0
White	82		82
**Racial Category: Total of all subjects**	96	0	96

Ethnic Categories: **Hispanic or Latino –** a person of Cuban, Mexican, Puerto Rican, South or Central American, or other Spanish culture or origin, regardless of race. The term "Spanish origin" can also be used in addition to "Hispanic or Latino."

Not Hispanic or Latino

Racial Categories: **American Indian or Alaskan Native –** a person having origins in any of the original peoples of North, Central, or South America, and who maintains tribal affiliations or community attachment.

Asian – a person having origins in any of the original peoples of the Far East, Southeast Asia, or the Indian subcontinent including, for example, Cambodia, China, India, Japan, Korea, Malaysia, Pakistan, the Philippine Islands, Thailand, and Vietnam. (Note: Individuals from the Philippine Islands have been recorded as Pacific Islanders in previous data collection strategies.)

Black or African American – a person having origins in any of the black racial groups of Africa. Terms such as "Haitian" or "Negro" can be used in addition to "Black or African American."

Native Hawaiian or other Pacific Islander – a person having origins in any of the original peoples of Hawaii, Guam, Samoa, or other Pacific Islands.

White – a person having origins in any of the original peoples of Europe, the Middle East, or North Africa.

18. BUDGET

18.1 Costs charged to patient: Routine clinical care

18.2 Treatment and tests to be research funded: Alisertib, research blood work and tumor biopsies at pre-registration, at completion of Cycle 1, and at disease progression.

REFERENCES

1. Osborne CK, Schiff R: Mechanisms of endocrine resistance in breast cancer. Ann Rev Med 2011, 62:233-247
2. Ring A, Dowsett M: Mechanisms of tamoxifen resistance. Endocr Relat Cancer 2004, 11:643-658
3. Ali S, Coombes RC: Endocrine-responsive breast cancer and strategies for combating resistance. Nature reviews Cancer 2002, 2:101-112
4. Dodwell D, Wardley A, Johnston S: Postmenopausal advanced breast cancer: options for therapy after tamoxifen and aromatase inhibitors. Breast 2006, 15:584-594
5. Schiff R, Massarweh SA, Shou J, Bharwani L, Mohsin SK, Osborne CK: Cross-talk between estrogen receptor and growth factor pathways as a molecular target for overcoming endocrine resistance. Clin Cancer Res 2004, 10:331S-336S
6. Pece S, Tosoni D, Confalonieri S, Mazzarol G, Vecchi M, Ronzoni S, Bernard L, Viale G, Pelicci PG, Di Fiore PP: Biological and molecular heterogeneity of breast cancers correlates with their cancer stem cell content. Cell 2010, 140:62-73
7. Mani SA, Guo W, Liao MJ, Eaton EN, Ayyanan A, Zhou AY, Brooks M, Reinhard F, Zhang CC, Shipitsin M, Campbell LL, Polyak K, Brisken C, Yang J, Weinberg RA: The epithelial-mesenchymal transition generates cells with properties of stem cells. Cell 2008, 133:704-715
8. Hwang-Verslues WW, Kuo WH, Chang PH, Pan CC, Wang HH, Tsai ST, Jeng YM, Shew JY, Kung JT, Chen CH, Lee EY, Chang KJ, Lee WH: Multiple lineages of human breast cancer stem/progenitor cells identified by profiling with stem cell markers. PloS ONE 2009, 4:e8377
9. Shipitsin M, Campbell LL, Argani P, Weremowicz S, Bloushtain-Qimron N, Yao J, Nikolskaya T, Serebryiskaya T, Beroukhim R, Hu M, Halushka MK, Sukumar S, Parker LM, Anderson KS, Harris LN, Garber JE, Richardson AL, Schnitt SJ, Nikolsky Y, Gelman RS, Polyak K: Molecular definition of breast tumor heterogeneity. Cancer Cell 2007, 11:259-273
10. Horwitz KB, Dye WW, Harrell JC, Kabos P, Sartorius CA: Rare steroid receptor-negative basal-like tumorigenic cells in luminal subtype human breast cancer xenografts. Proc Natl Acad Sci U S A 2008, 105:5774-5779
11. Bhola NE, Balko JM, Dugger TC, Kuba MG, Sanchez V, Sanders M, Stanford J, Cook RS, Arteaga CL: TGF-beta inhibition enhances chemotherapy action against triple-negative breast cancer. J Clin Invest 2013, 123:1348-1358
12. Ithimakin S, Day KC, Malik F, Zen Q, Dawsey SJ, Bersano-Begey TF, Quraishi AA, Ignatoski KW, Daignault S, Davis A, Hall CL, Palanisamy N, Heath AN, Tawakkol N, Luther TK, Clouthier SG, Chadwick WA, Day ML, Kleer CG, Thomas DG, Hayes DF, Korkaya H, Wicha MS: HER2 drives luminal breast cancer stem cells in the absence of HER2 amplification: implications for efficacy of adjuvant trastuzumab. Cancer Res 2013, 73:1635-1646
13. Chang CJ, Yang JY, Xia W, Chen CT, Xie X, Chao CH, Woodward WA, Hsu JM, Hortobagyi GN, Hung MC: EZH2 promotes expansion of breast tumor initiating cells through activation of RAF1-beta-catenin signaling. Cancer Cell 2011, 19:86-100
14. Balboni AL, Hutchinson JA, DeCastro AJ, Cherukuri P, Liby K, Sporn MB, Schwartz GN, Wells WA, Sempere LF, Yu PB, DiRenzo J: DeltaNp63alpha-mediated activation of bone morphogenetic protein signaling governs stem cell activity and plasticity in normal and malignant mammary epithelial cells. Cancer Res 2013, 73:1020-1030
15. Siggelkow W, Boehm D, Gebhard S, Battista M, Sicking I, Lebrecht A, Solbach C, Hellwig B, Rahnenfuhrer J, Koelbl H, Gehrmann M, Marchan R, Cadenas C, Hengstler JG, Schmidt M: Expression of aurora kinase A is associated with metastasis-free survival in node-negative breast cancer patients. BMC Cancer 2012, 12:562
16. Creighton CJ, Xiaoxian L, Landis M, Dixon JM, Neumeister VM, Sjolund A, Rimm DL, Wong H, Rodriguez A, Herschkowitz JI, Fan C, Zhang X, He X, Pavlick A, Gutierrez C, Renshaw L, Larionov AA, Faratian D, Hilsenbeck SG, Perou CM, Lewis MT, Rosen JM, Chang JC: Residual

breast cancers after conventional therapy display mesenchymal as well as tumor-initiating features. PNAS 2009, 106:13820-13825

17. D'Assoro AB, Liu T, Quatraro C, Amato A, Opyrchal M, Leontovich A, Ikeda Y, Ohmine S, Lingle W, Suman V, Ecsedy J, Iankov I, Di Leonardo A, Ayers-Inglers J, Degnim A, Billadeau D, McCubrey J, Ingle J, Salisbury JL, Galanis E: The mitotic kinase Aurora--a promotes distant metastases by inducing epithelial-to-mesenchymal transition in ERalpha(+) breast cancer cells. Oncogene 2014, 33:599-610
18. Opyrchal M, Salisbury JL, Zhang S, McCubrey J, Hawse J, Goetz MG, Lomberk GA, Haddad T, Degnim A, Lange C, Ingle JN, Galanis E, D'Assoro AB: Aurora-A Mitotic Kinase Induces Endocrine Resistance through Down-Regulation of ER-alpha Expression in Initially ER+ Breast Cancer Cells. PLoS ONE 2014, 9:e96995
19. Thrane S, Pedersen A, Thomsen M, al e: A kinase inhibitor screen identifies Mcl-1 and Aurora kinase A as novel treatment targets in antiestrogen-resistant breast cancer cells. Oncogene 2014, Online publication November 3:
20. Lindstrom LS, Karlsson E, Wilking UM, Johansson U, Hartman J, Lidbrink EK, Hatschek T, Skoog L, Bergh J: Clinically used breast cancer markers such as estrogen receptor, progesterone receptor, and human epidermal growth factor receptor 2 are unstable throughout tumor progression. J Clin Oncol 2012, 30:2601-2608
21. Friedberg JW, Mahadevan D, Cebula E, Persky D, Lossos I, Agarwal AB, Jung J, Burack R, Zhou X, Leonard EJ, Fingert H, Danaee H, Bernstein SH: Phase II study of alisertib, a selective Aurora A kinase inhibitor, in relapsed and refractory aggressive B- and T-cell non-Hodgkin lymphomas. J Clin Oncol 2014, 32:44-50
22. Dees EC, Cohen RB, von Mehren M, Stinchcombe TE, Liu H, Venkatakrishnan K, Manfredi M, Fingert H, Burris HA, 3rd, Infante JR: Phase I study of aurora A kinase inhibitor MLN8237 in advanced solid tumors: safety, pharmacokinetics, pharmacodynamics, and bioavailability of two oral formulations. Clin Cancer Res 2012, 18:4775-4784
23. Huck JJ, Zhang M, Mettetal J, Chakravarty A, Venkatakrishnan K, Zhou X, Kleinfield R, Hyer ML, Kannan K, Shinde V, Dorner A, Manfredi MG, Shyu WC, Ecsedy JA: Translational exposure-efficacy modeling to optimize the dose and schedule of taxanes combined with the investigational Aurora A kinase inhibitor MLN8237 (alisertib). Mol Cancer Ther 2014, 13:2170-2183
24. Melichar B, DeMichele A, Denis A, Bourbouloux E, Tan-Chiu E, Niu H, Schusterbauer C, Dansky Ullmann C, Zhang B, Benaim E: Phase 2 study of single agent MLN8237 (alisertib), an investigational aurora A kinase (AAK) inhibitor, in patients with relapsed/refractory breast cancer. Lancet Oncol. 2015 Apr;16(4):395-405:
25. Di Leo A, Jerusalem G, Petruzelka L, Torres R, Bondarenko IN, Khasanov R, Verhoeven D, Pedrini JL, Smirnova I, Lichinitser MR, Pendergrass K, Garnett S, Lindemann JP, Sapunar F, Martin M: Results of the CONFIRM phase III trial comparing fulvestrant 250 mg with fulvestrant 500 mg in postmenopausal women with estrogen receptor-positive advanced breast cancer. J Clin Oncol 2010, 28:4594-4600
27. Cristofanilli M, Budd GT, Ellis MJ, Stopeck A, Matera J, Miller MC, Reuben JM, Doyle GV, Allard WJ, Terstappen LW, Hayes DF: Circulating tumor cells, disease progression, and survival in metastatic breast cancer, N Engl J Med 2004, 351:781-791
28. Hayes DF, Cristofanilli M, Budd GT, Ellis MJ, Stopeck A, Miller MC, Matera J, Allard WJ, Doyle GV, Terstappen LW: Circulating tumor cells at each follow-up time point during therapy of metastatic breast cancer patients predict progression-free and overall survival, Clin Cancer Res 2006, 12:4218-4224
29. Liu MC. By the numbers: does circulating tumor cell enumeration have a role in metastatic breast cancer? J Clin Oncol. 2014;32(31):3479-82

30. Pocock SJ, Simon R: Sequential treatment assignment with balancing for prognostic factors in the controlled clinical trial. Biometrics 1975, 31:103-115
31. SH Jung: Randomized phase II trials with a prospective control. Stat Med 2008, 27:568-583

APPENDICES

Appendix I – ECOG Performance Status and Karnofsky Performance Status

PERFORMANCE STATUS CRITERIA<br>Please use ECOG Score for this protocol			
**ECOG (Zubrod)**		**Karnofsky**	
**Score**	**Description**	**Score**	**Description**
**0**	**Fully active; able to carry on all pre-disease performance without restriction**	100	Normal: no complaints, no evidence of disease
		90	Able to carry on normal activity; minor symptoms of disease
**1**	**Restricted in physically strenuous activity but ambulatory and able to carry out work of a light or sedentary nature, e.g., light housework, office work**	80	Normal activity with effort; some signs or symptoms of disease
		70	Cares for self; unable to carry on normal activities or do active work
**2**	**Ambulatory and capable of all self care but unable to carry out any work activities; up and about more than 50% of waking hours**	60	Requires occasional assistance; cares for most of his/her needs
		50	Requires considerable assistance and frequent medical care
**3**	**Capable of only limited self care; confined to bed or chair more than 50% of waking hours**	40	Disabled: requires special care and assistance
		30	Severely disabled: hospitalized but death not imminent
**4**	**Completely disabled; cannot carry on any self care; totally confined to bed or chair**	20	Very sick: active supportive care needed but death not imminent
		10	Moribund: fatal processes are progressing rapidly

Karnofsky PS from *Karnofsky DA, Abelmann WH, Craver LF, Burchenal JH: The use of the nitrogen mustards in the palliative treatment of carcinoma. Cancer 1, 634-656, 1948*.

*ECOG As published in Am. J. Clin. Oncol.: *Oken, M.M., Creech, R.H., Tormey, D.C., Horton, J., Davis, T.E., McFadden, E.T., Carbone, P.P.: Toxicity And Response Criteria Of The Eastern Cooperative Oncology Group. Am J Clin Oncol 5:649-655, 1982.*

The ECOG Performance Status is in the public domain therefore available for public use. To duplicate the scale, please cite the reference above and credit the Eastern Cooperative Oncology Group, Robert Comis M.D., Group Chair. From http://www.ecog.org/general/perf_stat.html

Appendix II – NYHA Heart Failure Classification

NYHA Class	Symptoms
I	Cardiac disease, but no symptoms and no limitation in ordinary physical activity, e.g. no shortness of breath when walking, climbing stairs etc.
II	Mild symptoms (mild shortness of breath and/or angina) and slight limitation during ordinary activity.
III	Marked limitation in activity due to symptoms, even during less-than-ordinary activity, e.g. walking short distances (20–100 m).<br>Comfortable only at rest.
IV	Severe limitations. Experiences symptoms even while *at rest*. Mostly bedbound patients.

Adapted from Dolgin M, Association NYH, Fox AC, Gorlin R, Levin RI, New York Heart Association. Criteria Committee. Nomenclature and criteria for diagnosis of diseases of the heart and great vessels. 9th ed. Boston, MA: Lippincott Williams and Wilkins; March 1, 1994.
Original source: Criteria Committee, New York Heart Association , Inc. Diseases of the Heart and Blood Vessels. Nomenclature and Criteria for diagnosis, 6th edition Boston, Little, Brown and Co. 1964, p 114.

Appendix III – Medication Diary

Initials (optional)________________________ Patient Study ID___________

Study Name/Number MC1431/TBCRC041 Cycle___________

Diary Instructions

- Please bring your Medication Diary and any empty or unused medication container(s) with you to every appointment.
- Please use an ink pen when completing the Medication Diary as these will be retained in our research record.
- To correct an error or mistake, please make a single line through that entry and write your initials and date next to the error or mistake.
- Please record each dose as soon as you take it and fill in the date as directed. Please also record the number of tablets you took for each dose of alisertib.
- Please indicate on the calendar below every day that you take your study medication by placing the time dose was taken on the line under the date.
- If you forget to take a dose and the next scheduled dose is to be taken in less than 6 hours, do not take the missed dose. If you miss a dose, DO NOT MAKE UP THIS DOSE ON A DIFFERENT DAY. Write the 'Date' in the appropriate place, leave the 'Time' blank, and write '0' for number of tablets taken for this missed dose.

Drug Instructions

- Alisertib should be taken regardless of the timing of meals. You should take your Alisertib with about 250 mL (1 cup) of water.
- Your two doses of alisertib should be taken every 12 hours. These doses need to be taken at least 6 hours apart.
- If your cycle starts in PM, you will take the PM dose only on Day 1 and an AM dose on Day 4.
- Do not take proton pump inhibitors (e.g., omeprazole, lansoprazole, pantoprazole), H2 antagonists (e.g., famotidine, ranitidine, cimetidine) or pancreatic enzymes.
- Do not take any neutralizing antacids or calcium-containing supplements 2 hours prior to taking alisertib and wait to take these until 2 hours after taking your alisertib.
- Store alisertib at room temperature
- If you vomit after taking alisertib, do not take another dose. Wait until your next scheduled dose before taking alisertib.

Other Instructions

- Please contact your physician or study coordinator any time you go into the hospital. Your physician can advise if you should stop taking your medication or continue it.

Study Medications	Dose
Alisertib (oral)	*MG*

	Day 1		***Day 2***		***Day 3***		***Day 4***		***Days 5-7***
Date:									
Time of Morning Dose<br>Number of pills taken	*AM*	*# of pills*	*AM*	*# of pills*	AM	*# of pills*	*AM*	*# of pills*	*NO PILLS AM*
Time of Afternoon Dose<br>Number of pills taken	*PM*	*# of pills*	*PM*	*# of pills*	*PM*	*# of pills*	*NO PILLS PM*		*NO PILLS PM*

	Day 8		***Day 9***		***Day 10***		***Days 11-14***
Date:							
Time of Morning Dose<br>Number of pills taken	*AM*	*# of pills*	*AM*	*# of pills*	AM	*# of pills*	*NO PILLS AM*
Time of Afternoon Dose<br>Number of pills taken	*PM*	*# of pills*	*PM*	*# of pills*	*PM*	*# of pills*	*NO PILLS PM*

	Day 15		***Day 16***		***Day 17***		***Days 18-28***
Date:							
Time of Morning Dose<br>Number of pills taken	*AM*	*# of pills*	*AM*	*# of pills*	AM	*# of pills*	*NO PILLS AM*
Time of Afternoon Dose<br>Number of pills taken	*PM*	*# of pills*	*PM*	*# of pills*	*PM*	*# of pills*	*NO PILLS PM*

NOTE: *Day 18 through Day 28 you will not take any drug*

*Participant Signature*_______________________________ *Date:* _______________

Area Below To Be Completed By Study Staff Only

Number of Pills returned: __________ Study Coordinator Initials: ______________
Date: _____________________ Discrepancy: Yes_________ No ________

Appendix IV – Proton Pump Inhibitor (PPI) and H2 antagonist

Alisertib has low and pH-dependent solubility. It is an enteric coated tablet designed to bypass the acidic gastric pH. Per a recently completed DDI study with esomeprazole, co-administration with PPIs resulted in ~30% increase in alisertib systemic exposure. For this reason chronic use of PPI or H2 antagonist medications is not allowed.

Proton Pump Inhibitors

- omeprazole (Prilosec)
- lansoprazole (Prevacid)
- rabeprazole (Aciphex)
- pantoprazole (Protonix)
- esomeprazole (Nexium)

H2- Blockers

- ranitidine (Zantac)
- cimetidine (Tagamet)
- famotidine (Pepcid)
- nizatidine (Axid)
