# Supplementary material for: Evaluation of Alisertib Alone or Combined With Fulvestrant in Patients With Endocrine-Resistant Advanced Breast Cancer: The Phase 2 TBCRC041 Randomized Clinical Trial
Source: JAMA Oncol. 2023 Mar 9;9(6):815–24. doi: 10.1001/jamaoncol.2022.7949 (PMC9999287; doi:10.1001/jamaoncol.2022.7949)
Supplement: Supplement 2. — eTable 1. Dose levels and modifications for alisertib eTable 2. Safety stopping rule event summary and approved protocol changes [file jamaoncol-e227949-s002.pdf]

Supplemental Online Content

Haddad TC, Suman VJ, D'Assoro AB, et al. Evaluation of alisertib alone or combined with fulvestrant in patients with endocrine-resistant advanced breast cancer: the phase 2 TBCRC041 randomized clinical trial. *JAMA Oncol*. Published online March 9, 2023.
doi:10.1001/jamaoncol.2022.7949

eTable 1. Dose levels and modifications for alisertib

eTable 2. Safety stopping rule event summary and approved protocol changes

This supplemental material has been provided by the authors to give readers additional information about their work.

© 2023 Haddad TC et al. *JAMA Oncology.*

eTable 1: Dose levels and modifications for alisertib

Dose for alisertib and modifications for adverse events are described. Starting dose (Dose level 0) of alisertib is 50mg oral twice daily on days 1-3, 8-10, 15-17 of a 28-day cycle. Dose level -1 is 40mg and dose level -2 is 30mg.

CTCAE System/ Organ/ Class	ADVERSE EVENT	Dose modifications – prior to Day 1 of each cycle ACTION
Blood and lymphatic system disorders	Anemia Grade 3 with hemoglobin <7.0 g/dL or Grade 4	First or Second Episode: Hold alisertib administration until recovery to ≤Grade 2 or baseline then decrease by 1 dose level
		If not recovered within 14 days discontinue alisertib and proceed to event monitoring phase*
		Third Episode: Discontinue study treatment (see note*).
		*NOTE: If patient is deriving benefit from study treatment and event can be managed safely, consult Study Chair regarding continuation.
Investigations	Neutrophil Count Decreased Grade 3 (neutrophils <1000 - 500 cells/mm^3)	First Episode: Hold alisertib administration and check neutrophil counts within 5-7 days of report of Grade 3 ANC (referred to as ANC recheck #1).
		(a) results of ANC recheck #1
		▪ If neutrophils ≥1500 cells/mm^3, resume alisertib at current dose
		▪ If neutrophils <1500 cells/mm^3, continue to hold alisertib and re-check neutrophils counts again within the next 7 days but at most 14 days after the first report of Grade 3 ANC (ANC recheck #2)
		(b) results of ANC recheck #2
		▪ If neutrophils ≥1500 cells/mm^3, resume alisertib at one dose lower
		▪ If neutrophils remain <1500 cells/mm^3 14 days after first report of Grade 3 ANC , discontinue study treatment*
		Second Episode: Hold alisertib administration and check neutrophil count within 5-7 days of report of Grade 3 ANC (referred to as ANC recheck #1).
		(a) results of ANC recheck #1
		▪ If neutrophils ≥1500 cells/mm^3, resume alisertib at first dose level below current dose level
		▪ If neutrophils <1500 cells/mm^3, continue to hold alisertib and re-check neutrophils counts again within the next 7 days but at most 14 days after the first report of Grade 3 ANC (ANC recheck #2)
		(b) results of ANC recheck #2

© 2023 Haddad TC et al. *JAMA Oncology.*

CTCAE System/ Organ/ Class	ADVERSE EVENT	Dose modifications – prior to Day 1 of each cycle ACTION
		▪ If neutrophils ≥1500 cells/mm^3, resume alisertib at first dose level below current dose
		▪ If neutrophils remain <1500 cells/mm^3 14 days after first report of Grade 3 ANC in this second episode, discontinue study treatment*
		Third Episode: discontinue study treatment (see note*)
		*NOTE: If patient is deriving benefit from study treatment and event can be managed safely, consult Study Chair regarding continuation.
Investigations	Neutrophil count decreased Grade 4 (neutrophils <500 cells/mm^3)	First Episode: Hold alisertib administration and check neutrophil counts within 5-7 days of report of Grade 4 ANC (referred to as ANC recheck #1).
		(a) results of ANC recheck #1
		▪ If neutrophils ≥1500 cells/mm^3, resume alisertib at first dose level below at current dose
		▪ If neutrophils <1500 cells/mm^3, continue to hold alisertib and re-check neutrophils counts again within the next 7 days but at most 14 days after the first report of Grade 4 ANC (ANC recheck #2)
		(b) results of ANC recheck #2
		▪ If neutrophils ≥1500 cells/mm^3, resume alisertib at first dose level below at current dose
		▪ If neutrophils remain <1500 cells/mm^3 14 days after first report of Grade 4 ANC, discontinue study treatment*
		Second Episode: Hold alisertib administration and check neutrophil counts within 5-7 days of report of Grade 4 ANC results of ANC recheck #1
		(a) results of ANC recheck #1
		▪ If neutrophils ≥1500 cells/mm^3, resume alisertib at first dose level below current dose
		▪ If neutrophils <1500 cells/mm^3, continue to hold alisertib and re-check neutrophils counts again within the next 7 days but at most 14 days after the first report of Grade 4 ANC (ANC recheck #2)
		(b) results of ANC recheck #2
		▪ If neutrophils ≥1500 cells/mm^3, resume alisertib at first dose level below at current dose
		▪ If neutrophils remain <1500 cells/mm^3 14 days after first report of Grade 4 ANC , discontinue study treatment*
		Third Event: Discontinue study treatment (see note*)

© 2023 Haddad TC et al. *JAMA Oncology.*

CTCAE System/ Organ/ Class	ADVERSE EVENT	Dose modifications – prior to Day 1 of each cycle ACTION
		*NOTE: If patient is deriving benefit from study treatment and event can be managed safely, consult Study Chair regarding continuation.
Investigations	Platelet count decreased Grade 1 and 2 (<100,000 cells/mm^3 but >50,000 cells/mm^3)	Hold alisertib administration and check platelet counts every 3-5 days until platelets ≥100,000 cells/mm^3 or for a maximum of 21 days after Grade 1-2 decrease in PLT reported
		If platelet count recovered to ≥100,000 cells/mm^3 within 21 days then resume alisertib at current dose
		Otherwise discontinue study treatment *
		*NOTE: If patient is deriving benefit from study treatment and event can be managed safely, consult Study Chair regarding continuation
Investigations	Platelet count decreased Grade 3 (25,000 – 50,000 cells/mm^3)	First Episode: Hold alisertib administration and check neutrophil counts within 5-7 days of report of Grade 3 PLT (referred to as PLT recheck #1)
		(a) results of PLT recheck #1
		▪ If platelets ≥100,000 cells/mm^3, resume alisertib at current dose
		▪ If platelets <100,000 cells/mm^3, continue to hold alisertib and re-check platelet count again within the next 5- 7 days but at most 14 days after the first report of Grade 3 ANC (ANC recheck #2)
		(b) results of PLT recheck #2
		▪ If platelets ≥100,000 cells/mm^3, resume alisertib at first dose level below at current dose
		▪ If platelets remains <100,000 cells/mm^3 14 days after first report of Grade 3 PLT decrease, discontinue study treatment
		Second Episode: Hold alisertib administration and check platelet counts within 5-7 days of report of Grade 1 ANC results of ANC recheck #1
		(a) results of PLT recheck #1
		▪ If platelets ≥100,000 cells/mm^3, resume alisertib at current dose
		▪ If platelets <100,000 cells/mm^3, continue to hold alisertib and re-check platelet counts again within the next 7 days but at most 14 days after the first report of Grade 3 PLT (PLT recheck #2)
		(b) results of PLT recheck #2
		▪ If platelets ≥100,000 cells/mm^3, resume alisertib at first dose level below at current dose

© 2023 Haddad TC et al. *JAMA Oncology.*

CTCAE System/ Organ/ Class	ADVERSE EVENT	Dose modifications – prior to Day 1 of each cycle ACTION
		▪ If platelets remains <100,000 cells/mm^3 14 days after first report of Grade 3 PLT decrease, discontinued 14 days after first report of Grade 3 ANC, discontinue study treatment
		Third Event: Discontinue all study treatment (see note*).
		*NOTE: If patient is deriving benefit from study treatment and event can be managed safely, consult Study Chair regarding continuation
	Platelet count decreased Grade 4 (<25,000/mm^3) or Grade 3 with bleeding	Discontinue all study treatment
		*NOTE: If patient is deriving benefit from study treatment and event can be managed safely, consult Study Chair regarding continuation.
Blood and lymphatic system disorders	Febrile neutropenia Grade 3 (neutrophils <1000 cells/mm^3 with a single temperature of >38.3°C (101°F) or a sustained temperature of ≥38°C (100.4°F) for more than one hour.	Discontinue all study treatment*
		*NOTE: If patient is deriving benefit from study treatment and event can be managed safely, consult Study Chair regarding continuation.
	Grade 4 (Life threatening consequences)	
Gastrointestinal disorders	Mucositis oral Grade 3 or 4	First event:
		Hold alisertib administration until recovered to ≤Grade 1 or pre-treatment level
		If recovered Grade 1 or baseline within 21 days, continue alisertib one dose level below current dose level
		Second event:
		Hold alisertib administration until recovered to ≤Grade 1 or pre-treatment level
		If recovered to ≤Grade 1 or baseline within 21 days, continue alisertib one dose level below current dose level
		If not, discontinue all study treatment*
		Third event: Discontinue all study treatment (see note*)
		*NOTE: If patient is deriving benefit from study treatment and event can be managed safely, consult Study Chair regarding continuation.
Nervous system disorders	Somnolence ≥Grade 3	Hold alisertib administration until recovered to ≤Grade 1 or pre-treatment level

© 2023 Haddad TC et al. *JAMA Oncology.*

CTCAE System/ Organ/ Class	ADVERSE EVENT	Dose modifications – prior to Day 1 of each cycle ACTION
		If recovered to ≤Grade 1 or baseline within 14 days, continue alisertib one dose level below current dose level
		If not, discontinue all study treatment (see note*).
		NOTE: Evaluate whether patient alcohol consumption or utilization of benzodiazepines are contributing. If so, counsel patient to restrict alcohol and discontinue benzodiazepines with provider guidance
		*NOTE: If patient is deriving benefit from study treatment and event can be managed safely, consult Study Chair regarding continuation
Other unspecified adverse event reduction instructions	≥Grade 3	Hold alisertib administration until recovered to ≤Grade 1 or pre-treatment level (baseline level)
		If recovered to ≤Grade 1 or baseline within 14 days, continue alisertib one dose level below current dose level
		If not, discontinue all study treatment *
		*NOTE: If patient is deriving benefit from study treatment and event can be managed safely, consult Study Chair regarding continuation
**CTCAE System/ Organ/ Class**	**ADVERSE EVENT**	**Dose modifications – prior to Cycle 1 Day 15 only ACTION**
Investigations	Neutrophil count decreased Grade 3 or 4 (neutrophils <1000 cells/mm^3)	Omit Cycle 1 Days 15-17 administration of alisertib
		Administer fulvestrant loading dose
		Check neutrophil count on Cycle 2 Day 1

© 2023 Haddad TC et al. *JAMA Oncology.*

eTable 2: Safety stopping rule event summary and approved protocol changes

The safety stopping boundary was crossed after 12 patients had received at least one cycle of treatment. Five patients had developed a Grade 4 toxicity or died on treatment. A summary of AEs and attribution to alisertib is provided along with a summary of the protocol changes in response to these events.

Patient	Arm	Adverse Event Summary	Attribution
1	A	Death due to cardiac arrest and grade 4 thrombotic microangiopathic hemolysis (cycle 4)	Death is unrelated (due to PD); hemolysis possibly due to alisertib
2	A	Death due to acute respiratory failure (initial manifestation of acute coronary syndrome) during cycle 3; underlying lung metastasis and malignant effusions at pre-registration	Possibly due to alisertib
3	A	Grade 4 neutropenia; Grade 4 cerebral edema due to new brain metastasis identified prior to cycle 2	Probably due to alisertib; disease progression
4	B	Death due to acute respiratory failure (aspiration pneumonia) on cycle 1 day 8; on chronic oxygen supplementation at pre-registration	Unrelated
5	B	Grade 4 neutropenia after cycles 2-4; Grade 3 hyperbilirubinemia and hyponatremia after cycle 4; underlying pseudocirrhosis at pre-registration	Definitely due to alisertib; unrelated, due to underlying liver disease
**Protocol Changes:**			
• Changed pre-registration inclusion criteria for ECOG PS from 0-2 to 0-1 and repeated it in the registration criteria			
• Excluded patients with visceral crisis at pre-registration and added interval development of visceral crisis during pre-registration as registration exclusion criteria; included a definition for visceral crisis as “moderate-to-severe organ dysfunction as assessed by symptoms and signs, laboratory studies, and rapid progression of disease.”			
• Excluded patients with chronic oxygen supplementation requirement			
• Excluded patients with history of or evidence of brain metastasis			
• Revised registration total bilirubin from ≤1.5 upper limit normal (ULN) to total bilirubin ≤ULN with note that if it is out of normal range, then direct bilirubin must be ≤ ULN			

© 2023 Haddad TC et al. *JAMA Oncology.*
