# Supplementary material for: Evaluation of Alisertib Alone or Combined With Fulvestrant in Patients With Endocrine-Resistant Advanced Breast Cancer: The Phase 2 TBCRC041 Randomized Clinical Trial
Source: JAMA Oncol. 2023 Mar 9;9(6):815–24. doi: 10.1001/jamaoncol.2022.7949 (PMC9999287; doi:10.1001/jamaoncol.2022.7949)
Supplement: Supplement 3. — Data sharing statement [file jamaoncol-e227949-s003.pdf]

Data Sharing Statement

Haddad. Evaluation of Alisertib Alone or Combined With Fulvestrant in Patients With Endocrine-Resistant Advanced Breast Cancer. *JAMA Oncol*. Published March 09, 2023. doi:10.1001/jamaoncol.2022.7949

Data
Data available: Yes
Data types: Deidentified participant data
How to access data: The data generated in this study are available upon request from the corresponding author according to TBCRC policies. Haddad.Tufia@mayo.edu
When available: With publication

Supporting Documents
Document types: None

Additional Information
Who can access the data: to anyone requesting the data
Types of analyses: for any purpose
Mechanisms of data availability: with investigator support
Any additional restrictions: n/a
